# Post-transplant cyclophosphamide versus anti-thymocyte globulin after reduced intensity peripheral blood allogeneic cell transplantation in recipients of matched sibling or 10/10 HLA matched unrelated donors: final analysis of a randomized, open-label, multicenter, phase 2 trial

**DOI:** 10.1038/s41408-024-00990-3

**Published:** 2024-02-19

**Authors:** Eolia Brissot, Myriam Labopin, Helene Labussière, Gaelle Fossard, Patrice Chevallier, Thierry Guillaume, Ibrahim Yakoub-Agha, Micha Srour, Claude-Eric Bulabois, Anne Huynh, Sylvain Chantepie, Anne-Lise Menard, Marie-Therese Rubio, Patrice Ceballos, Rémy Dulery, Sabine Furst, Florent Malard, Didier Blaise, Mohamad Mohty

**Affiliations:** 1 Sorbonne Université, AP-HP, INSERM UMRs938, Paris, France ; Service d’Hématologie Clinique et de Thérapie Cellulaire, Hôpital Saint Antoine, AP-HP, Paris, France; 2 European Society for Blood and Marrow Transplantation Paris Study Office/CEREST-TC, Paris, France; 3 Hôpital Lyon Sud, Hospices Civils de Lyon, Pierre Bénite, France; 4 Hematology Department, Center Hospitalier Universitaire de Nantes, Nantes, France; 5 CHU Lille, Department of Hematology, Univ. Lille, INSERM U1286, Infinite, F-59000 Lille, France; 6 Department of Hematology, CHU de Grenoble, Grenoble, France; 7 CHU-Institut Universitaire du Cancer Toulouse Oncopole (IUCT-O), Toulouse, France; 8 Service d’Hématologie, Institut d’Hématologie de Basse-Normandie CHU de Caen, Caen, France; 9 Department of Hematology, Center Henri Becquerel, Rouen, France; 10 University Hospital of Nancy, Vandoeuvre-Les-Nancy, France; 11 Hematology Department, Saint-Eloi University Hospital, Montpellier, France; 12 Transplant and cellular immunotherapy program, Department of hematology, Institut Paoli Calmettes, Cancer research center of Marseille (CRCM), Aix-Marseille University (AMU), Marseille, France

**Keywords:** Haematological diseases, Clinical trial design

## Abstract

The use of post-transplantation cyclophosphamide (PTCy) for graft-versus-host disease (GVHD) prophylaxis is not established after reduced intensity conditioning (RIC) hematopoietic stem cell transplantation (HSCT) from fully matched donors. This was a randomized, open-label, multicenter, phase 2 trial. All patients received a RIC regimen with fludarabine, intravenous busulfan for 2 days (Flu-Bu2), and a peripheral blood stem cell (PBSC) graft from a matched related or 10/10 HLA-matched unrelated donor. Patients were randomly assigned to receive anti-thymocyte globulin (ATG) 5 mg/kg plus standard GVHD prophylaxis or PTCy 50 mg/kg/d at days +3 and +4 plus standard GVHD prophylaxis. The primary endpoint was the composite endpoint of GVHD- and relapse-free survival (GRFS) at 12 months after HSCT. Eighty-nine patients were randomly assigned to receive either PTCy or control prophylaxis with ATG. At 12 months, disease-free survival was 65.9% in the PTCy group and 67.6% in the ATG group (*P* = 0.99). Cumulative incidence of relapse, non-relapse mortality, and overall survival were also comparable in the two groups. GRFS at 12 months was 54.5% in the PTCy group versus 43.2% in the ATG group (*P* = 0.27). The median time to neutrophil and platelet count recovery was significantly longer in the PTCy group compared to the ATG group. Except for day +30, where EORTC QLQ-C30 scores were significantly lower in the PTCy compared to the ATG group, the evolution with time was not different between the two groups. Although the primary objective was not met, PTCy is effective for GVHD prophylaxis in patients receiving Flu-Bu2 conditioning with a PBSC graft from a fully matched donor and was well tolerated in term of adverse events and quality of life. This trial was registered at clinicaltrials.gov: NCT02876679.

## Introduction

The use of allogeneic hematopoietic stem cell transplantation (HSCT) is still increasing worldwide, mainly due to the development of reduced intensity conditioning (RIC) regimens, increase in age of patients and increasing stem cell sources [1]. However, graft-versus-host disease (GVHD) still represents a major limitation of HSCT. The combination of fludarabine and 2 days of busulfan (Flu-Bu2) is a widely used RIC regimen in many centers in Europe [2–6]. However, the best GVHD prophylaxis combination in the Flu-Bu2 RIC regimen has not yet been established. While the combination of cyclosporine A (CsA), and a short course of methotrexate (MTX) after transplantation is considered as the gold standard for GVHD prophylaxis after conventional myeloablative HSCT from HLA-identical siblings [7], there is no consensus on the optimal preventive regimen for GVHD prophylaxis after RIC HSCT [8]. In a retrospective study, similar outcomes in the group of patients who received MTX or mycophenolate mofetil (MMF) and CsA without ATG were observed, but this group had a higher risk of chronic (c) GVHD leading to worse survival. In the context of ATG-containing regimens, the addition of MMF or MTX to CsA did not reduce the risk of acute (a) GVHD, but significantly increased that of relapse incidence (RI), possibly as a consequence of the relatively reduced risk of cGVHD, leading to worse disease-free survival (DFS) and overall survival (OS) [9]. Considering the source of donors, the updated recommendations, based on several high-level evidence publications [10], suggest that rabbit anti-thymocyte globulin (ATG) or anti-T-lymphocyte globulin should be used for GVHD prophylaxis in patients undergoing matched unrelated donor (MUD) HSCT [11]. However, ATG delays immune reconstitution and has been shown to be associated with more infections, especially viral infections [12, 13]. On the other hand, post-transplant cyclophosphamide (PTCy) is now well established, successful, and widely utilized for GVHD prophylaxis after bone marrow (BM) haploidentical HSCT [14, 15]. The mechanism of action of PTCy has been described as inducing preferential elimination and clonal deletion of alloreactive T-cells [16]. Moreover, there is evidence supporting the importance of regulatory T-cells in mediating long-term post-transplant tolerance and GVHD control with PTCy [17]. However, BM is not the preferred source of stem cells after RIC HSCT, and the potential efficacy of PTCy on preventing GVHD when using PBSCs is still under debate. This point is of major concern, as PBSCs represent the main stem cell source of allogeneic cells worldwide. Results from two non-randomized phase 2 studies suggest that PTCy alone may not be the preferred GVHD prophylaxis following a RIC transplant with PBSCs [18, 19]. The incidences of grade II-IV and grade III-IV a GVHD were 45% and 27%, respectively, with a non-relapse mortality (NRM) of 36% at 1 and 2 years, suggesting the benefit of adding another immunosuppressive treatment in the PBSC transplantation setting. The hitherto inconclusive data highlight the need for a controlled trial in a standardized setting. We launched a phase 2 randomized clinical trial comparing at 12 months, the efficacy of PTCy versus ATG for GVHD prophylaxis in the setting of Flu-Bu RIC as determined by a composite endpoint of GVHD-free, relapse-free survival (GRFS), allogeneic PBSC transplantation. In this report, we present the final analysis of this study.

## Subjects and methods

### Study design and patients

This is a randomized, multicenter, open-label phase 2 trial comparing PTCy with standard ATG as GVHD prophylaxis in patients receiving a RIC regimen before HSCT from matched sibling donors (MSD) or 10/10-HLA MUD in 11 French medical centers. The trial was approved by the Comité de Protection des Personnes d’Aulnay-sous-Bois (France). Eligible patients were aged 18–70 years, diagnosed with a hematological malignancy, including acute myeloid or lymphoblastic leukemia, myelodysplastic syndrome, myeloproliferative disorder, chronic lymphocyte leukemia, and lymphoma, for which a RIC HSCT was indicated. Eligibility criteria for RIC HSCT included at least one of the following parameters: (i) patient age older than 50 years; (ii) heavily pre-treated patients who had received an autologous HSCT (auto-HSCT) or with more than two lines of chemotherapy before HSCT; and (iii) patients with poor performance status because of significant medical comorbidities [20] i.e., a Karnofsky score of at least 70. Only HLA-matched family donors and 10/10-HLA MUD were selected for the purpose of this study, donor selection was performed on the basis of high resolution (4 digit) typing of HLA-A, B, C, DRB1, DQB1. The complete eligibility criteria are in the protocol (Supplementary appendix 1). Mobilized PBSCs was the only stem cell source accepted. All patients gave informed consent.

### Randomization

Eligible patients were randomly assigned in a 1:1 ratio to receive either PTCy (experimental arm) or ATG (control arm). Allocation was done centrally. Patients were included and assigned to treatment up to 30 days before transplantation. Investigators had access to treatment assignments at their own centers. The statistician had access to data, in keeping with the need to report to the Data and Safety Monitoring Committee.

### Procedures

Fludarabine was administered intravenously over 30 min at a total dose of 150 mg/m^2^ divided into five daily doses of 30 mg/m^2^/day, from day −6 to day −2. Busulfan was infused once daily intravenously over 3 h at a dose of 130 mg/m^2^/day. In the experimental arm, cyclophosphamide (50 mg/kg/day) was given on days +3 and +4 post-transplant. In the control arm, rabbit ATG (Thymoglobuline®, Sanofi-Genzyme, Lyon, France) was given at 2.5 mg/Kg/day × 2 days on days −2 and −1. CsA was administered at a dose of 3 mg/kg/day by continuous intravenous infusion starting from day +5 in the experimental group and from day −3 in the control group, with doses adjusted to maintain a trough level of 200–400 ng/mL. CsA was changed to twice daily oral dosing as soon as it was tolerated. CsA was tapered over 4 weeks from day +62, if clinically possible. Oral MMF was given at a fixed dose of 2 g/day starting from day +5 in the experimental group and from day −3 in the control group. No treatment adjustment was performed for MMF. MMF was tapered over 4 weeks starting from day +35 if clinically possible. All supportive care was given in keeping with the local institutional practice.

Safety assessments included reports of adverse events (AEs) graded according to the Common Terminology Criteria Adverse Events version 4.02. Grading of aGVHD was performed according to the revised Glucksberg criteria [21]. cGVHD was recorded (as well as the requirement for a systemic immunosuppressive therapy) and the maximum grade achieved according to the NIH Consensus Criteria [22]. All Epstein-Barr Virus (EBV) and cytomegalovirus (CMV) reactivations requiring treatment had to be reported, as well as all cardiac AEs.

To assess health-related quality of life, patients completed the European Organization for Research and Treatment of Cancer (EORTC) Quality of Life Core Questionnaire (QLQ-C30) [23] and the Functional Assessment of Cancer Therapy-Bone Marrow Transplant (FACT-BMT) [24] at days −7 (baseline), +30, +90, +180 and +360.

### Outcomes

The primary endpoint was the composite endpoint of GRFS at 12 months after HSCT. In fact, it is well established that such composite endpoints acknowledge that both survival and rates of other critical events are important when testing new interventions. The Blood and Marrow Transplant Clinical Trials Network (BMT CTN) recognized the potential utility of a composite endpoint in HSCT trials [25]. GRFS after HSCT, including grade III-IV aGVHD, cGVHD requiring systemic treatment, relapse, or death, is a clinically meaningful one because it represents ideal recovery from HSCT (at 1 year) and a measure of cure without ongoing morbidity. Secondary endpoints were aGVHD, cGVHD, NRM, RI, DFS, OS, chronic GVHD-free, relapse-free survival (CRFS) and quality of life (QoL). CRFS was defined as survival in the absence of cGVHD and relapse. NRM was defined as death without evidence of relapse or progression. DFS was defined as survival with no evidence of relapse or progression. OS was defined as the time from HSCT to death, regardless of the cause. RI and NRM rates were estimated using cumulative incidence (CI) functions and considered as competing risks. For aGVHD and cGVHD, death and relapse were considered as competing events. For CI of serious AEs, death was considered as a competing event. Engraftment was defined as achieving an absolute neutrophil count greater than or equal to 0.5 × 10^9^/L for three consecutive days. Platelet engraftment was defined as independence from platelet transfusion for at least 7 days with a platelet count ≥20 × 10^9^/L.

### Statistical analysis

With 80 analyzable patients, the study design had 60% power to identify PTCy as superior to ATG when the GRFS at 1 year was 20% better than ATG at the one-sided significance level of 5% (two-sided level of 10%). The full analysis set included all patients who underwent HSCT for whom GRFS could be estimated. We also compared OS and DFS from time of randomization, in an intent-to-treat analysis. Probabilities of GRFS, DFS and OS were calculated using Kaplan–Meier estimates. Univariate analyses were performed using Gray’s test for CI and the log-rank test for GRFS, DFS and OS. The follow-up was censored at 1 year for 1-year comparisons. We also updated the follow-up of all patients in order to give the estimates at 3 years. Results of QoL after scoring were compared at each time point using the Mann-Whitney test and a generalized linear model was used to compare the evolution with time. Statistical analyses were performed with R version 4.0.1 (R Core Team (2020). R: A language and environment for statistical computing. R Foundation for Statistical Computing, Vienna, Austria. URL https://www.R-project.org/). This study was registered with ClinicalTrials.gov, identifier number: NCT02876679.

## Results

**Fig. 1:**
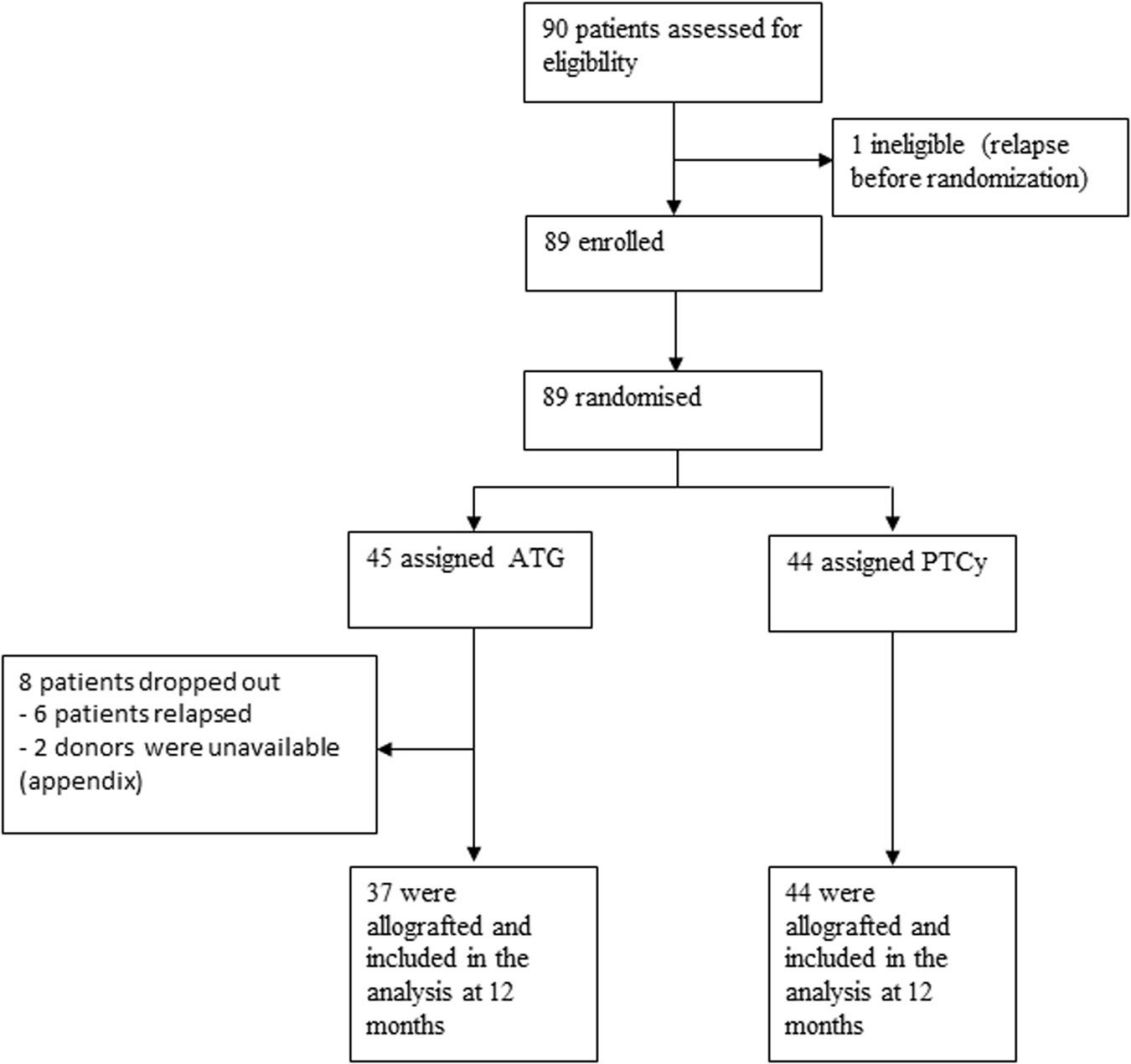
Study flowchart.

Between April 6, 2017, and October 10, 2019, a total of 90 patients from 11 centers were included. However, because one patient relapsed before randomization, only 89 patients were randomly assigned to receive either PTCy (44 patients) or control prophylaxis with ATG (45 patients) (Fig. 1). In the ATG-arm, only 37 patients underwent HSCT because six patients relapsed before transplant (4 cytologic relapses and two molecular relapses; 3 of these patients underwent HSCT with a sequential conditioning regimen chosen by the investigators) and two donors became unavailable (one unrelated donor became unavailable and one donor had an EBV replication) (Supplementary appendix 2-Table S1); in the PTCy group, 44 underwent HSCT. Overall, the median age was 64 years (range 21–71), and 69% patients were male. The baseline demographic and transplantation-related characteristics were well balanced between the two arms (Table 1). The median follow-up was 56 months in the PTCy group and 55 in the ATG group.

**Table 1:** Patient and transplant related characteristics.

		PTCy (*n* = 44)	ATG (*n* = 37)	*P* value
Median follow-up (months) [95% CI]		12 [11.97–12.16]	12 [11.93–12.3]	0.19
Patient age (years)	median (min-max) [IQR]	64.4 (36–71.1) [57.5–66.5]	64.3 (21.3–70.7) [59.4–67.6]	0.73
Patient gender	male	30 (68.2%)	26 (70.3%)	0.84
	female	14 (31.8%)	11 (29.7%)	
Recipient cytomegalovirus serostatus	Negative	19 (43.2%)	17 (47.2%)	0.29
	Positive	25 (56.8%)	17 (47.2%)	
	missing	0	3	
ECOG	0	25 (61%)	19 (57.6%)	0.78
	1	15 (36.6%)	12 (36.4%)	
	2	1 (2.4%)	2 (6.1%)	
	missing	3	4	
Diagnosis	acute myeloid leukemia	21 (47.7%)	17 (45.9%)	0.66
	acute lymphoblastic leukemia	2 (4.5%)	2 (5.4%)	
	multiple myeloma	1 (2.3%)	3 (8.1%)	
	lymphoma	8 (18.2%)	5 (13.5%)	
	chronic lymphocytic leukemia	1 (2.3)	0 (0%)	
	myelodysplastic syndrome	8 (18.2%)	8 (21.6%)	
	myeloproliferative disorder	1 (2.3%)	2 (5.4%)	
	other	2 (4.5%)	0	
Status at transplantation	diagnosis	1 (2.3%)	2 (5.4%)	0.15
	CR1	29 (65.9%)	17 (45.9%)	
	CR2	2 (4.5%)	7 (18.9%)	
	CR3	4 (9.1%)	2 (5.4%)	
	PR	4 (9.1%)	7 (18.9%)	
	R/R	4 (9.1%)	2 (5.4%)	
Time randomization-conditioning regimen (days)	median (min-max) [IQR]	7 (1–21) [2–9.5]	7 (1–23) [2–10]	0.72
Year of transplant	median (min-max)	2018 (2017–2019)	2018 (2017–2019)	0.54
Donor	matched related	17 (38.6%)	15 (40.5%)	0.86
	10/10 matched unrelated	27 (61.4%)	22 (59.5%)	
Donor sex	male	23 (52.3%)	21 (58.3%)	0.59
	female	21 (47.7%)	15 (41.7%)	
	missing	0	1	
Donor age (years)	median (min-max) [IQR]	38 (18–69) [27–54.2]	31 (21–69) [25–56]	0.66
	missing	2	2	
Donor cytomegalovirus serostatus	negative	25 (58.1%)	20 (57.1%)	0.93
	positive	18 (41.9%)	15 (42.9%)	
	missing	1	2	
Graft cell content				
CD34 × 10.6/Kg	median (min-max) [IQR]	6 (1.5–11.1) [4.7–7.6]	6.6 (2.6–10.9) [4.7–8.2]	0.42
	missing	1	1	
Associated IS	CsA +MMF	28 (63.6%)	24 (64.9%)	0.91
	CsA alone	16 (36.4%)	13 (35.1%)	

*CR* complete remission, *IS* immunosuppressive drug, *IQR* interquartile range, *PR* partial response, *R/R* relapsed or refractory, *PTCy* post-transplant cyclophosphamide, *ATG* anti-thymocyte globulin, *CsA* cyclosporine A, *MMF* mycophenolate mofetil, *ECOG* Eastern Cooperative Oncology Group.

**Fig. 2:**
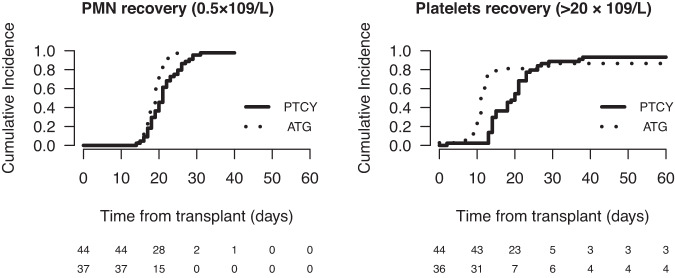
Polymorphonuclear neutrophil (PMN) and platelet engraftment after transplantation with post- cyclophosphamide (PTCy) or with anti-thymocyte globulin (ATG).

The median time to neutrophil recovery was significantly longer in the PTCy group at 21 days (range, 14–40) compared to 19 days in the ATG group (range, 14–27) (*P* = 0.01). Platelet count recovery was longer in the PTCy group, with a median time to achieve platelets >20 g/L of 20 days (range, 2–177) versus 11 days (range, 6–195) in the ATG group (*P* < 0.0001) (Fig. 2). Platelet numbers remained significantly lower at 1 year in the PTCy group compared to the ATG group. However, at 3 months and later, the number of platelet transfusions did not differ between the groups.

Table 2 shows the outcomes at 12 months. No statistically significant differences between the PTCy and ATG groups was observed.

**Table 2:** Outcomes at 1, 3, and 5 years.

		Relapse	NRM	DFS	OS	GRFS	CRFS	chronic GVHD	chronic GVHD requiring systemic treatment
1 year	PTCy (*n* = 44)	22.7% [11.6–36]	11.4% [4.1–22.8]	65.9% [50–77.8]	79.5% [64.4–88.8]	56.8% [41–69.9]	56.8% [41–69.9]	32.5% [18.5–47.2]	13.6% [5.4–25.6]
	ATG (*n* = 37)	27% [13.9–42]	8.1% [2–19.8]	64.9% [47.3–77.9]	81.1% [64.4–90.5]	40.5% [24.9–55.7]	40.5% [24.9–55.7]	36.1% [20.7–51.8]	24.3% [11.9–39.2]
	*P* value (censored at 1 year)	0.61	0.66	0.82	0.91	0.12	0.11	0.72	0.58
3 years	PTCy (*n* = 44)	27.3% [15–41]	11.4% [4.1–22.8]	61.4% [45.4–73.9]	72.7% [57–83.5]	47.7% [32.5–61.5]	47.7% [32.5–61.5]	32.5% [18.5–47.2]	18.2% [8.4–30.9]
	ATG (*n* = 37)	32.4% [18–47.7]	10.8% [3.3–23.3]	56.8% [39.4–70.8]	64.9% [47.3–77.9]	37.8% [22.6–53]	37.8% [22.6–53]	41.7% [25.2–57.4]	27% [13.8–42.1]
5 years	PTCy (*n* = 44)	27.3% [15–41]	18.6% [7.3–33.8]	54.2% [36.8–68.6]	60.3% [42.5–74.2]	43.2% [28.4–57.1]	43.2% [28.4–57.1]	32.5% [18.5–47.2]	22.7% [11.6–36.1]
	ATG (*n* = 37)	37.6% [20.8–54.4]	10.8% [3.3–23.3]	51.6% [33.2–67.2]	60.5% [42.1–74.7]	37.8% [22.6–53]	37.8% [22.6–53]	41.7% [25.2–57.4]	27% [13.8–42.1]
	*p* value (entire FU)	0.52	0.57	0.77	0.94	0.39	0.37	0.44	0.58

*NRM* non-relapse mortality, *DFS* disease-free survival, *OS* overall survival, *GRFS* graft-versus-host disease- free, relapse-free survival, *CRFS* chronic graft-versus-host disease-free, relapse-free survival, *GVHD* graft-versus-host disease, *PTCy* post-transplant cyclophosphamide, *ATG* anti-thymocyte globulin, *FU* follow-up.

**Fig. 3:**
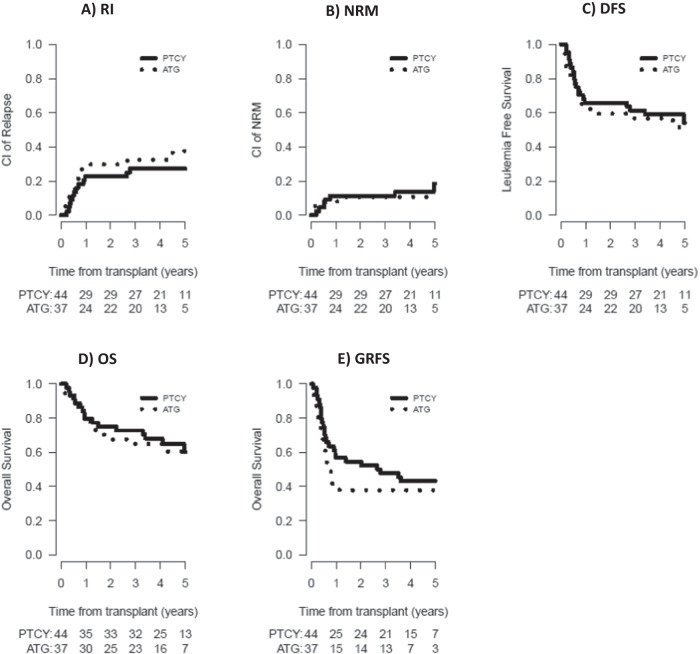
Clinical outcomes according to the use of post-transplant cyclophosphamide (PTCy) or anti-thymocyte globulin (ATG). Relapse incidence (RI) (**A**), non-relapse mortality (NRM) (**B**), disease-free survival (DFS) (**C**), overall survival (OS) (**D**), graft-versus-host disease-free, relapse-free survival (GRFS) (**E**) according to the use of post-transplant cyclophosphamide (PTCy) or anti-thymocyte globulin (ATG).

Likewise, at 5 years, there was no statistically significant difference in RI (27.3% vs. 37.6%, *P* = 0.52), CI of NRM (18.6% % vs. 10.8%, *P* = 0.57), DFS (54.2% vs. 51.6%, *P* = 0.77), OS (60.3% vs. 60.5%, *P* = 0.94), CRFS (43.2% vs. 37.8%, *P* = 0.37) or GRFS (43.2% vs. 37.8%, *P* = 0.39), between the PTCy and ATG groups, respectively (Fig. 3) (Table 2).

Finally, in an intent-to-treat analysis including all patients from randomization, 1-year DFS was 65.9% (95% CI, 50–77.8) vs. 62.2% (95% CI, 46.5–74.6) in the PTCy and ATG groups (*P* = 0.5), 1-year OS was 79.5% (95% CI, 64.4–88.8) vs. 73.3% (95% CI, 57.8–83.9), respectively (*P* = 0.41).

**Fig. 4:**
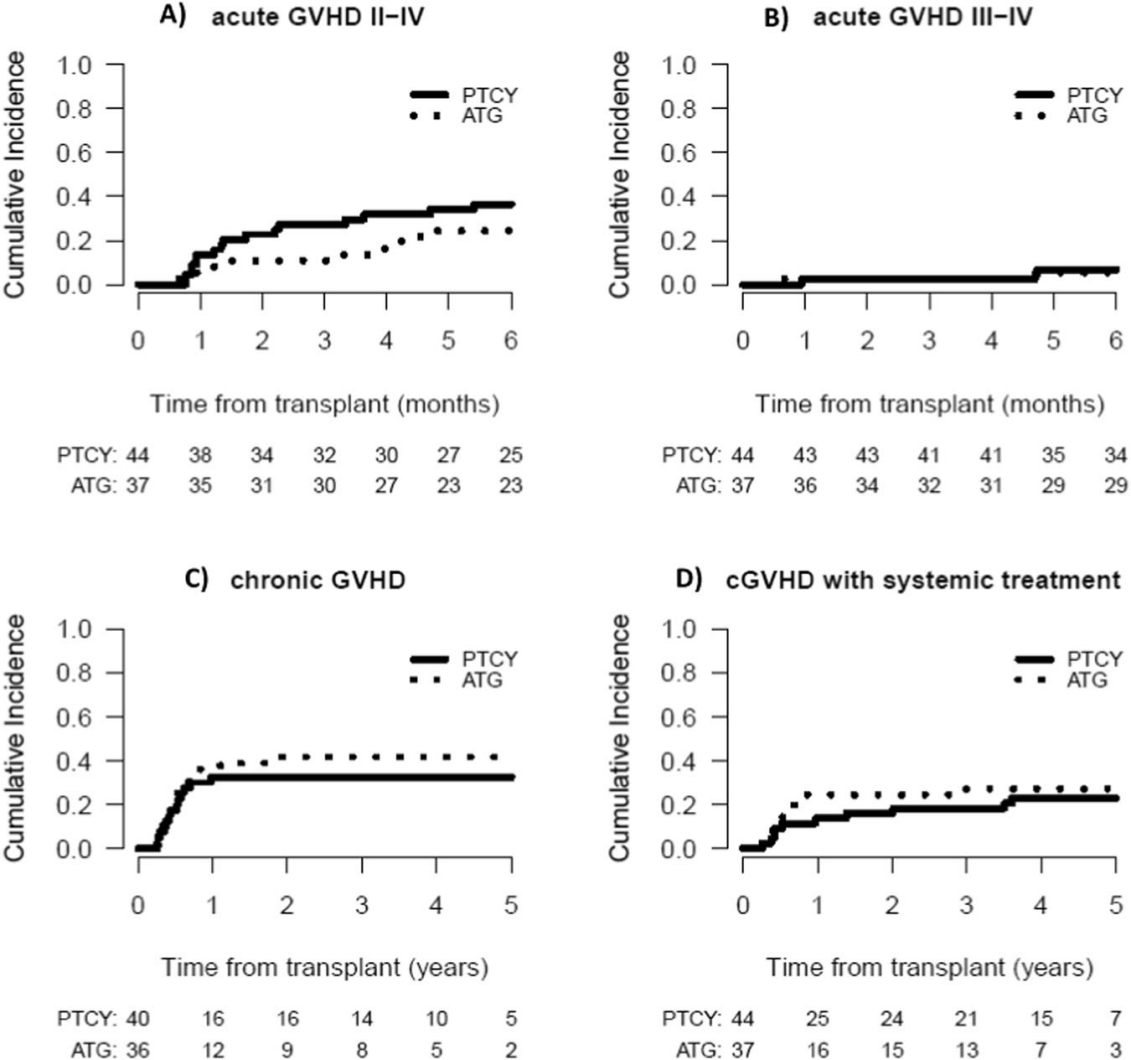
Cumulative incidence of GVHD. aGVHD grade II-IV(**A**), aGVHD grade III-IV(**B**), cGVHD (**C**), cGVHD requiring systemic treatment (**D**) according to the use of post-transplant cyclophosphamide (PTCy) or anti-thymocyte globulin (ATG).

At 6 months, no significant difference was observed in the CI of aGVHD grade II-IV between the PTCy and ATG groups, with 36.4% and 24.3%, respectively (*P* = 0.35) (Fig. 4). In patients who received PTCy, 6.8% developed aGVHD grade III-IV in comparison to 5.4% of those who received ATG (*P* = 0.81). The incidence, organ involvement and severity of aGVHD were similar in the two groups (Supplementary appendix 2-Table S2). At 1 year, the CI of cGVHD was 32.5% in the PTCy group and 36.1% in the ATG group, with no significant difference observed between the two groups (Fig. 4), and the CI of cGVHD requiring systemic treatment was 13.6% in the PTCy group and 24.3% in the ATG group (*P* = 0.58) (Table 2).

AEs numbered 37 in the PTCy group and 31 in the ATG group. Six and four patients developed a cardiac AE in the PTCy and ATG groups, respectively (Table 3). The CI of cardiac events at 1-year did not differ between the two groups with 11.4% (95%CI, 4.1–22.7) in the PTCy group and 8.1% (95%CI, 2–19.8) in the ATG group, *P* = 0.69. All cardiac complications occurred before 40 days except for two patients (1 in the PTCy group at day +323 and 1 in the ATG group at day +230) With respect to hemorrhagic cystitis, four events were observed in the PTCy group versus 1 in the ATG group (*P* = 0.37). Cystitis occurred within 20 days in the PTCy group and at day +201 in one patient who received ATG. EBV reactivation occurred in five patients in the PTCy group and in eight patients in the ATG group. The CI of EBV reactivation requiring treatment was 6.8% (95%CI, 1.7–16.9) and 21.6% (95%CI,10–36.1), *P* = 0.18. The CI for CMV reactivation requiring treatment did not differ between the groups: 29.5% (95%CI, 16.8–43.4) in PTCy and 32.4% (95%CI, 18–47.7) in the ATG group (*P* = 0.72). In the first month, 72.7% of patients experienced an infection in the PTCy group versus 59.5% in the ATG group (*p* = 0.21). Febrile neutropenia represented 78% of the infections in the PTCy group and 64% in the ATG group. Three cases of *Clostridium difficile* were reported in the PTCy group and two cases in the ATG group. Between the first and third month, 20 episodes of infection were reported in the PTCy group versus 19 in the ATG group (*p* = 0.73), it was mostly (70%) viral reactivation (in particular, CMV). In the PTCy group, the main cause of death was progression. Three patients died of GVHD, one of bacterial infection, one from hemorrhage. In the ATG group, progression was also the main cause of death with two patients dying of GVHD, one of pneumonia and one from hemorrhage (Supplementary appendix 2-Table S3).

**Table 3:** Serious adverse events.

		PTCY (*n* = 44)	ATG (*n* = 37)	*P*
EBV reactivation (requiring treatment)	No	39 (88.6%)	29 (78.4%)	0.21
	Yes	5 (11.4%)	8 (21.6%)	
CMV reactivation (requiring treatment)	No	31 (70.5%)	25 (67.6%)	0.78
	Yes	13 (29.5%)	12 (32.4%)	
CARDIAC Events (Atrial Fibrillation/Arrhythmia/Unspecified Cardiac Disease)	No	38 (86.4%)	33 (89.2%)	0.75
	Yes	6 (13.6%)	4 (10.8%)	
Hemorrhagic CYSTITIS (BK Virus associated hemorrhagic cystitis/Non-BK Virus associated hemorrhagic cystitis)	No	40 (90.9%)	36 (97.3%)	0.37
	Yes	4 (9.1%)	1 (2.7%)	

*ATG* anti-thymocyte globulin, *CMV* Cytomegalovirus *EBV* Epstein Barr Virus, *PTCy* post-transplant cyclophosphamide.

Except for day +30 where EORTC QLQ-C30 scores were significantly lower in the PTCy compared to the ATG group (*P* = 0.01), there were no significant differences between the two groups at days +90, +180, and +360. There was a suggestion of trend towards a lower FACT-BMT score at day +30 in the PTCy group (*P* = 0.051), but the scores were not significantly different at the other time points. The evolution with time was not different between the two groups (*P* = 0.24 and *P* = 0.70 for the EORTC functional score and FACT-BMT score, respectively).

## Discussion

Despite progress in prophylaxis regimens, GVHD remains a major complication post-HSCT, leading to increased morbidity, mortality, and altered QoL. The differential impacts of PTCy and ATG are an important area of clinical and biological research. However, only retrospective studies with different aims have been conducted. This randomized phase 2 trial did not show significant differences between PTCy and ATG in the acute and cGVHD incidence in patients who underwent RIC HSCT with an MSD or a 10/10 HLA MUD. Even with over 4 years of follow-up, the absence of a statistically significant difference persists between the two groups. Considering donor type, the results are in accordance with a retrospective study from the EBMT on 174 and 1452 patients transplanted with a 10/10 HLA MUD, receiving PTCy and ATG, respectively, in which no significant difference between the PTCy and ATG groups was observed for incidence of grade II-IV aGVHD [26]. Bailen et al. have reported on 60 patients undergoing a matched or 9/10 mismatched unrelated donor (MMUD) HSCT with ATG-based prophylaxis combined with MTX-CsA, and 72 using a PTCy-based regimen [27]. PTCy showed a reduction in the rate of aGVHD but it is worth noting that 9/10 MMUDs were included. Recently, Bolanos-Meade *et al*. published the results of a phase 3 trial that randomized PTCy-tacrolimus-MMF or tacrolimus-MTX in patients who underwent HSCT from an HLA-matched related donor or a matched or 7/8 mismatched unrelated donor after a RIC regimen [28]. GRFS was significantly longer in the group who received PTCy, and the analysis showed a significantly lower hazard of grade III or IV aGVHD and of cGVHD with the PTCy-prophylaxis regimen than with the standard-prophylaxis regimen. In the phase 3 HOVON 96 trial, PTCy associated with CsA was compared to mycophenolic acid and CsA in patients with a RIC regimen [29]. This trial showed that PTCy was superior to CsA-MMF with respect to GFRS at 1 year. These important results emphasize the beneficial effect of PTCy in HSCT, more significantly than in the haploidentical setting. However, ATG was not included in these trials which is the standard GVHD prophylaxis in most of European countries for RIC regimens. In a retrospective study comparing the outcomes of adults with acute myeloid leukemia undergoing HSCT from HLA-MSD after the use of PTCy or ATG, no differences were observed at 1 year, in the transplantation outcomes, except for cGVHD which was significantly lower in the ATG group [30]. Although there was heterogeneity in terms of conditioning regimens and association of immunosuppressive drugs, the DFS and RI were comparable in both groups. These are important data especially considering that RIC HSCT mainly relies on the development of an immunological graft-versus-leukemia (GVL) effect, in contrast to a myeloablative conditioning regimen [31]. It is now established that the effects of ATG on RIC are dose-dependent, and that intermediate doses of ATG between 4 and 6 mg/kg seem to prevent GVHD optimally while sparing the GVL effect [32]. In our trial, using PTCy at the dose of 50 mg/kg/d on days +3 and +4 post-transplantation resulted in similar DFS and RI incidence, suggesting a retention of the GVL effect.

This trial did not find a significant difference in the safety profiles of PTCy and ATG. The cardiac toxicity profile of PTCy compared with non-PTCy GVHD prophylaxis is still under debate [33]. In our trial, 14% developed a cardiac AE in the PTCy group versus 8% in the ATG group, a statistically non-significant difference. Yeh et al. performed a retrospective analysis to evaluate cardiac toxicities after HLA-matched HSCT [34]. They found that baseline cardiac comorbidities were associated with a higher incidence of cardiac toxicities after HSCT, but PTCy-based GVHD prophylaxis did not appear to impact their development. Viral reactivation is an important issue, especially during the first few months post-transplant. ATG is a well-defined risk factor for EBV reactivation and EBV post-transplantation lymphoproliferative disorder [35]. Goldsmith et al. reported that PTCy, regardless of donor, was associated with a higher incidence of CMV infection [36]. However, we did not find significant differences for EBV and CMV reactivation between the two groups. This could reflect the low intensity of the Flu-Bu2 conditioning regimen with a faster immune reconstitution. Likewise, the rate of hemorrhagic cystitis was similar after PTCy and ATG.

Finally, the QoL study pointed to a significantly lower score for the PTCy group initially, at day +30; however, at months 3, 6, and 12, no difference was observed between the two groups.

Limitations of the study included heterogeneity of patients’ hematological diseases. RIC regimens typically have less antineoplastic activity but have limited toxicity and are thus better tolerated by patients who are not eligible for myeloablative conditioning. In our cohort, 20 patients were not in complete remission at transplant, of whom eight were treated for B-mature lymphoid malignancies or Hodgkin lymphoma and, therefore, had already received several lines of chemotherapy. It could explain why investigators decided to perform a Flu-Bu2 conditioning regimen, regarding the numerous previous lines of treatment and the median age of the cohort. However, the main objective of this study was to compare PTCy and ATG as GVHD prophylaxis and their impact on GRFS prevention regardless of the diagnosis. The distribution of diseases was well balanced between the two groups, however, the limited size of the cohort may mask subtle differences in patient characteristics such as disease status at transplant. Finally, the survival outcomes of DFS, RI, NRM, and OS were comparable between the two groups, but they should be confirmed in a larger study. We allowed 1 month between randomization and HSCT which led to the exclusion of eight patients, by chance, all in the ATG-arm, the main reason was relapse pre-transplant that modified the conditioning regimen. However, the characteristics of the population in each arm were comparable. Finally, as it was a phase 2 study, we were only able to include 40 patients per group therefore, the statistical power planned for in the protocol was obviously limited.

Although the primary objective was not met, the important results of this multicenter prospective study highlight some practical points. ATG has been extensively recognized as the ‘standard of care’ for GVHD prophylaxis in MSD and MUD HSCT [37]. However, it carries some limitations: it is not available everywhere, it is rather expensive, difficult to administer, and is often linked by investigators to the generation of opportunistic infections. The alternative drug, cyclophosphamide, is comparatively low-cost, widely available, and easy to use. Due to its significant success in the control of GVHD in the ‘difficult’ haploidentical HSCT setting, the question of whether it would be advantageous in MSD and MUD transplants is of paramount importance, particularly in centers where the routine use of ATG is precluded for the stated reasons. Despite some retrospective studies showing that PTCy is indeed effective in the latter setting, no conclusive data on its advantage or disadvantage over ATG was observed. Therefore, our randomized trial results demonstrate that the effectiveness and safety profile of the two drugs appear to be very similar, opening up a basis for an informed choice and paving the way for future studies.

### Supplementary information

supplementary appendix 1

supplementary appendix 2

## Data Availability

Data supporting the findings of this study including de-identified patient data are available after final completion of the trial report and are shared according to data sharing guidelines upon reasonable request to the corresponding author.
