# Supplementary material for: Post-transplant cyclophosphamide versus anti-thymocyte globulin after reduced intensity peripheral blood allogeneic cell transplantation in recipients of matched sibling or 10/10 HLA matched unrelated donors: final analysis of a randomized, open-label, multicenter, phase 2 trial
Source: Blood Cancer J. 2024 Feb 19;14(1):31. doi: 10.1038/s41408-024-00990-3 (PMC10876658; doi:10.1038/s41408-024-00990-3)
Supplement: Supplementary file 1 — supplementary appendix 1 [file 41408_2024_990_MOESM1_ESM.pdf]

A randomized phase IIb study of cyclophosphamide (Cy) versus anti-thymocyte globulin (ATG) for the prophylaxis of graft-versus-host disease (GVHD) after reduced-intensity conditioning allogeneic peripheral blood stem cell transplantation-

ATG-Cy GVHD

BIOMEDICAL RESEARCH PROTOCOL RELATING TO A MEDICINAL PRODUCT FOR HUMAN USE

Version N°6 of 28/02/2018
Project Code: P150955 / EUDRACT N°: 2016-002129-12

Coordinating Investigator: Pr Mohamad MOHTY
Saint-Antoine Hospital
Service d'hématologie clinique et thérapie cellulaire
Tel: +33 149 28 26 20
Email: mohamad.mohty@inserm.fr

Sponsor: AP-HP and by delegation: Clinical Research and Development Department (DRCD)
Hôpital Saint-Louis
1, avenue Claude Vellefaux
DRCD-Siège project referent: **Maud JACUBERT**
Tel.: 01 44 84 17 80
Email: maud.jacubert@aphp.fr

Methodology, statistical analysis: Dr Myriam LABOPIN
Saint-Antoine Hospital
Tel: 33 (0)1 40 01 13 14
Email: myriam.labopin@upmc.fr

Entity responsible for monitoring research:
Unité de Recherche Clinique (URC) du GH HUEP
Saint-antoine Hospital
184 rue du Faubourg Saint Antoine
75012 Paris
DRCD-URC (Clinical Research Unit) project referent:
Amel TOUATI
Tel: 01 49 28 22 02
Email: amel.touati@aphp.fr

Entity responsible for investigational products circuit :
Département Essais cliniques de l'AGEPS
Pharmacist: **Dr Blandine LEHMANN / Caroline Egon**
Chrystelle Frappesauce-Hereng
Tel: 01 46 69 14 02
Email: blandine.lehmann@aphp.fr / caroline.egon@aphp.fr
chrystelle.frappesauce-hereng@aphp.fr

Clinical Research and Development Department (DRCD)
Hôpital Saint Louis 75010 PARIS

SIGNATURE page for a biomedical research PROTOCOL

Research Code: P150955 / EUDRACT N° 2016-002129-12

Title: **A randomized phase IIb study of cyclophosphamide (Cy) versus anti-thymocyte globulin (ATG) for the prophylaxis of graft-versus-host disease (GVHD) after reduced-intensity conditioning allogeneic peripheral blood stem cell transplantation – ATG-Cy GVHD**

Version N°6 of: 28/02/2018

The research will be carried out in accordance with the protocol, with current good practices and with the legislative and regulatory provisions in force.

Coordinating Investigator:
Pr Mohamad MOHTY

Saint-Antoine Hospital
Service d’hématologie clinique et thérapie cellulaire

Date: ……………/………/………..
Signature:

Sponsor
AP-HP
and by delegation: Clinical Research and Development Department (DRCD)

Date: ……………/………/………..
Signature:

The research received a favourable opinion from the CCP Ile-De-France X on 08/11/2016 and authorisation from the ANSM on 12/10/2016

CONTENTS

1 SCIENTIFIC JUSTIFICATION FOR THE RESEARCH ... 11
1.1 HYPOTHESIS FOR THE RESEARCH ... 11
1.2 DESCRIPTION OF KNOWLEDGE RELATING TO THE PATHOLOGY IN QUESTION ... 12
1.3 DESCRIPTION OF THE POPULATION TO BE STUDIED AND JUSTIFICATION FOR THE CHOICE OF PARTICIPANTS ... 13
2 OBJECTIVES ... 13
2.1 PRIMARY OBJECTIVE ... 13
2.2 SECONDARY OBJECTIVES ... 13
2.3 OBJECTIVE OF ANY POSSIBLE ANCILLARY RESEARCH ... 13
3 PLAN FOR THE RESEARCH - DESCRIPTION OF RESEARCH METHODOLOGY ... 13
3.1 CONCISE DESCRIPTION OF THE PRIMARY AND SECONDARY ASSESSMENT CRITERIA ... 14
3.1.1 Primary assessment criterion ... 14
3.1.2 Secondary assessment criteria ... 14
3.2 EXPERIMENTAL PLAN ... 14
3.3 NUMBER OF CENTRES PARTICIPATING ... 14
3.4 IDENTIFICATION OF THE SUBJECTS ... 14
3.5 RANDOMISATION ... 14
4 PROCEDURE FOR THE RESEARCH ... 14
4.1 SELECTION PERIOD ... 14
4.2 INCLUSION / RANDOMIZATION VISIT (J-30, J0) ... 15
4.3 FOLLOW-UP VISITS ... 17
4.4 END OF RESEARCH VISIT ... 17
4.5 EXPECTED LENGTH OF PARTICIPATION AND DESCRIPTION OF THE CHRONOLOGY AND DURATION OF THE RESEARCH. ... 18
4.6 TABLE OR DIAGRAM SUMMARISING THE CHRONOLOGY OF THE RESEARCH - DISTINCTION BETWEEN CURRENT PRACTICE AND RESEARCH ... 18
4.7 BIOLOGICAL COLLECTION ... 19
4.8 TERMINATION RULES ... 19
4.8.1 Criteria and methods for prematurely terminating the research treatment ... 19
4.8.2 Follow-up of the subjects after the premature termination of treatment ... 20
4.8.3 Methods for replacing subjects, if applicable ... 20
4.8.4 Terminating part or all of the research ... 20
5 PATIENTS RECRUITMENT ... 21
5.1 RECRUITMENT METHODS ... 21
5.2 EXPECTED NUMBER OF PATIENTS ELIGIBLE IN THE CENTRES ... 21
6 TREATMENT ADMINISTERED TO RESEARCH PARTICIPANTS ... 22
6.1 DESCRIPTION OF THE EXPERIMENTAL MEDICATIONS ... 22
6.1.1 Cyclophosphamide ... 22
6.2 DESCRIPTION OF THE NON-EXPERIMENTAL TREATMENT ... 24
6.3 DESCRIPTION OF THE TRACEABILITY ELEMENTS THAT ACCOMPANY THE EXPERIMENTAL MEDICATIONS 25
7 ASSESSMENT OF EFFICACY ... 26
7.1 DESCRIPTION OF PARAMETERS FOR ASSESSING EFFICACY ... 26
7.1.1 Primary Efficacy Outcome Variable ... 26
7.1.2 Secondary Efficacy Outcome Variables ... 26
7.2 ANTICIPATED METHODS AND TIMETABLE FOR MEASURING, COLLECTING AND ANALYSING THE PARAMETERS FOR ASSESSING EFFICACY ... 26
7.2.1 Primary Efficacy Outcome ... 26
7.2.2 Secondary Efficacy Outcome ... 26
8 SPECIFIC RESEARCH COMMITTEES ... 27

8.1 STEERING COMMITTEE 27
8.2 SAFETY DATA MONITORING COMMITTEE 27

9 SAFETY ASSESSMENT - RISKS AND RESTRICTIONS ADDED BY THE RESEARCH 28

9.1 DESCRIPTION OF PARAMETERS FOR ASSESSING SAFETY 28
9.2 ANTICIPATED METHODS AND TIMETABLE FOR MEASURING, COLLECTING AND ANALYSING THE PARAMETERS FOR ASSESSING SAFETY (SEE TABLE 10.2) 28
9.3 PROCEDURES IN PLACE FOR RECORDING AND REPORTING ADVERSE EVENTS 29
9.3.1 Definitions 29
9.3.2 The investigator's roles 30
9.3.3 Specific features of the protocol 31
9.3.4 Procedures and deadlines for notifying the sponsor 32
9.3.5 Period for notifying the sponsor 33
9.3.6 The sponsor's roles 33

10 DATA MANAGEMENT 37

10.1 DATA COLLECTION METHODS 37
10.2 IDENTIFICATION OF DATA COLLECTED DIRECTLY IN THE CRFS AND THAT WILL BE CONSIDERED AS SOURCE DATA 37
10.3 RIGHT TO ACCESS SOURCE DATA AND DOCUMENTS 37
10.3.1 Access to data 37
10.3.2 Source documents 37
10.3.3 Data confidentiality 38
10.4 DATA PROCESSING AND STORAGE OF DOCUMENTS AND DATA 38
10.4.1 Identification of the manager and the location(s) for data processing 38
10.4.2 Data entry 38
10.4.3 Data processing (CNIL, the French Data Protection Authority) in France 38
10.4.4 Archival 38
10.5 OWNERSHIP OF THE DATA 39

11 STATISTICAL ASPECTS 39

11.1 DESCRIPTION OF STATISTICAL METHODS TO BE USED INCLUDING THE TIMETABLE FOR THE PLANNED INTERIM ANALYSES 39
11.2 CALCULATION HYPOTHESES FOR THE NUMBER OF SUBJECTS REQUIRED AND THE RESULT 39
11.3 SPECIFY IF SUBJECTS WHO LEAVE THE RESEARCH PREMATURELY WILL BE REPLACED AND IN WHAT PROPORTION. 40
11.4 ANTICIPATED LEVEL OF STATISTICAL SIGNIFICANCE 40
11.5 STATISTICAL CRITERIA FOR TERMINATION OF THE RESEARCH. 40
11.6 METHOD FOR TAKING INTO ACCOUNT MISSING, UNUSED OR INVALID DATA 40
11.7 MANAGEMENT OF MODIFICATIONS MADE TO THE ANALYSIS PLAN FOR THE INITIAL STRATEGY 40
11.8 SELECTION OF POPULATIONS 40

12 QUALITY CONTROL AND ASSURANCE 40

12.1 GENERAL ORGANISATION 40
12.1.1 Strategy for opening the centres 41
12.1.2 Level of centre monitoring 41
12.2 QUALITY CONTROL 41
12.3 CASE REPORT FORM 41
12.4 MANAGEMENT OF NON-COMPLIANCES 41
12.5 AUDITS/INSPECTIONS 42
12.6 PRIMARY INVESTIGATOR'S COMMITMENT TO ASSUME RESPONSIBILITY 42

13 ETHICAL AND LEGAL CONSIDERATIONS 42

13.1 RESPONSIBILITIES OF INVESTIGATORS 42
13.2 METHODS FOR OBTAINING INFORMATION AND CONSENT FROM RESEARCH PARTICIPANTS 42
Special case: Mention of the possibility for the investigator of withholding certain 43
13.3 SUBJECT PROHIBITED FROM PARTICIPATING IN ANOTHER RESEARCH OR AN EXCLUSION PERIOD ANTICIPATED AFTER THE RESEARCH, IF APPLICABLE 43
13.4 COMPENSATION FOR SUBJECTS 43

13.5 REGISTRATION ON THE NATIONAL REGISTER OF SUBJECTS PARTICIPATING IN BIOMEDICAL RESEARCH RELATING TO THE PRODUCTS LISTED IN ARTICLE L. 5311-1 OF THE FRENCH PUBLIC HEALTH CODE 43
13.6 LEGAL OBLIGATIONS 43
13.6.1 The sponsor's role *43*
13.7 REQUEST FOR AN OPINION FROM THE COMITÉ DE PROTECTION DES PERSONNES (CPP, ETHICAL REVIEW BOARD)43
13.8 REQUEST FOR AUTHORISATION TO ANSM 43
13.9 COMMITMENT TO COMPLIANCE WITH THE MR 001 "MÉTHODOLOGIE DE REFERENCE" 43
13.10 MODIFICATIONS TO THE RESEARCH 43
13.11 FINAL RESEARCH REPORT 44

14 FUNDING AND INSURANCE 44
14.1 FUNDING SOURCE 44
14.2 INSURANCE 44

15 PUBLICATION RULES 44
15.1 MENTION OF THE AFFILIATION OF AP-HP FOR PROJECTS SPONSORED OR MANAGED BY AP-HP 44
15.2 MENTION OF THE AP-HP MANAGER (DRCD) IN THE ACKNOWLEDGEMENTS OF THE TEXT 44
15.3 MENTION OF THE FINANCIER IN THE ACKNOWLEDGEMENTS OF THE TEXT 44

16 BIBLIOGRAPHY 44

17 LIST OF APPENDIX 48
Appendix 1: SUMMARY OF PRODUCT CHARACTERISTICS 48
Appendix 2: GVHD STAGING (Przepiorka et al., 1995) 48
Appendix 3: GVHD GRADING (Przepiorka et al., 1995) 48
Appendix 4: Rule of 9s for body surface area. 48
Appendix 5: Signs and symptoms of cGvHD (2014 NIH consensus criteria) (Jagasia et al., 2015) 50
Appendix 6: Organ scoring of cGvHD (2014 NIH consensus criteria) (Jagasia et al., 2015) 51
Appendix 7: Performans status (OMS) 53
Appendix 8: Karnofsky Performance Scale Index 54
Appendix 9: EORTC QLQ-C30 (European Organisation for Research and Treatment of Cancer Quality of Life Questionnaire-Core 30) 55
Appendix 10: FACT-BMT (Functional Assessment of Cancer Therapy - Bone Marrow Transplant 57

SUMMARY

Full title	A randomized phase IIb study of cyclophosphamide (Cy) versus anti-thymocyte globulin (ATG) for the prophylaxis of graft-versus-host disease (GVHD) after reduced-intensity conditioning allogeneic peripheral blood stem cell transplantation
Acronym	ATG-Cy GVHD
Coordinating Investigator	Pr Mohamad MOHTY
Sponsor	Assistance Publique – Hôpitaux de Paris
Scientific justification	Allogeneic stem cell transplantation (allo-SCT) is a well-established therapy for different hematologic malignancies. Reduced-intensity conditioning (RIC) regimens can decrease the rate of toxicity/mortality in elderly patients, or in patients with poor medical condition. GVHD prophylaxis remains a challenging task after allo-SCT. The Flu-ivBu combination is a widely used RIC regimen, endorsed by EMA since July 2014. ATG in combination with cyclosporine-A ±mycophenolate mofetil is the backbone for GVHD prophylaxis in this setting. ATG can prevent GVHD with a good efficacy, but at the cost of a higher toxicity and profound immunosuppression, calling for more effective therapies. The most widely used RIC regimen in France incorporates fludarabine (Flu), intermediate doses of IV-busulfan (Bu) and anti-thymocyte globulins (ATG). While the use of ATG can prevent severe acute and chronic GVHD after allogeneic peripheral blood stem cell (PBSC) transplantation from both HLA-identical sibling and unrelated donors, some data suggested that in-vivo T-cell depletion with ATG in the RIC setting may induce a higher risk of disease relapse. Also, ATG induces profound immune suppression and increase incidence of opportunistic infections, especially EBV-related complications (relative risk=4.9; 95% CI[1.1-21.0]; P=0.03).<br>On the other hand, high-dose post-transplantation cyclophosphamide (PTCy) was developed to facilitate HLA-haploidentical allo-SCT using unmanipulated bone marrow (BM) cells. PTCy was effective in preventing both acute and chronic GVHD given its capacity to preferentially eliminate allo-reactive T cells and preserve regulatory T cells, both of which impact allogeneic immune reactions. Subsequently, the efficacy of PTCy as sole GVHD prophylaxis after myeloablative conditioning when using BM was also shown. However, BM is not the preferred source of stem cells after RIC allo-SCT, and the potential efficacy of PTCy on preventing GVHD when using PBSCs (which is the most frequently used source of allogeneic cells worldwide) is debated.<br>The advent of PTCy therapy is nowadays on the cutting edge. Thus, the potential efficacy (and cost-effectiveness) of PTCy for GVHD prophylaxis may have a major ATG sparing potential. A recent single centre phase 2 study (n=49) suggested that PTCy alone may not be the preferred GVHD prophylaxis following a RIC transplant with PBSCs. Indeed, A matched cohort analysis compared outcomes to tacrolimus/methotrexate GVHD prophylaxis and indicated higher rates of acute GVHD grade II to IV (46% versus 19%; hazard ratio [HR], 2.8; P =0..02) and treatment-related mortality (HR, 3.3; P =0.035) and worse overall survival (HR, 1.9; P=0..04) with post-CY. Interpretation of the above non-randomized data is further complicated by heterogeneity (related and unrelated donors, BM and PBSC as stem cell source, different conditioning regimen), highlighting the need for a controlled randomized trial in a standardized setting.
Primary objective and assessment criterion	Primary objective:<br>To compare at 12 months the efficacy of post-transplant cyclophosphamide (PTCy) versus anti-thymocyte globulin (ATG) for GVHD prophylaxis in the setting of Fludarabine-Busulfan reduced-intensity conditioning (RIC) as determined by a composite endpoint of GVHD-free, relapse-free survival (GRFS) allogeneic peripheral blood stem cell transplantation.<br><br>Primary assessment criteria:

	The primary endpoint of the trial will be the assessment of a composite endpoint of graft–versus-host disease-free, relapse-free survival (GRFS) at 12 months after allogeneic stem cell transplantation.
Secondary objectives and assessment criteria	Secondary endpoint:<br>• To evaluate the occurrence of grade 2-4 and grade 3-4 severe acute GVHD within the first 6 months after transplantation.<br>• To evaluate the occurrence of chronic GVHD within the first 12 months after transplantation.<br>• To evaluate non-relapse mortality within the first 12 months after transplantation.<br>• To evaluate disease-free and overall survival at 12 months after transplantation.<br>• To evaluate the Quality of Life (QoL) in both treatment arms.<br>• Increase the knowledge on the mechanism of action of PTCy and ATG in GVHD prophylaxis by descriptive immune recovery studies.<br><br>Secondary assessment criteria:<br>• Cumulative incidence of grade 2-4 and grade 3-4 severe acute GVHD, according to the Glucksberg criteria revised by Przepiorka et al., within the first 6 months after transplantation.<br>• Cumulative incidence of chronic GVHD as assessed by NIH Consensus Criteria within the first 12 months after transplantation.<br>• Cumulative incidence of non-relapse mortality within the first 12 months after transplantation.<br>• Disease-free and overall survival at 12 months after transplantation.<br>• Quality of Life (QoL) in both treatment arms at D-7,D30, D90, D180 and D360.<br>• Descriptive immune recovery studies (lymphocytes –including regulatory cells- and dendritic cells subsets) related to the use of PTCy versus ATG in GVHD prophylaxis.
Experimental design	This is a phase IIB randomized, multicenter, active comparator controlled, parallel group study.<br>Patients will be randomized 1:1 to receive either, the fludarabine, IV busulfan conditioning and ATG for GVHD prophylaxis (“standard of care” arm) or the fludarabine, IV busulfan conditioning without ATG but with post-transplant cyclophosphamide (PTCy; “experimental arm”).
Population involved	Women and men with a hematologic malignancy for which a reduced-intensity conditioning allo-SCT is indicated
Inclusion criteria	- Patients aged between 18 and 70 years<br>- Presence of a hematologic malignancy for which a reduced-intensity conditioning allo-SCT is indicated (eligibility criteria for RIC allo-SCT) include at least one of the following parameters: (i) patient age older than 50 years; (ii) heavily pre-treated patients who received an autologous hematopoietic SCT (auto-SCT) or with more than 2 lines of chemotherapy before allo-SCT; and (iii) patients with poor performance status because of significant medical comorbidities as described by Sorror et al.<br>- Karnofsky index ≥ 70%<br>- Availability of a sibling or unrelated stem-cell donor (10/10-HLA matched unrelated donor)<br>- Efficient contraceptive method during the whole length of the treatment and in accordance with SmPC of the administered products<br>- Written informed consent.<br>- Affiliation to a social security system (recipient or assign)
Non-inclusion criteria	The presence of a single non-inclusion criterion makes a patient unable to enter the protocol:<br>- Creatinine clearance less than 30 mL/min<br>- Bilirubin or amino-transferases above 3X upper normal limit<br>- Cardiac ejection fraction less than 40%<br>- Pulmonary impairment with <50% lung carbon monoxide diffusing capacity (DLCO)<br>- Known hypersensitivity or contraindication to the use of post-transplant Cy and ATG<br>- Any circumstance that precludes the use of the drugs involved in the protocol<br>- Pregnancy or breast-feeding women<br>- Patients allografts
Experimental	• In the experimental group:

treatment	Cyclophosphamide: 50mg/Kg/day IV at D+3 and D+4<br><br>• In the control group:<br>Anti-thymocyte globulin (ATG - Thymoglobuline®) 2.5 mg/Kg/day IV for 2 consecutive days (day -2 and -1)
Non experimental treatment	• Fludarabine<br>• Busulfan<br>• Cyclosporine A<br>• Mycophenolate Mofetil (MMF)<br>• Mesna<br>• Supportive care
Treatment regimen	Patients should be included in the month prior to D0 of allo-SCT. Patients will be randomized 1:1 in the “experimental group” or in the “control group”.<br>After registration for the study, the following treatment plan is to be followed:<br><u>a- RIC regimen</u><br>-30 mg/m^2/day IV fludarabine for 5 days (day-6 to day-2)<br>-130 mg/m^2/day IV busulfan once daily for 2 days (day -4 and -3)<br><br><u>b- Graft infusion on D0</u>: only peripheral blood stem cells will be accepted; CD34 target dose is 4 × 10^6 per Kg body weight, with a minimum of 2 × 10^6 per Kg body weight.<br><br><u>c- GVHD prophylaxis</u><br>➔ <u>in the experimental group</u>:<br>Cyclophosphamide: 50mg/Kg/day IV at D+3 and D+4<br>Cyclosporine A (CsA) 3mg/kg/day IV from day +5<br>In case of unrelated donor: Mycophenolate Mofetil (MMF) oral; 500mg x 4/d from day +5<br><br>➔ <u>in the control group:</u><br>ATG (Thymoglobuline®) 2.5 mg/Kg/day IV for 2 consecutive days (day -2 and -1)<br>Cyclosporine A (CsA) 3mg/kg/day IV from day -3<br>In case of unrelated donor: Mycophenolate Mofetil (MMF) oral; 500mg x 4/d from day -3<br><br>d- <u>Supportive care</u>: will be performed according to each participating center usual practice
Risks added by the research	*Risk associated with the use of Cyclophosphamide (experimental group):*<br>Cyclophosphamide (CY) is an alkylating agent which prevents cell division primarily by cross- linking DNA strands. CY is converted to its active form in vivo by hepatic enzymes. After a single dose, tissue enzymes degrade most of the active metabolites. After high doses (> 40 mg/kg), the alkylating activity in the plasma is minimal by 24 hours. Several of the metabolites appear to have toxic actions. One of the metabolic products, acrolein (CH2=CH-CHO), is known to be toxic to the bladder urothelium and can cause hemorrhagic cystitis when CY is administered at high doses. Some of the most common toxicities associated with cyclophosphamide include:<br>Gastrointestinal: nausea, vomiting and anorexia<br>Hematologic: myelosuppression<br>Cardiovascular: severe chronic heart failure characterized by cardiomegaly, pericardial effusions, diffuse voltage decrease on ECG and decreased LVEF<br>Genitourinary: hemorrhagic cystitis (prevented by hydration and mesna therapy or bladder irrigation) and gonadal function impairment<br>Miscellaneous: fluid retention, alopecia and rare pulmonary toxicity

	Risk associated with the use of Rabbit Anti-Thymocyte Globulin (control group):<br>The ATG to be used in this trial is a purified preparation of rabbit gamma globulin containing high concentrations of antibodies against human lymphocytes. The preparation may contain low levels of antibody that cross-react with human platelets, white cells or red cells. The potential side effects of ATG are:<br>Hematologic: neutropenia and thrombocytopenia<br>Dermatologic: skin rash and itching<br>Neurologic: fever, chills,<br>Miscellaneous: serum sickness (severe skin rashes, mouth and vaginal sores, pain and swelling of the joints, or kidney damage) and anaphylaxis (hypotension, wheezing, difficulty breathing and severe hives)
Practical procedure	After randomization, patient will be in transplant period between D-6 to D+28/+35 (for experimental and control group): evaluation of early toxicity and initials response.<br><br>Post-transplant period at 3M, 6M, 12M: performance status, evaluation of QOL, toxicity, infections, acute GVHD (and chronic GVHD for 6M and 12M), date of discontinuations of immunosuppressive medication, blood counts, bone marrow aspiration.
Number of subjects chosen	Up to 88 patients will be included to obtain 80 analysable patients (40 per arm), when 80 patients will have received transplant (defined as the infusion of the graft on day 0) inclusions will be stopped
Number of projected centers	12
Research period	The total duration of the study is expected to be 37 months (duration of recruitment: 24 months and study duration for a considered patient: 13 months).
Number of inclusions expected per centre and per month	(see table below)

N°	Town	Country	Expected recruitment/month	Total
1	Paris	France	0.57	15
2	Marseille	France	0,93	14
3	Nantes	France	0,54	13
4	Lille	France	0,39	7
5	Toulouse	France	0,30	7
6	Caen	France	0,13	3
7	Montpellier	France	0,21	5
8	Lyon	France	0,14	3
9	Rouen	France	0.26	5
10	Nancy	France	0.15	2
11	Montpellier	France	0.13	2

<table>
<tr><td></td><td><table><tr><td>12</td><td>Grenoble</td><td>France</td><td>1</td><td>12</td></tr><tr><td colspan="3">TOTAL</td><td>4.75</td><td>88</td></tr></table></td></tr>
<tr><td>Statistical analysis</td><td>The primary analysis of this hypothesis generation study is to estimate the composite endpoint of graft–versus-host disease-free, relapse-free survival (GRFS) at 12 months after allogeneic stem cell transplantation in both treatment arms. In fact, it is more and more established that such composite endpoint acknowledges that both survival and rates of other critical events are important when testing new interventions. Recently, the Blood and Marrow Transplant Clinical Trials Network (BMT CTN) recognized the potential utility of a composite endpoint in trials of allo-SCT. The novel composite endpoint of GVHD-free, relapse-free survival (GRFS) after allo-SCT, defined as grade 3-4 acute GVHD, chronic GVHD requiring systemic treatment, relapse, or death, is a clinically very meaningful one because it represents ideal recovery from allo-SCT (at 1 year) and a measure of cure without ongoing morbidity.
Sample size and power considerations are based on the comparison of PTCy to ATG arms. PTCy will be considered as promising relative to ATG if the HR relative to ATG, after adjustment on covariates, is significant at the one-sided significance level of 0.05. We are using one-sided testing since it is a phase II study to identify if PTCy should be considered as promising for further phase III study. The follow-up will be censored at 1 year for all patients. The probability of GRFS at 1 year in the ATG group was assumed to be 30, 35, 40 or 45%, while probabilities in the PTCy group was assumed to be 15, 20, 25 or 30% higher at 1 year, depending on the scenario. The simulation results are shown in the Table below.
With 80 patients, the study design has 58-60.6% power to identify PTCy as superior to ATG when its GRFS at 1 year is 20% better than ATG, and 75.2-76.7% power to identify PTCy as superior to ATG when its GRFS at 1 year is 25% better than ATG.</td></tr>
<tr><td>Funding source</td><td>PHRC –K 2015 (DGOS-INCA)</td></tr>
<tr><td>Data Safety Monitoring Board anticipated</td><td>Yes</td></tr>
</table>

1 SCIENTIFIC JUSTIFICATION FOR THE RESEARCH

1.1 Hypothesis for the research

- Reduced-intensity conditioning (RIC) regimens

Allogeneic stem cell transplantation (allo-SCT) is a well-established therapy for different hematologic malignancies. However, most hematological malignancies concern older patients (Osca-Gelis et al., 2013; Smith et al., 2015; Smith et al., 2011). Over the years, recognition of the need to offer transplantation to older adults and/or patients with comorbid disease has spurred the development of less toxic, more tolerable preparative regimens – the so-called reduced intensity conditioning (RIC) regimens (Slavin et al., 1998). The hazard of death associated with allo-SCT has decreased significantly over the past decade, with a reduction in non-relapse mortality (NRM) of over 50% along with better long-term survival after allo-SCT, and this is felt to be due in large part to the introduction of RIC regimens. Considering that allogeneic transplants are being increasingly performed in older patients with higher risk disease and more comorbid illnesses, this reduction in non-relapse mortality (NRM) is remarkable (Gooley et al., 2010).
In this setting, the combination of fludarabine and 2 days of busulfan (Flu-Bu2) is a widely used RIC regimen (Malard et al., 2011; Mohty et al., 2003a). Initially described in HLA identical sibling peripheral blood stem cell transplantation, Flu-Bu2 was combined with anti-T-lymphocyte globulin (ATG) (Fresenius 10 mg/kg/day) and cyclosporine A (CsA) alone for the prophylaxis of graft-*versus*-host-disease (GVHD) (Slavin et al., 1998). However, the best GVHD prophylaxis combination in the Flu-Bu2 RIC regimen has not yet been established.
In contrast to allogeneic allo-SCT with myeloablative conditioning, disease control after RIC relies on the development of an immunological graft-*versus*-leukemia effect (GVL) (McSweeney et al., 2001) (Giralt et al., 1997). Since GVHD and GVL are closely linked, the possibility of discriminating between them by the use of *in vivo* T-cell depletion remains challenging, particularly in allogeneic allo-SCT performed after RIC (Baron et al., 2012). In this context, ATG can effectively prevent acute and chronic GVHD, but can also increase the risk of infectious complications and of relapse (Soiffer et al., 2011). It is now established that the effects of ATG in RIC are dose-dependent, and that intermediate doses of thymoglobulin between 4 and 6 mg/kg seem to prevent GVHD optimally while sparing the GVL effect (Baron et al., 2014; Mohty et al., 2003b) (Crocchiolo et al., 2013).
Apart from ATG, the impact of the post-transplant immunosuppression on transplant outcomes in allogeneic HSCT following RIC has been poorly explored. While the combination of CsA and a short course of methotrexate after transplantation is considered as the gold standard for GVHD prophylaxis after conventional myeloablative allogeneic allo-SCT from HLA-identical siblings (Storb et al., 2010), there is no consensus on the optimal preventive regimen for GVHD prophylaxis after RIC allogeneic allo-SCT. In the context of non-ATG-containing, fludarabine-based RIC from HLA-identical sibling donors was recently evaluated by Piñana *et al* (Pinana et al., 2010). The authors found similar post-transplant outcomes (cumulative incidence of acute and chronic GVHD, 2-year relapse and OS) with either a combination of CsA and methotrexate (MTX) or of CsA and mycophenolate mofetyl (MMF). Recently, Rubio et al observed similar outcomes in the group of patients who received MTX or MMF and CsA without ATG, but this group had a higher risk of chronic GVHD leading to worse survivals. In the context of ATG-containing regimens, the addition of MMF or MTX to CsA did not reduce the risk of acute GVHD, but significantly increased the risk of relapse, possibly as a consequence of the relatively reduced risk of chronic GVHD, leading to worse leukemia-free and overall survival (Rubio et al., 2015).
Chronic GVHD was previously reported as a very important process mediating the GVL effect, and with a strong correlation with reduced relapse rates, mainly in the RIC setting, compensating for the lower chemotherapy dose and, thus, the lower anti-leukemic effect (Weisdorf et al., 2012). Also, ATG can induce profound immune suppression and increased incidence of opportunistic infections, especially EBV-related complications (relative risk=4.9, 95% CI,[1.1-21.0]; P=0.03 (Peric et al., 2011).

- Post-transplantation cyclophosphamide (PTCy)
High dose post-transplantation cyclophosphamide (PTCy) was also developed to facilitate HLA-haploidentical allo-SCT using unmanipulated bone marrow (BM) cells as a method of selective *in vivo* allodepletion (Luznik et al., 2012). High-dose PTCy is an attractive approach for crossing the HLA barrier in allogeneic allo-SCT because the treatment is affordable, strikingly effective, and requires no special expertise beyond IV chemotherapy administration. Cyclophosphamide-induced tolerance is an example of the larger phenomenon of drug-induced immunologic tolerance (Schwartz and Dameshek, 1959).

It is now well established that the timing of drug administration is critical to the achievement of tolerance and that cyclophosphamide can only induce transplantation tolerance in a narrow window of time after first antigen exposure (Mayumi et al., 1996; Nomoto et al., 1992). In murine models, PTCy showed to be effective in preventing both acute and chronic GVHD, given its capacity to preferentially eliminate allo-reactive T cells, to preserve donor T regulatory cells (Tregs) and also to promote Tregs expansion, including Tregs in lymphoid organs (Ganguly et al., 2014; Luznik and Fuchs, 2010; Mayumi et al., 1996).
In a clinical trial, Luznik et al. evaluated the safety and efficacy of high-dose PTCy to prevent graft rejection and graft-versus-host disease (GVHD) after outpatient nonmyeloablative conditioning and T cell-replete bone marrow transplantation from partially HLA-mismatched (haploidentical) related donors in 68 patients(Luznik et al., 2008). The cumulative incidences of grades II-IV and grades III-IV aGVHD by day 200 were 34% and 6%, respectively. There was a trend toward a lower risk of extensive cGVHD among recipients of 2 versus 1 dose of PTCy (p = .05). The cumulative incidences of nonrelapse mortality (NRM) and relapse at 1 year were 15% and 51%, respectively. Overall survival (OS) and event-free survival (EFS) at 2 years after transplantation were 36% and 26%, respectively. Recently, 372 consecutive adult hematologic malignancy patients who underwent this procedure were retrospectively studied. Patients received uniform conditioning, T-cell-replete allografting, then PTCy, mycophenolate mofetil, and tacrolimus. Six-month probabilities of NRM and severe aGVHD were 8% and 4%. With 4.1-year median follow-up, 3-year probabilities of relapse, progression-free survival (PFS), and overall survival (OS) were 46%, 40%, and 50%, respectively (McCurdy et al., 2015).
Subsequently, the efficacy of PTCy as sole GVHD prophylaxis after allogeneic bone marrow transplantation using fludarabine and busulfan in myeloablative conditioning regimen was also shown in a multi-institutional study (Kanakry et al., 2014). The cumulative incidences of grades 2 to 4 acute, grades 3 to 4 acute, and chronic GVHD were 51%, 15%, and 14%, respectively. NRM at 100 days and 1 year were 9% and 16%, respectively. With a median follow-up period of 2.2 years, the 2-year disease-free survival and OS rates were 62% and 67%, respectively.
The use of PBSCs instead or BM after T-cell replete haploidentical transplantation did not appear to be detrimental in terms of either GVHD or engraftment rate (Castagna et al., 2014). BM is not the preferred source of stem cells after RIC allo-SCT and the potential efficacy of PTCy on preventing GVHD when using PBSCs is still under debate. This point is of major concern as PBSCs represent the main stem cell source of allogeneic cells worldwide. A recent single centre phase 2 study (n=49) suggested that PTCy alone may not be the preferred GVHD prophylaxis following a RIC transplant with PBSCs (Bradstock et al., 2015). Interpretation of the above non-randomized data is further complicated by heterogeneity. Holtick et al published a phase II trial that included 11 patients with myeloma or lymphoma who underwent conditioning with fludarabine and busulfan followed by T-replete PBSCT and application of 50 mg/kg/d of cyclophosphamide on day+3 and +4 without other concurrent immunosuppression (Holtick et al., 2015). The incidences of grade II-IV and grade III-IV aGvHD were 45% and 27%, respectively, with a NRM of 36% at one and 2 year suggesting the benefit of adding another immunosuppressive treatment in PBSC transplantation setting. These results highlight the need for a controlled randomized trial in a standardized setting.

- Hypothesis

With the above background, our hypothesis is that PTCy will be an effective treatment for GVHD after Flu-Bu-based RIC regimen. To test this hypothesis, we propose to compare the efficacy of PTCy to current standard of care with ATG after a Flu-Bu-based RIC regimen on GVHD prophylaxis. We hypothesize use of PTCy for prevention of GVHD will improve GVHD-free, relapse-free survival, a composite endpoint which measures freedom from ongoing morbidity and represents an ideal outcome measure after allo-SCT.

1.2 Description of knowledge relating to the pathology in question

GVHD prophylaxis remains a challenging task after allo-SCT. The Flu-ivBu combination is a widely used RIC regimen, endorsed by EMA since July 2014. ATG in combination with cyclosporine-A ± mycophenolate mofetil is the backbone for GVHD prophylaxis in this setting. ATG can prevent GVHD with a good efficacy, but at the cost of a higher toxicity and profound immunosuppression, calling for more effective therapies. The advent of PTCy therapy is nowadays on the cutting edge. PTCy controls alloreactivity through elimination of alloreactive T cells while preserving regulatory T cells, both of which impact allogeneic immune reactions. Thus, the potential efficacy (and cost-effectiveness) of PTCy for GVHD prophylaxis may have a major ATG sparing potential.
This randomized trial represents the first prospective controlled trial aiming to test the use of PTCy in the Flu-ivBu RIC setting. The protocol will use a novel endpoint for benchmarking interventions based on a composite primary endpoint of GVHD-free, relapse-free survival which measures freedom from ongoing morbidity and represents an ideal outcome measure after allo-SCT.

1.3 Description of the population to be studied and justification for the choice of participants

Inclusion criteria:

- Patients aged between 18 and 70 years
- Presence of a hematologic malignancy for which an reduced-intensity conditioning allo-SCT is indicated (eligibility criteria for RIC allo-SCT include at least one of the following parameters: (i) patient age older than 50 years; (ii) heavily pre-treated patients who received an autologous hematopoietic SCT (auto-SCT) or with more than 2 lines of chemotherapy before allo-SCT; and (iii) patients with poor performance status because of significant medical comorbidities as described by Sorror et al.
- Karnofsky index ≥ 70% (Appendix 8)
- Availability of a sibling or unrelated stem-cell donor (10/10-HLA matched unrelated donor)
- Efficient contraceptive method during the whole length of the treatment and in accordance with the SmPC of the administered products
- Written informed consent.

- Affiliation to a social security system (recipient or assign)

Non-inclusion criteria:

The presence of one of the following exclusion criteria makes a patient unable to enter the protocol:
- Creatinine clearance less than 30 mL/min
- Bilirubin or amino-transferases above 3X upper normal limit
- Cardiac ejection fraction less than 40%
- Pulmonary impairment with <50% lung carbon monoxide diffusing capacity (DLCO)
- Known hypersensitivity or contraindication to the use of post-transplant Cy and ATG
- Any circumstance that precludes the use of the drugs involved in the protocol
- Pregnancy or breast-feeding women
- Patients allografts

2 OBJECTIVES

2.1 Primary objective

The present randomized trial is primarily designed to compare at 12 months the efficacy of post-transplant cyclophosphamide (PTCy) versus anti-thymocyte globulin (ATG) for GVHD prophylaxis in the setting of Fludarabine-Busulfan reduced-intensity conditioning (RIC) as determined by a composite endpoint of GVHD-free, relapse-free survival (GRFS) allogeneic peripheral blood stem cell transplantation.

2.2 Secondary objectives

• To evaluate the occurrence of grade 2-4 and grade 3-4 severe acute GVHD, according to the Glucksberg criteria revised by Przepiorka et al., within the first 6 months after transplantation.
• To evaluate the occurrence of chronic GVHD as assessed by NIH Consensus Criteria within the first 12 months after transplantation.
• To evaluate non-relapse mortality within the first 12 months after transplantation.
• To evaluate disease-free and overall survival at 12 months after transplantation.
• To evaluate the Quality of Life (QoL) in both treatment arms.

2.3 Objective of any possible ancillary research

• To increase the knowledge on the mechanism of action of PTCy and ATG in GVHD prophylaxis by descriptive immune recovery studies.

The ultimate goal of this Phase IIB study is to assess the feasibility and inform the design of a subsequent phase III study.

3 PLAN FOR THE RESEARCH - DESCRIPTION OF RESEARCH METHODOLOGY

3.1 Concise description of the primary and secondary assessment criteria

3.1.1 Primary assessment criterion

The primary endpoint of the trial will be assessed by the composite endpoint of graft–versus-host disease-free, relapse-free survival (GRFS) at 12 months after allogeneic stem cell transplantation.

3.1.2 Secondary assessment criteria

• Cumulative incidence of grade 2-4 and grade 3-4 severe acute GVHD, according to the Glucksberg criteria revised by Przepiorka et al., within the first 6 months after transplantation.
• Cumulative incidence of chronic GVHD as assessed by NIH Consensus Criteria within the first 12 months after transplantation.
• Cumulative incidence of non-relapse mortality within the first 12 months after transplantation.
• Disease-free and overall survival at 12 months after transplantation.
• Quality of Life (QoL) in both treatment arms at D-7,D30, D90, D180 and D360.
• Descriptive immune recovery studies (lymphocytes –including regulatory cells- and dendritic cells subsets) related to the use of PTCy versus ATG in GVHD prophylaxis.

3.2 Experimental plan

Randomized trial comparing two groups treated by RIC allo-SCT:

- Experimental group: post-transplant cyclophosphamide
- Control group: ATG

Patients will be randomly assigned (1:1) to receive cyclophosphamide or ATG.

3.3 Number of centres participating

This is a national multicenter trial with a projected number of participating centers of ~~11~~12.

3.4 Identification of the subjects

For this research, the subjects will be identified as follows:
Centre No. (3 numerical positions) - Selection order No. of the person in the centre (4 numerical positions) - surname initial - first name initial
This reference is unique and will be retained for the entire research period.

A randomisation number will be assigned by the eCRF during randomisation. (*This number will have the following format: Two letters positions followed by 6 numerical positions.)*

3.5 Randomisation

Patient will be randomized at the Inclusion Visit

Centralized permuted blocked randomization will be prepared by the clinical research Unit (URC-EST). The investigators at each site will obtain the randomized treatment allocation and prescription using Internet (CleanWeb, Telemedecin Technologies, S.A.S).

4 PROCEDURE FOR THE RESEARCH

4.1 Selection period

The selection visit takes place in all eligible patients no later than 30 days before the inclusion visit. The screening period is defined as the time between the selection visit and the inclusion visit. The information note will be given to the patient.
Satisfactory functioning of organ systems has to be documented before inclusion as outlined in the inclusion criteria. This includes physical exam, chest X-ray, ECG, echocardiogram, lung function test, liver function tests, serum creatinine, BUN, serum total protein. In addition, blood cell counts, including a differential of white blood cells and a bone marrow aspiration, are required in case of acute leukemia patient. Adapted evaluation should be adopted for the different group hematological malignancies.

Subjects whose consent is sought	Who informs the subject and collects their consent	When is the subject informed	When is the subject's consent collected
Patient	Investigator	At selection visit	At inclusion visit

4.2 Inclusion / Randomization visit (J-30, J0)

- Ascertainment of inclusion and exclusion criteria by the investigator
- Assessment that organ functions evaluation has not revealed any exclusion criteria
- Confirmation that an HLA matched family donor or 10/10 matched unrelated donor has been identified
- Eligible patients will be offered to enter the study. If they accept, the investigator will collect informed written consent from the patient.

Evaluation before start of conditioning

A routine exam of clinical chemistry values is performed according to local standards.
Beta-HCG will systematically be performed in women in childbearing age.
For examination of chimerism, samples from patient and donor have to be collected and stored.
The evaluation of quality of life (QOL) will be evaluated by 2 questionnaires: EORTC QLQ-C30 (European Organization for Research and Treatment of Cancer Quality of Life Questionnaire-Core 30) and FACT-BMT (Functional Assessment of Cancer Therapy - Bone Marrow Transplant)

- Patients eligible for the study will be included and randomized in either of the following two groups:

- **Experimental group**

Conditioning regimen:
- 30 mg/m2/day IV fludarabine for 5 days (day-6 to day-2)
- 130 mg/m2/day IV busulfan once daily for 2 days (day -4 and -3)

GVHD prophylaxis:
All patients will receive post-transplant 50mg/Kg/day cyclophosphamide IV (day +3 and +4) AND cyclosporine-A IV alone (from day +5) in case of an HLA-sibling donor, or cyclosporine-A IV and mycophenolate-mofetil oral (from day +5) in case of an HLA-matched unrelated donor

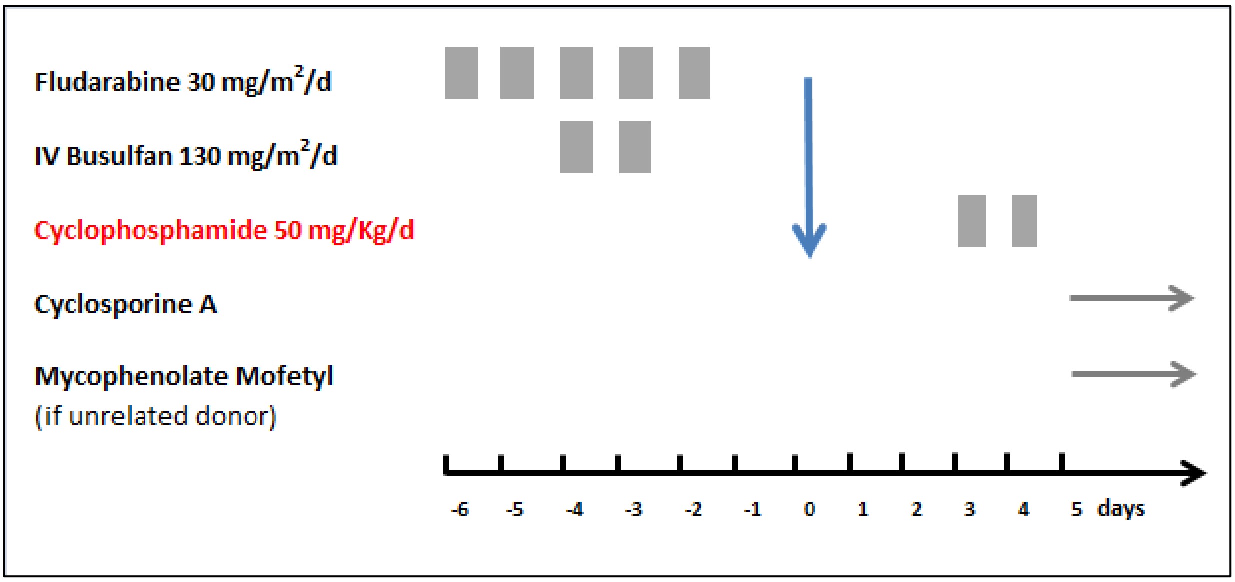

- **Control group**

Conditioning regimen:
- 30 mg/m^2/day IV fludarabine for 5 days (day-6 to day-2)
- 130 mg/m^2/day IV busulfan once daily for 2 days (day -4 and -3)

GVHD prophylaxis:
- 2.5 mg/Kg/day IV, ATG (Thymoglobuline®) for 2 consecutive days (day -2 and -1)
All patients will receive cyclosporine-A IV alone (from day -3) in case of an HLA-sibling donor, or cyclosporine-A IV and mycophenolate-mofetil (MMF) oral (from day -3) in case of an HLA-matched unrelated donor.

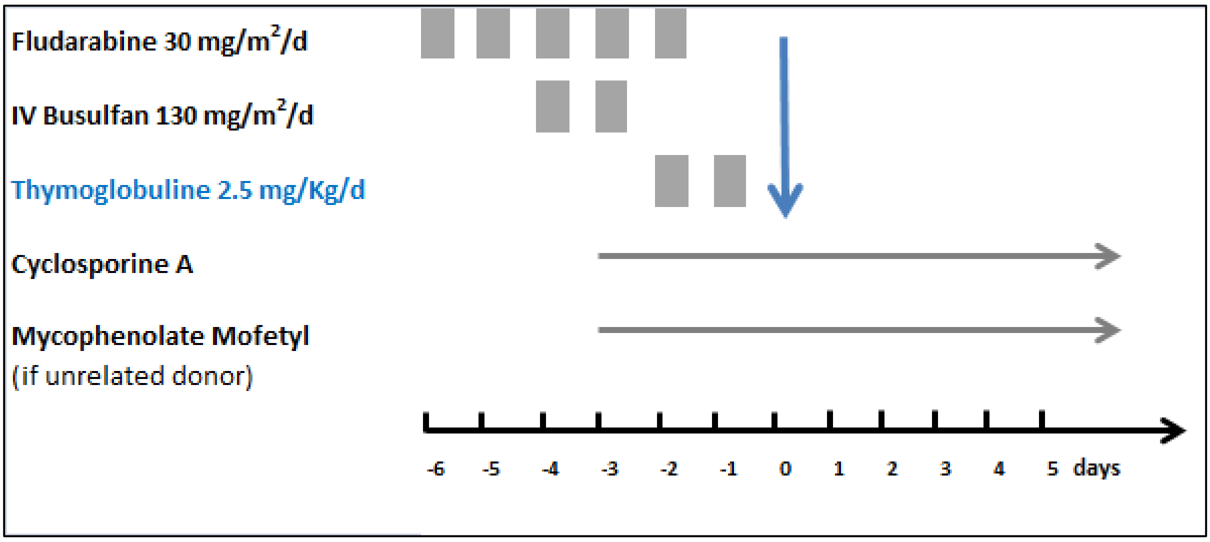

→ G-CSF-mobilized PBSCs are the only stem cell source accepted in the protocol

Donor selection

Only HLA matched family donors and 10/10-HLA matched unrelated donors will be selected for the purpose of this study. After informed consent, HLA matched family donors will undergo a clinical and biological evaluation according to the national recommendations of the «Agence de Biomédecine». Of note, donors should have no contra-indications for the mobilization of peripheral blood stem cells using GCSF

→ Potential family members are initially typed at the HLA-A, -B, -DRB1 and -DQB1 loci at an intermediate resolution level. Donor selection is performed on the basis of a high resolution (4 digit) typing of HLA-A, B, C, DRB1, DQB1. Patients with an HLA identical family donor willing to donate stem cells OR unrelated donor with matching in 10/10 alleles (HLA-A, B, C, DRB1, DQB1) available and ready to donate within 4 weeks from decision to proceed to transplant will be excluded from the study.

Donor mobilization

Donor mobilization with G-CSF: this will be performed according to the national recommendations of the Société Française de Greffe de Moelle et de Thérapie Cellulaire (SFGM-TC) and the “Agence de Biomédecine”. The donor will receive 10 µg/Kg donor body weight of G-CSF, starting on day-5 before the planned first day of apheresis. Mobilized peripheral blood stem cells (PBSC) are the preferred type of graft. A total number of 6.0 x 10^6 CD34+ cells per kilogram recipient's body weight should be targeted with an acceptable minimum of 2.0 x 10^6 CD34+ cells per kilogram recipient's body weight.
Poor stem cell mobilisation or poor marrow harvest will be reported in the CRF. However, since they are not under the influence or responsibility of the transplant physician or the sponsor of the study, they will not be regarded as protocol violations.

Graft Evaluation (tests are performed as part of graft processing)

- Gram stain (done locally) post-selection
- Total nucleated cell count (done locally) pre- and post-processing
- Endotoxin testing post-processing, done locally or sent to an authorized lab
- Flow cytometric analysis for CD34+ cells and for CD3+ cells pre- and post- processing done locally using validated SOPs

Graft infusion

Grafts are transfused without any further manipulation such as T-cell depletion and CD34+ selection are not permitted. Transplantation should be performed within 72 hours from start of first apheresis.
The production of PBSC follows the standard operation procedures of the participating centers or, in case of an unrelated donor, of the donor search centers. It is the responsibility of the producing institution to perform the stem cell harvest according to GMP guidelines and national and international laws.

Transplant Procedures

Unmanipulated PBSC grafts will be administered on Day 0 to all patients according to individual institutional guidelines after appropriate processing and quantification has been performed by the local laboratory. Stem cells are administered through an indwelling central venous catheter. If infusion occurs over two days, Day 0 is the day the last infusion is completed.

4.3 Follow-up Visits

Evaluation of early toxicity and initial response after allo-SCT (day +28 to day +35)

- day of neutrophil (>500/µl) and platelets (first of three days with >20G/l without transfusion) engraftment
- performance status
- evaluation of QOL: EORTC QLQ-C30 and FACT-BMT questionnaires
- maximum toxicity with respect to mucositis, liver, pancreas, kidney, lung, heart, neurological system according to CTC criteria (cf. appendices)
- infections (bacteremia, fungemia, invasive fungal infection, CMV reactivation and disease, other viral reactivation or infection),
- acute GVHD (cf. appendices)
- bone marrow aspiration with evaluation of morphological response as well as chimerism from peripheral blood

Evaluation during follow up at 3 months from allo-SCT (day +90 to day +100)

- performance status
- evaluation of QOL: EORTC QLQ-C30 and FACT-BMT questionnaires
- maximum toxicity with respect to mucositis, liver, kidney, lung, heart, neurological system according to CTC criteria (cf. appendices)
- infections (bacteremia, fungemia, invasive fungal infection, CMV reactivation and disease, other viral reactivation or infection)
- acute GVHD (cf. appendices)
- date of discontinuation of immunosuppressive medication
- blood counts
- bone marrow aspiration with evaluation of morphological response in case of acute leukemia or adapted - evaluation process as well as chimerism from peripheral blood

Evaluation at 6 months ± 15 days after allo-SCT

Evaluations at 6 months remain unchanged.

- performance status
- evaluation of QOL: EORTC QLQ-C30 and FACT-BMT questionnaires
- maximum toxicity with respect to mucositis, liver, pancreas kidney, lung, heart, neurological system according to CTC criteria (cf. appendices)
- infections (bacteremia, fungemia, invasive fungal infection, CMV reactivation and disease, other viral reactivation or infection)
- grade of acute and chronic GVHD (cf. appendices); GVHD is classified according to clinical symptoms
- date of discontinuation of immunosuppressive medication
- blood counts
- bone marrow aspiration with evaluation of morphological response for acute leukemia and adapted evaluation for other malignancies and chimerism in peripheral blood.

4.4 End of research visit

Evaluation at 12 months ± 15 days after allo-SCT

Evaluations at 12 months remain unchanged.

- performance status
- evaluation of QOL: EORTC QLQ-C30 and FACT-BMT questionnaires
- maximum toxicity with respect to mucositis, liver, pancreas kidney, lung, heart, neurological system according to CTC criteria (cf. appendices)
- infections (bacteremia, fungemia, invasive fungal infection, CMV reactivation and disease, other viral reactivation or infection)
- grade of acute and chronic GVHD (cf. appendices); GVHD is classified according to clinical symptoms, irrespective to the time interval to pDLI
- date of discontinuation of immunosuppressive medication
- blood counts
- bone marrow aspiration with evaluation of morphological response for acute leukemia and adapted evaluation for other malignancies and chimerism in peripheral blood.

4.5 Expected length of participation and description of the chronology and duration of the research.

Duration of recruitment: 24 months
Duration of participation for each patient: 13 months
Total duration of the study: 37 months

4.6 Table or diagram summarising the chronology of the research - Distinction between current practice and research

Actions	Screening period	Inclusion visit before start of conditioning	D28 to D35	M3 (D90 to D100)	M6 +/- 15 days	M12 +/- 15 days End of research
Informed consent		R				
History	C	C	C	C	C	C
Clinical exam, heart rate, blood pressure,respiration-SaO2, temperature, height, and weight, performance status (Appendix 7)	C	C	C	C	C	C
ECG	C					
Chest X-ray	C					
Echocardiography	C					
Hematology(1)	C		C	C	C	C
pregnancy test(2)	C	C	C	C	C	C
Bone marrow aspiration	C		C	C	C	C
Serum chemistries	C	C	C		C	C
Serum creatinin measurement, blood urea nitrogen and serum total protein	C		C	C	C	C
Bilirubin, Transaminases, γGT, Phosphatases Alcalines measurement	C		C	C	C	C
Pulmonary function tests (3)	C				C	C
Chimerism (4)		C	C	C	C	C
Quality of life evaluation (5)		R	R	R	R	R
Adverse events			R	R	R	R
Date of neutrophils and platelets engraftment			C			
Infection			C	C	C	C
GVHD			C	C	C	C
Dispensation of experimental medication		R				
Samples for biological collection (6)		R	R	R	R	R

R: Research ; C : Current Practice

(1) A differential of white blood cells and a bone marrow aspiration are required in case of acute leukemia patient. Adapted evaluation should be adopted for the different group haematological malignancies.

(2) A pregnancy test for women of childbearing capacity must be performed at selection visit, before start of conditioning and then at monthly interval during the whole length of the treatment and in accordance with the SmPC of the administered products.

(3) Evaluation should include body plethysmography (RV, TLC), spirometry (FVC, FEV1), Hb-corrected DLCO/TLCO
(4) For examination of chimerism, samples from patient and donor have to be collected and stored.
(5) The evaluation of quality of life (QOL) will be evaluated by 2 questionnaires: EORTC QLQ-C30 (European Organisation for Research and Treatment of Cancer Quality of Life Questionnaire-Core 30) and FACT-BMT

(Functional Assessment of Cancer Therapy - Bone Marrow Transplant)
(6) If accepted by the patient on the informed consent

4.7 Biological Collection

A biological collection will be constituted to perform immune recovery studies related to the use of PTCy versus ATG in GVHD prophylaxis

Post-transplantation cyclophosphamide (PTCy) is an effective prophylaxis against graftversus- host disease (GVHD). Ganguly et al have recently found, in murine model, that PTCy-mediated protection against GVHD is not singularly dependent on depletion of donor alloreactive T cells but also requires rapidly recovering donor Tregs to initiate and maintain alloimmune regulation (Ganguly et al., 2014). However, the cellular mechanisms by which PTCy prevents GVHD remain unclear, particularly concerning other immune cells implicated in GVHD physipathology.

This ancillary study will be dedicated to compare immune reconstitution related to the use of PTCy versus ATG in GVHD prophylaxis. A comprehensive analysis of immune cells will be performed by flow cytometry to identify and characterize immune cells involved in the modulation of host immune responses, in the control of infections and alloreactivity.
For that purpose, the following T, B and dendritic cell subsets will be investigated:

- B cell subsets: naïve, memory, transitionnal and regulatory B cells
- Effector T cell subsets: naïve, memory, Tscm,
- Innate cells: NK cells, invariant NKT cells, mucosal-associated invariant T cells (MAIT), gamma-delta T cells
- Dendritic cells: CD1c+ DC, plasmacytoid dendritic cells, CD141+ DC, Slan-DC, monocytes
- Regiulatory cells: Foxp3 regulatory T cells, MDSC

Phenotypic and functional studies will be performed at different time : inclusion visit, then at day 18-24, day 30, day90, day180 and day 360 for all patients included in this study.
Blood samples will be collected on EDTA (30 mL) and after Ficoll, peripheral blood mononuclear cells (PBMC) will be frozen and stored until use. Serum (5 mL) will also be collected for each patient and frozen for subsequent analyses.

This part of the project will be performed by our research team at the research site Saint Antoine: INSERM UMR938 under the responsibility of Pr Mohty.

4.8 Termination rules

4.8.1 Criteria and methods for prematurely terminating the research treatment

4.8.1.1 Different situations

- Temporary termination of treatment, the investigator must document the reason for stopping and restarting the treatment in the subject's source file and the case report form (CRF)
- Premature termination of treatment, but the subject is still included in the research, until the end of the subject's participation; the investigator must document the reason.
- Premature termination of treatment and end of participation in the research. The investigator must:
    - Document the reason(s)
    - Collect the assessment criteria when participation in the research ends, if the subject agrees
    - Contact the patient by telephone at M12 to collect vital status.

4.8.1.2 Criteria and methods for the premature termination of the research

The Data and Safety Monitoring Board will meet on a regular basis and may decide to stop treatment based on observed adverse effects. Premature discontinuation of the study shall occur in the case there is:

- excessive grade III-IV acute GVHD severe at day 100 after allo-SCT >= 25%
- excessive non-relapse mortality at day 100 after allo-SCT >=25%

- Any subject can withdraw from participating in the research at any time and for any reason.

- The investigator can temporarily or permanently end a subject's participation in the research for any reason that affects the subject's safety or which would be in the subject's best interests.
- If a patient is lost to follow up, action should be made to know the vital status of the patient. The site will need to take action in locating the patient. The coordinator should make at least two attemps to contact the patient by telephone and two additional attempts to contact the patient’s emergency contact if this information is available. If this is not successful, as last resort, the coordinator should check the national death registries where approved by regulatory authorities and available. The check of the death registries will only be permitted if the patient has consented.

If a subject leaves the research prematurely, data relating to the subject can be used unless an objection was recorded when the subject signed the consent form.

If consent is withdrawn, no data about the subject may be used unless the subject states in writing that he/she does not object. In practice, the subject is excluded from the research.

The case report form must list the various reasons for ending participation in the research:
- ☐ Ineffective
- ☐ Adverse reaction
- ☐ Other medical problem
- ☐ Subject's personal reasons
- ☐ Explicit withdrawal of consent

4.8.2 Follow-up of the subjects after the premature termination of treatment

Ending a subject's participation does not affect the normal management of the subject's illness in any way.
If there are serious adverse events, the investigator must notify the sponsor and monitor the subject until theorical M12. If treatment is stopped prematurely due to a serious adverse event, a serious adverse event notification form will be sent by fax (01 44 84 17 99) to the sponsor. The serious adverse event will be monitored until it is resolved.
Patients who withdraw from the study will be contacted by telephone at M3, M6 and M12 to collect their vital status.

4.8.3 Methods for replacing subjects, if applicable

Patients discharged prematurely or excluded from the research will not be replaced. Analysis will be by intention to treat, including failures, subjects lost to follow up or with missing data, patients who died and those who discontinued treatment for intolerance or side effects. The inclusion and treatment numbers of patients discharged prematurely should not be reused.

4.8.4 Terminating part or all of the research

AP-HP as sponsor or the Competent Authority (ANSM) can prematurely terminate all or part of the research, temporarily or permanently, upon the recommendation of a data and safety monitoring board in the following situations:
- first of all, if suspected unexpected serious adverse reactions (SUSARs) are seen in an arm being treated or if there is a discrepancy in the serious adverse reactions between the 2 arms being treated, and which require a reassessment of the benefit-risk ratio for the research.
- likewise, unexpected facts, new information about the product, in light of which the objectives of the research or of the clinical programme are unlikely to be achieved, can lead AP-HP as sponsor or the Competent Authority (ANSM) to prematurely halt the research.
- AP-HP as sponsor reserves the right to permanently suspend inclusions at any time if it appears that the inclusion objectives are not met.

If the research is temporarily prematurely terminate patients already included should continue the treatment. If it is permanently terminate all patients must stop the treatment.

If the research is terminated prematurely, the decision and justification will be given by the sponsor, AP-HP, to the Competent Authority (ANSM) and to the CPP within 15 days, along with recommendations from the Data and Safety Monitoring Board.

5 PATIENTS RECRUITMENT

5.1 Recruitment methods

Up to 88 patients will be included to obtain 80 analysable patients (40 per arm), when 80 patients will have received transplant (defined as the infusion of the graft on day 0) inclusions will be stopped.
Total number of subjects chosen, number of centres, inclusion period and number of subjects/centre are summarized in the following table

Total number of subjects chosen	88
Number of centres	12
Inclusion period (months)	24
Number of subjects/centre	2-15
**Number of subjects/centre/month**	**0.13-0.93**

5.2 Expected number of patients eligible in the centres

N° centers	Speciality	Hospital, Town	Expected recruitement /month	Total/24 months
01	Hematology	Paris	0.57	15
02	Hematology	Marseille	0.93	14
03	Hematology	Nantes	0,54	13
04	Hematology	Lille	0.39	7
05	Hematology	Toulouse	0,30	7
06	Hematology	Caen	0.13	3
07	Hematology	Montpellier	0,21	5
08	Hematology	Lyon	0.14	3
09	Hematology	Rouen	0.26	5
10	Hematology	Nancy	0.15	2
11	Hematology	Montpellier	0.13	2
12	Hematology	Grenoble	1	12

6 TREATMENT ADMINISTERED TO RESEARCH PARTICIPANTS

6.1 Description of the experimental medications

6.1.1 Cyclophosphamide

It is an alkylating agent of the nitrogen mustard type (specifically, the oxazaphosphorine group).

Posology and treatment duration: Cyclophosphamide [50 mg/kg/day] will be given on Day 3 post-transplant (between 60 and 72 hours after marrow infusion) and on Day 4 post-transplant (approximately 24 hours after Day 3 cyclophosphamide).

Preparation: Refer to SmPC

Method of administration: Cyclophosphamide will be given as an IV infusion over 1-2 hours (depending on volume).

Destruction: After every preparation, each empty or opened vial will be destroyed by
each hospital pharmacy according to local procedures relating to cytotoxic agents.

Premedication and supportive care:

Hydration prior to cyclophosphamide may be given according to institutional standards. A recommended approach is as follows: Patients are instructed to increase fluids overnight before cyclophosphamide administration. Hydration with normal saline at 3 ml/kg/h IV will be started 2 hours prior to cyclophosphamide, then the rate will be reduced to 2 ml/kg/h for 1 hour pre- cyclophosphamide and continued at 2 ml/kg/h for 8 hours post-cyclophosphamide.

Mesna (non experimental drug) is required in patients receiving PostCy. Mesna dose must be > 80% of the total daily dose of Cy and given in divided doses 30 minutes before and at 3, 6, and 8 hours after cyclophosphamide intravenously.

Supply:
The intial supply order will be send by the CRA after opening visits.

The hospital pharmacist will confirm receipt in writing of all batches of study medication sent and maintain an accurate accounting of them.

Resupply:
After a patient inclusion/ randomization in the experimental group, the eCRF will send by the document "resupply" (numbers of vials adapted to the patient's weight).

The hospital pharmacist will confirm receipt in writing of all batches of study medication sent and maintain an accurate accounting of them.

Hospital storage:
Drugs must be stored by the pharmacy in accordance with instructions and kept separate from normal hospital drugs.

Preparation:The hospital pharmacy will be in charge of preparation.
The preparation will be made in accordance to SMPC of each commercial drugs, and each bag will be labelled with all mandatory mentions (labels supplied by the sponsor), and its shelf life. For storage conditions and duration after reconstitution please see the SMPC of each drug.
Infusion bags should be stored between 2 and 8°C until administration.

Dispensing:
Pharmacies will dispense to care givers, the experimental medication on the basis of a specific prescription and with respect to local procedures.

Administration: It is important to check the medicinal product labels to ensure that the appropriate preparation is being given to the patient, as prescribed according to the randomization group.

Cyclophosphamide/mesna: Prior, during and immediately after the administration, adequate amounts of fluid should be ingested or infused to force diuresis in order to reduce the risk of urinary tract toxicity. Therefore, Cyclophosphamide should be administered in the morning.

After administration, waste material should be disposed of in accordance with local requirements.
Each administration will be tracked on the “administration patient file”.

Corticosteroids may not be used as an anti-emetic agent and should not be administered until 24 hours after the completion of post-transplantation cyclophosphamide, unless used for adrenal support or during a medical emergency (e.g. treatment of anaphylaxis).

7.1.2 Anti-thymocyte globulin (ATG)

Anti-thymocyte globulin (rabbit) is an immune globulin. It works by suppressing the body's immune response

Supply: According to the Article L 1121-16-1 of the French Public Health Code, institutional sponsor has the option not to provide treatment used in conditions for entitlement to reimbursement by the health insurance. Treatment is prescribed in accordance with the recommandations. So, Anti-thymocyte globulin (ATG) won’t be supplied by the sponsor but by the hospital pharmacies. The specific labeling to regulatory research references will be achieved by each PUI allowed for this optional task (Article R. 5126-9 of the French Public Health Code) accordance with the Article 42 of the LD13 GMP.

Labelling: in accordance with regulatory requirements.

Posology and treatment duration: Rabbit anti-thymocyte globulin will be given at 2.5 mg/Kg/day x 2 days on Days -2 and -1.

Preparation: Refer to SmPC

Method of administration: The dose of ATG will be administered as an IV infusion over 4-8 hours.

Destruction: After every preparation, each empty or opened vial will be destroyed by each care unit (or hospital pharmacy) according to local procedures.

Premedication and supportive care: Methylprednisolone 1 mg/kg will be given as premedication x 2 days with the ATG administration and will be discontinued thereafter. Additional medications to prevent or treat reactions will be administered as indicated according to institutional guidelines. If severe reaction is encountered after the first dose of ATG, the second dose can be delayed until Day +5.

Administration: Each administration will be tracked on the “administration patient file”.

7.1.3 Methods for monitoring compliance with the treatment

Cyclophosphamide mesna and thymoglobulin, will be administered intravenously and will be easily monitored for compliance on “ATG-Cy administration file”.
For corticosteroids, a collection book reporting prednisone tapering will be completed by the patients and transmitted to the investigators at each visit, as well as a copy of prescribed medications.

7.1.4 Accountability and destruction

Unused experimental medications (CYC and mesna) must be accounted by the CRA at the end of the study. After completion, study drug medication (unused) might be destroyed by each hospital pharmacy according to local procedures.

6.2 Description of the non-experimental treatment

Mesna is required in patients receiving PostCy. Mesna dose must be > 80% of the total daily dose of Cy and given in divided doses 30 minutes before and at 3, 6, and 8 hours after cyclophosphamide intravenously.

Fludarabine will be administered intravenously over 30 minutes at a total dose of 150 mg/m^2 divided into 5 daily doses of 30 mg/m^2/day.

Busulfan will be infused once daily intravenously over 3 hours at a dose of 130 mg/m^2/day.

Cyclosporine A CsA is administered at a dose of 3 mg/kg/day by continuous intravenous infusion starting from day +5 in the experimental group and from day - 3 in the control group and doses will be adjusted to maintain a trough level of 200-400 ng/mL. CsA will be changed to twice daily oral dosing as soon as tolerated. CsA is tapered over 4 weeks form day + 62 if clinically possible (GVHD)

Mycophenolate Mofetil
Oral MMF is given at a fixed oral dose of 2 g/day starting from day +5 in the experimental group and from day - 3 in the control group. No treatment adjustment is performed for MMF. MMF is tapered over 4 weeks starting from day + 35 if clinically possible (GVHD).

Methylprednisolone 1 mg/kg will be given as premedication x 2 days with the ATG administration and will be discontinued thereafter. Additional medications to prevent or treat reactions will be administered as indicated according to institutional guidelines. If severe reaction is encountered after the first dose of ATG, the second dose can be delayed until Day +5.

Supportive care

All supportive care will be given in keeping with the local institutional practice. Supportive care should be administered in a similar fashion to subjects randomized to all two arms of the study.

Growth Factors
G-CSF may be given per institutional guidelines.

Seizure Prophylaxis
Rivotril (Clonazepam) will be administered for the prevention of busulfan-associated seizures to all research participants receiving busulfan, starting 12 hours prior to starting busulfan. Dosing of Clonazepam will be administered as per the BMT guidelines. In case of allergic reactions to Clonazepam, alternative anti-seizure medications will be used as clinically indicated

Blood Products
Transfusion thresholds for blood product support will be consistent with standard institutional guidelines. All blood products will be irradiated.

Prophylaxis against Infections
Patients will receive infection prophylaxis according to institutional guidelines. Infection prophylaxis should include, but is not limited to, agents or strategies (e.g., PCR screening and preemptive therapy) to reduce the risk of bacterial, herpes simplex, CMV, EBV, Pneumocystis jiroveci, toxoplasmosis, and fungal infections:

→ Antibiotic prophylaxis for prevention of Pneumocystis pneumonia and infections with encapsulated organisms must be given to all patients until 6 months after discontinuation of all systemic immunosuppressive treatment immune recovery, defined by a normal CD4 T cell count. Suggested antibiotic regimens are: Trimethoprim/Sulfamethoxazole DS 1 tab PO qMWF, or if sulfa allergic, Atovaquone 1500mg PO qMWF or Pentamidine 300 mg inhaled q4 weeks. Additional prophylactic antibiotics are at the discretion of the physician.

→ Cytomegalovirus (CMV): CMV monitoring through nucleic acid amplified testing (NAAT) will be done according to institutional guidelines. It is recommended that weekly assessment for CMV be done through Day 100 and then at each clinical assessment until Day 180 post-transplant or longer if patient still received immunosuppressive therapy. Any reactivation and/or CMV disease will be captured in this study. Pre-emptive treatment (early treatment of CMV viremia detected by PCR) is the preferred strategy for the

majority of patients. It is recommended that the threshold to initiate pre-emptive therapy will be according to a value determined by the assay being performed at the institution or a rising trend on successive measurements from patient's baseline. Populations at risk of CMV infection include all CMV-seropositive patients, all CMV-seronegative patients with CMV-seropositive donors, and all patients receiving blood product transfusions from CMV-seropositive donors.

→ Toxoplasmosis: Toxoplasmosis monitoring through nucleic acid amplified testing (NAAT) will be done according to institutional guidelines, in patients at risk (seropositive patient or donor). Prophylaxis with agents against toxoplasmosis can be given as per local institutional guidelines.

→ Epstein-Barr Virus (EBV): EBV monitoring through nucleic acid amplified testing (NAAT) will be done according to institutional guidelines. It is recommended that EBV monitoring will be done weekly through Day 100 and then at each clinical assessment until Day 180 post-transplant or longer if patient still received immunosuppressive therapy. It is recommended that patients with EBV DNA levels of > 1000 copies/mL receive 375 mg/m^2 of rituximab. Those patients that continue to have levels above 1000 copies/mL on subsequent testing should be considered to receive three additional weekly infusions of 375 mg/m^2 of rituximab. Patients with rapidly rising EBV DNA levels or clinical symptoms are recommended to have imaging studies to diagnose an EBV PTLD. Management of suspected EBV PTLD should be performed according to institution guidelines.

→ Herpes virus (HSV or VZV): Patients must receive acyclovir or valacyclovir through Day 365 post transplant as standard prophylaxis against HSV and VZV per institutional guidelines or until the CD4 T-cell count has normalized.

→ Antifungal prophylaxis: No specific antifungal prophylaxis is recommended. In case it is the routine policy of a centre to give an antifungal prophylaxis in patients with HSCT (i.e., fluconazole, oral amphotericin B, itraconazole, posaconazole or other drugs for which there are published data of controlled trials showing the antifungal prophylaxis of this drug in HSCT patients), **it is required that this policy is being documented in the CRF and the same policy be applied to all the patients included in the centre.**

→ All cases, physicians can refer to the recommendations made by the European Conference on Infections in Leukaemia (ECIL. The full version is available on the website of the EBMT:
https://www.ebmt.org/Contents/Resources/Library/ECIL/Pages/ECIL.aspx

→ treatments given for prevention of infectious complications must be reported in the electronic CRF of the study.

Intravenous Immune Globulin (IVIG)
IVIG administration will be according to local institutional standard practice.

Sinusoidal Obstruction Syndrome (SOS)/Veno-occlusive Disease (VOD) of the Liver Prophylaxis
Prophylaxis against SOS/VOD with heparin and/or ursodiol will be done according to local institutional standard practice.

Donor Lymphocyte Infusions – Viral-specific cytotoxic T-lymphocytes (CTLs)
Donor lymphocyte infusions (DLI) may be performed for the following reason (not restrictive):
- relapsed or persistent disease
- refractory infections
- mixed chimerism (CD3+ donor chimerism <95%)

In all cases, for recommendation regarding DLI indications and doses, physicians can refer to the recommendations made by the SFGM-TC (Guillaume et al., 2014).

Viral specific CTL (donor-derived or third-party) may be given for treatment of infections not responding to standard therapy (e.g. CMV, EBV, adenovirus, etc).

6.3 Description of the traceability elements that accompany the experimental medications

Preparation and administration of experimental medication will be traced.
Trade name with dosage, batch number and expiry date of experimental medications will be reported on a document dedicated to this matter for each patient of the study.

7 ASSESSMENT OF EFFICACY

7.1 Description of parameters for assessing efficacy

7.1.1 Primary Efficacy Outcome Variable

The primary endpoint of the trial will be the assessment of a composite endpoint of graft–versus-host disease-free, relapse-free survival (GRFS) at 12 months after allogeneic stem cell transplantation.

7.1.2 Secondary Efficacy Outcome Variables

The secondary efficacy outcome variables are as follows:

• Cumulative incidence of grade 2-4 and grade 3-4 severe acute GVHD within the first 6 months after transplantation.
• Cumulative incidence of chronic GVHD as assessed by NIH Consensus Criteria within the first 12 months after transplantation.
• Cumulative incidence of non-relapse mortality within the first 12 months after transplantation.
• Disease-free and overall survival at 12 months after transplantation.
• Quality of Life (QoL) in both treatment arms at D-7,D30, D90, D180 and D360.
• Descriptive immune recovery studies (lymphocytes –including regulatory cells- and dendritic cells subsets) related to the use of PTCy versus ATG in GVHD prophylaxis.

7.2 Anticipated methods and timetable for measuring, collecting and analysing the parameters for assessing efficacy

7.2.1 Primary Efficacy Outcome

Parameter: GRFS; Evaluation will be performed at time of inclusion and then at 1, 3, 6 and 12 months post-transplant. For the novel composite endpoint of GVHD-free, relapse-free survival (GRFS) after allo-SCT, events are defined as grade 3-4 acute GVHD, chronic GVHD requiring systemic treatment, relapse, or death. Diagnostic criteria for acute and chronic GVHD are described below.

7.2.2 Secondary Efficacy Outcome

• Parameter: acute GVHD; acute GVHD grading should be performed by the revised Glucksberg criteria (Przepiorka et al., 1995). The time of onset of acute grades II-IV and III-IV acute GVHD will be recorded, as well as the maximum grade achieved. This endpoint will be evaluated through 180 days post HSCT. Investigators should document on a weekly basis (beginning with the day of transplant) the raw data for the GVHD target organs (Appendix 2, 3 and 4). This should include the extent of skin rash, if any; the bilirubin; the daily stool output; or number of stools per day for an outpatient. The weekly record should reflect the worst representative days of the preceding week for each target organ involvement. Biopsy confirmation of target organs is recommended in most circumstances to confirm the diagnosis acute GVHD. In addition, the relevant differential diagnoses should be excluded (e.g., drug rash, gastrointestinal infection such as Clostridium difficile, veno-occlusive disease (VOD), total parenteral nutrition (TPN), etc.).

• Parameter: chronic GVHD; the time of onset of chronic GVHD will be recorded, as well as the requirement for a systemic immunosuppressive therapy and the maximum grade achieved according to the NIH Consensus Criteria (Jagasia et al., 2015) Appendix 5. This endpoint will be evaluated through one-year post HSCT

Diagnosis of chronic GVHD

Minimum one diagnostic manifestation of chronic GvHD OR presence of at least 1 distinctive manifestation plus a pertinent biopsy, laboratory, or other tests (eg, pulmonary function tests [PFT], Schirmer's test), evaluation by a specialist (ophthalmologist, gynecologist), or radiographic imaging showing chronic GvHD in the same or another organ, unless stated otherwise (see Appendix 2 and Appendix 3). Differential diagnosis of chronic GvHD must be excluded e.g. toxicity, infection.

Definition of mild, moderate and severe chronic GvHD

Eight organs or sites (skin, mouth, eyes, gastrointestinal tract, liver, lungs, joint and fascia, and genital tract) are considered for calculating global score. Elements included in the proposed global scoring include both the number of organs or sites involved and the severity score within each affected organ. Performance status scoring is not incorporated into the global scoring system. Organ scoring is performed according to the NIH Working Group consensus (Jagasia et al., 2015) (see Appendix 6).

• Parameter: Cumulative incidence of non-relapse mortality; An event for this endpoint is death without evidence of disease recurrence. Disease recurrence will be considered a competing event. Evaluation will be performed at time of inclusion and then at 1, 3, 6 and 12 months post-transplant.

• Parameter: Disease-free and overall survival; Overall survival is defined as the time interval between date of transplant and death from any cause or for surviving patients, to last follow-up. The event for this endpoint is death from any cause. Relapse-free survival is the time from date of transplant to death or relapse, whichever comes first. The event for this endpoint is relapse or death. Patients alive and free from disease relapse will be censored at last follow-up. Evaluation will be performed at time of inclusion and then at 1, 3, 6 and 12 months post-transplant.

• Parameter: Quality of Life (QoL) in both treatment arms at D-7, D30, D90, D180 and D360. The evaluation of quality of life (QOL) will be evaluated by 2 questionnaires: EORTC QLQ-C30 (European Organization for Research and Treatment of Cancer Quality of Life Questionnaire-Core 30 Appendix 9) and FACT-BMT (Functional Assessment of Cancer Therapy - Bone Marrow Transplant Appendix 10)

• Parameter: Descriptive immune recovery studies (lymphocytes –including regulatory cells- and dendritic cells subsets) related to the use of PTCy versus ATG in GVHD prophylaxis. Phenotypic and functional studies will be performed at day 7-15, day 30, day90, day180 and day 360 for all patients included in this study.

8 SPECIFIC RESEARCH COMMITTEES

8.1 Steering committee

The steering committee includes:

The coordinating investigator: Mohamad Mohty

The missions of the steering committee are to defined objectives of the research, to propose change of the protocol during research, to organize the research, to determine the methodology, to coordinate the informations and to monitor the conduct of research.
The steering committee decide ongoing research what to do in unexpected situation.

The scientific committee meets every 6 months

8.2 Safety Data Monitoring Committee

The Data and Safety Monitoring Board (DSMB) can be established by the sponsor. Its primary mission is to serve as a committee for monitoring safety data. It can have other missions, such as monitoring efficacy data (especially if the protocol includes interim analyses).
The DSMB is mentioned in Article L. 1123-7 of the French Public Health Code.

A DSMB will be convened for this biomedical research -. The DSMB will hold its preliminary meeting before the first inclusion of the first subject. All missions as well as the precise operating methods of the DSMB are described in the charter for the research's DSMB.

General information about the DSMB
The DSMB makes recommendations to the sponsor about the continuation, modification or termination of the research. The recommendations that the DSMB can make are:

- to continue the research with no modification
- to continue the research with a modification to the protocol and/or to the monitoring of subjects
- to temporarily halt inclusions
- to permanently terminate the research in light of:
    - safety data: serious adverse reactions
    - efficacy data: proven futility or efficacy

The DSMB has a consultative role in advising the sponsor on safety issues such as tolerance and re-assessment of the benefit-risk ratio during the research.

Definition of the DSMB's missions:

- Validation of the research methodology:

The proposed methodology for the clinical trial will be validated by the DSMB so that it does not jeopardise the safety of subjects, in particular relating to the inclusion and randomisation methods.

- Validation of tolerance monitoring methods:
    - nature of the evaluated parameters
    - frequency of the evaluations, consultation schedule

- Validation of termination criteria:
    - criteria for terminating a subject's participation for tolerance reasons
    - criteria for the temporary or permanent termination of the research (leading to the establishment of certain recommendations ("stopping rules"))

- Modification of the protocol and recommendations:

In light of the analysis of tolerance data for the research, the DSMB can, when applicable:
propose substantial modifications in order to modify certain data, in particular relating to the protocol (inclusion and non-inclusion criteria, monitoring, additional exams, etc.).Likewise the DSMB can issue any recommendations it deems useful in order to best ensure the safety of the research subjects and to maintain a favourable benefit-risk balance throughout the research.

Definition of the DSMB's operating methods:

- meeting at the first analysis of successful treatments in the 5 first included patients
- reception of SAE notification (grade III and IV, for CTCAE upgrade) from the sponsor to the DSMB

The sponsor retains decision-making authority. When applicable, the sponsor delivers its decision, with justification, and DSMB reports to the Competent Authority (ANSM) and the CPP.

9 SAFETY ASSESSMENT - RISKS AND RESTRICTIONS ADDED BY THE RESEARCH

9.1 Description of parameters for assessing safety

Safety outcome measure (see 10.2)

9.2 Anticipated methods and timetable for measuring, collecting and analysing the parameters for assessing safety (see table 10.2)

1. Parameter: complete blood count with differential counts and coagulation
   Method: Blood count and coagulation
   Timetable:
2. Parameter: renal function, serum chemistry
   Method: potassium, magnesium, creatinine, uric acid, calcium
   Timetable:

3. Parameter: hepatic function
   Method: bilirubin, transaminases, γGT, Phosphatases Alcalines measurement
   Timetable:
4. Parameter: Hemorrhagic cystitis
   Method: clinical history, urine dipstick
   Timtable:
5. Infectious disease
   Methods : clinical history, temperatures, CMV, EBV
   Timetable:
6. Parameter: GVHD assessment
   Method: acute and chronic GVHD grading
   Timetable:

Parameter	Method	Screening period	Inclusion visit before start of conditioning	D28 to D35	M3 (D90 to D100)	M6 +/- 15days	M12 +/- 15 days End of research
**Blood count**	Blood count Coagulation	C	C	C	C	C	C
**Renal function Serum chemistry**	potassium, magnesium, creatinine, uric acide, calcium	C	C	C	C	C	C
**Hepatic function**	Bilirubin, transaminases, γGT, Phosphatases Alcalines measurement	C	C	C	C	C	C
**Hemorrhagic cystitis**	Urine dipstick		C	C			
**Infectious disease**	Clinical history, temperatures, CMV, EBV	C	C	C	C	C	C
**GVHD assessment**	acute and chronic GVHD grading			C	C	C	C

9.3 Procedures in place for recording and reporting adverse events

9.3.1 Definitions

According to Article R1123-39 of the French Public Health Code and the guideline on good pharmacovigilance practices (EMA, 2012) :

- **Adverse event**

Any untoward medical occurrence in a patient or clinical trial subject administered a medicinal product and which does not necessarily have a causal relationship with this treatment.

- **Adverse drug reaction**

Any response to a medicinal product which is noxious and unintended**.**

- **Serious adverse event**

Any untoward medical occurrence that at any dose results in death, is life-threatening, requires inpatient hospitalisation or prolongation of existing hospitalisation, results in persistent or significant disability/incapacity, or is a congenital anomaly/birth defect**.**

- **Unexpected adverse reaction**

An adverse reaction, the nature, severity or outcome of which is not consistent with the applicable product information: the summary of product characteristics (SmPC) for an authorised product or the investigator's brochure for an unauthorised investigational product**.**

According to the notice to sponsors of clinical trials for medications (ANSM):

- **New safety issue**

Any new information regarding safety:
- that could significantly alter the assessment of the benefit-risk ratio for the experimental medication, or for the trial
- or which could lead to the possibility of altering the administration of the experimental medication or altering the conduct of the trial

Examples:
a) any clinically significant increase in the frequency of an expected serious adverse reaction occurring
b) suspected unexpected serious adverse reactions (SUSAR) occurring in patients who have finished the trial and about whom the sponsor is notified by the investigator, who also provides any follow-up reports
c) any new fact relating to the conduct of the clinical trial or the development of the experimental medication, if the new fact is likely to affect participant safety
Examples:
- a serious adverse event likely to be related to the investigations and to the trial's diagnostic procedures and which could modify the conduct of this trial
- a significant risk for the trial participants such as ineffectiveness of the experimental medication used in the trial in treating a life-threatening illness
- significant safety results from a recently completed research carried out on animals (such as a carcinogenicity research)
- the premature termination, or temporary interruption, of a trial conducted with the same experimental medication in another country, for safety reasons
- an unexpected serious adverse reaction associated with a non-experimental medication required for carrying out the trial, (e.g., challenge agents, rescue treatment)

d) recommendations from the data safety monitoring board (DSMB), if applicable, if they are relevant to the safety of the participants
e) any unexpected serious adverse reaction reported to the sponsor by another sponsor of a trial carried out in a different country but relating to the same medication

9.3.2 The investigator's roles

9.3.2.1 Regulatory obligations of the investigator (Art R1123-54 of the French Public Health Code)

The investigator must notify the sponsor, **immediately on the day when the investigator becomes aware**, of all the serious adverse events, except those that are listed in the protocol (see. section 10.3.3.1) or in the investigator's brochure as not requiring immediate notification.

These serious adverse events are recorded in the "adverse event" section of the case report form and the investigator must immediately notify the sponsor's Vigilance division.

9.3.2.2 The investigator's other roles

The investigator must document the serious adverse event as thoroughly as possible and provide the medical diagnosis, if possible.

The seriousness of an AE should not be confused with the severity of the event.
The investigator assesses the severity of the adverse events. The severity of AE is to be graded based on the Common Terminology Criteria for Adverse Events (CTCAE) from National Institutes of Health (Adverse et al.). For events not listed CTCAE, the following guidelines should be used to evaluate the grade of severity for the AE.
Grade I–Mild: Transient or mild discomfort; no limitation in activity; no medical intervention/therapy required
Grade II–Moderate: Mild to moderate limitation in activity; some assistance may be needed; no or minimal medical intervention/ therapy required

Grade III–Severe: Marked limitation in activity; some assistance usually required; medical intervention/ therapyrequired, hospitalization possible
Grade IV–Life Threatening: Extreme limitation in activity; significant assistance required; significant medicalintervention/therapy required, hospitalization

For acute GVH: grading should be performed by the revised Glucksberg criteria (Przepiorka et al., 1995).

For chronic GVHD; the time of onset of chronic GVHD will be recorded, as well as the requirement for a systemic immunosuppressive therapy and the maximum grade achieved according to the NIH Consensus Criteria (Jagasia et al., 2015) Appendix 5.

The method used by the investigator, is based on the WHO method (WHO Uppsala Monitoring Centre) and includes the following four causality terms:

- Certain
- Probable/Likely
- Possible
- Unlikely (not excluded)

Their definition is provided in the table below (from WHO-UMC causality categories, version from 17-Apr-2012).

WHO-UMC causality categories (extract)

Causality term	Assessment criteria*
**Certain**	· Event or laboratory test abnormality, with plausible time relationship to drug intake<br>· Cannot be explained by disease or other drugs<br>· Response to withdrawal plausible (pharmacologically, pathologically)<br>· Event definitive pharmacologically or phenomenologically (i.e. an objective and specific medical disorder or a recognized pharmacological phenomenon)<br>· Rechallenge satisfactory, if necessary
**Probable / Likely**	· Event or laboratory test abnormality, with reasonable time relationship to drug intake<br>· Unlikely to be attributed to disease or other drugs<br>· Response to withdrawal clinically reasonable<br>· Rechallenge not required
**Possible**	· Event or laboratory test abnormality, with reasonable time relationship to drug intake<br>· Could also be explained by disease or other drugs<br>· Information on drug withdrawal may be lacking or unclear
**Unlikely**	· Event or laboratory test abnormality, with a time to drug intake<br>· that makes a relationship improbable (but not impossible)<br>· Disease or other drugs provide plausible explanations

*All points should be reasonably complied with

9.3.3 Specific features of the protocol

All serious and non-serious adverse events must be reported in the CRF.

9.3.3.1 Serious adverse events that do not require the investigator to immediately notify the sponsor

These serious adverse events are only recorded in the "adverse event" section of the case report form.

- **Normal and natural evolution of the pathology :**
  - Hospitalization for the follow-up of the disease under study
- **Complication after transplantation :**
  - Grade < 3 acute GVHD according to the revised Glucksberg criteria
  - Mild or moderate chronic GVHD according to the NIH consensus criteria
  - Infection grade < 3 according to CTCAE scale
  - Aplasia postchemotherapy < 30 days
  - Fever, aplasia, pancytopenia, neutropenia, thrombopenia, anemia < grade 3 according to CTCAE scale.

- **Special circumstances :**
  - Hospitalization for a pre-existing condition
  - Hospitalization for a medical treatment or surgery already planned before the inclusion.
  - Admission for social or administrative reason

- **<u>Adverse events likely to be associated with the treatments prescribed as part of the patient's care during the monitoring of the research</u>**

9.3.3.2 Serious adverse events that require the investigator to immediately notify the sponsor

The investigator must report all adverse events that meet one of the seriousness criteria below, except for events listed in section 10.3.3.1 as not requiring immediate notification to the sponsor :<br>1- Death<br>2- Life threatening situation<br>3- Requiring hospitalisation or prolonging hospitalisation<br>4- Persistent or significant disability or incapacity<br>5- Congenital abnormality or birth defect<br>6- Or any other adverse event considered "medically significant"

9.3.3.3 Other events that require the investigator to immediately notify the sponsor

- **<u>Transplantation complications :</u>**
  - Grade ≥ 3 acute GVHD according to the revised Glucksberg criteria
  - Severe chronic GVHD according to the NIH consensus criteria
  - Infection grade ≥ 3 according to CTCAE scale

- **<u>*In utero* exposure</u>**

The sponsor must be notified immediately about any pregnancy during which the foetus (from the pre-embryonic stage up to birth) could have been exposed at a given time to an experimental medication, even if the pregnancy is not associated with an adverse event.

Notification is required if the exposure involves - the mother or the father

- ***<u>Secondary cancer / myelodysplasic syndroms</u>***

The sponsor must be notified immediately about any secondary cancer or myelodysplasic syndrom.

- ***<u>Serious incident</u>***

Any biovigilance incident must be reported to the local correspondent of biovigilance with the biovigilance form. The sponsor must be informed as soon as the investigator becomes aware of a serious incident.

9.3.4 Procedures and deadlines for notifying the sponsor

Notification of an SAE must initially be provided in a written report using the special form for reporting SAE. The report must be signed by the investigator.

Each item in the form must be completed by the investigator so that the sponsor can carry out the appropriate analysis.

This initial notification must be followed by one or more detailed follow-up report(s), in writing and signed, within a maximum of 8 days in the case of a fatal or life-threatening event and within 15 days for all other cases.

Whenever possible, the investigator will provide the sponsor with any documents that may be useful (medical reports, laboratory test results, results of additional exams, etc.). These documents must be made anonymous. In addition, the documents must include the following: research acronym, number and initials of the subject, nature and date of the serious adverse event.

Any adverse event will be monitored until fully resolved (stabilisation at a level considered acceptable by the investigator, or return to the previous state) even if the subject has left the trial.

The initial notification, the SAE follow-up reports and all other documents must be sent to the sponsor via fax only to the Vigilance Division of the DRCD, fax No. **01 44 84 17 99**

For studies using e-CRF:
- the investigator completes the SAE notification form in the e-CRF, validates, prints and signs the form before sending it *via* fax.
- if it is not possible to connect to the e-CRF, the investigator will complete, sign and send the SAE notification form. As soon as the connection is restored, the SAE notification form in the e-CRF must be duly completed.

The investigator must comply with all requests from the sponsor for additional information.
For all questions relating to the notification of an adverse event, the Vigilance Division of the DRCD can be contacted via email: vigilance.drc@aphp.fr

***In utero* exposure**

The investigator must monitor the pregnant woman throughout her pregnancy or until the pregnancy is terminated, and must notify the sponsor of the outcome of the pregnancy, using this form.
If the outcome of the pregnancy falls within the definition of a serious adverse event (miscarriage, pregnancy termination, foetal death, congenital abnormality, etc.), the investigator must follow the procedure for reporting SAE.
If the exposure involves the father, the investigator must obtain the mother's permission before collecting information about the pregnancy.

The initial pregnancy notification, the SAE follow-up reports and all other documents must be sent to the sponsor via fax only to the Vigilance Division - of the DRCD, fax No. **01 44 84 17 99**.

9.3.5 Period for notifying the sponsor

The investigator must report all SAE that occur in research subjects:
- after the date on which the consent is signed.
- throughout the period during which the participant is monitored, as determined by the research
- with no time limit, if the SAE is likely to be due to the experimental medication or to the research procedures (for example, serious reactions that could appear long after exposure to the medication, such as cancers or congenital abnormalities)

9.3.6 The sponsor's roles

The sponsor, represented by its Vigilance Division, continuously assesses the safety of each experimental medication throughout the research.

9.3.6.1 Analysis and declaration of serious adverse event

The sponsor assesses:
- the seriousness of all adverse events reported
- the causal relationship of these events with each experimental medication and/or specific medical procedures/exams added by the research and with other possible treatments
- the expected or unexpected nature of these adverse reactions

All serious adverse events which the investigator and/or the sponsor believe could reasonably have a causal relationship with the experimental medication are considered as suspected adverse reactions.

All suspected unexpected serious adverse reactions (SUSAR) and serious incident are declared by the sponsor, within the legal time frame, to the Agence Française de Sécurité Sanitaire des Produits de Santé (ANSM, French Health Products Safety Agency) and to the relevant Comité de Protection des Personnes (CPP, ethical committee).

- The initial declaration must be made no later than 7 calendar days after the date on which the serious adverse event occurs in the case of death or of a life-threatening diagnosis.

- The initial declaration must be made no later than 15 calendar days after the date on which the serious adverse event occurs in the case of other serious situations.

- The follow-up declaration must be made no later than 8 days after the 7- or 15-day deadline (depending on the seriousness).

Any suspected unexpected serious adverse reaction must also be declared electronically in the Eudravigilance European database for adverse events due to medications, established by the European Medicines Agency (EMA).

The sponsor must notify all relevant investigators about any data that could adversely affect the safety of the research subjects.

9.3.6.2 Reference safety information

The assessment of expectedness of a serious adverse reaction by the sponsor is realized on the basis of the following reference safety information:

- **Expected serious adverse events related to the experimental drug are :**

Cyclophosphamide
Cyclophosphamide (CY) is an alkylating agent which prevents cell division primarily by cross- linking DNA strands. CY is converted to its active form in vivo by hepatic enzymes. After a single dose, tissue enzymes degrade most of the active metabolites. After high doses (> 40 mg/kg), the alkylating activity in the plasma is minimal by 24 hours. Several of the metabolites appear to have toxic actions. One of the metabolic products, acrolein (CH2=CH-CHO), is known to be toxic to the bladder urothelium and can cause hemorrhagic cystitis when CY is administered at high doses.

Some of the most common toxicities associated with cyclophosphamide include:

- Gastrointestinal: nausea, vomiting and anorexia
- Hematologic: myelosuppression
- Cardiovascular: severe chronic heart failure characterized by cardiomegaly, pericardial effusions, diffuse voltage decrease on ECG and decreased LVEF
- Genitourinary: hemorrhagic cystitis (prevented by hydration and mesna therapy or bladder irrigation) and gonadal function impairment
- Miscellaneous: fluid retention, alopecia and rare pulmonary toxicity

Refer to Endoxan SmPC®

*Rabbit Anti-Thymocyte Globulin (*Thymoglobuline®)
The ATG to be used in this trial is a purified preparation of rabbit gamma globulin containing high concentrations of antibodies against human lymphocytes. The preparation may contain low levels of antibody that cross-react with human platelets, white cells or red cells. The potential side effects of ATG are:

- Hematologic: neutropenia and thrombocytopenia
- Dermatologic: skin rash and itching
- Neurologic: fever, chills,
- Miscellaneous: serum sickness (severe skin rashes, mouth and vaginal sores, pain and swelling of the joints, or kidney damage) and anaphylaxis (hypotension, wheezing, difficulty breathing and severe hives)

Expected serious adverse events related to research procedures (graft complication) are :

Graft Infusion
Symptoms may include changes in heart rate and/or rhythm, changes in blood pressure, fever, chills, sweats, nausea, vomiting, diarrhea, abdominal cramping, hemoglobinuria, acute renal failure, allergic reactions, respiratory dysfunction, or headache.

Infections

Transplantation puts the patient at higher risk for bacterial, viral, or fungal infections, which are potentially

life-threatening. These risks are potentially higher with CD-34 selected transplants. Prophylaxis will be initiated and patients will be closely monitored for signs of infections and will receive early and appropriate treatment.

Graft-versus-host Disease
Acute or chronic GVHD may develop after allogeneic transplantation that can be disabling and can lead to death. GVHD is thought to be initiated by T-cells contained in the graft. CD34+ selection and PTCy reduce the number of alloreactive T-cells but GVHD can occur after these transplants.

Sinusoidal Obstruction Syndrome (SOS)/Veno-occlusive Disease (VOD) of the Liver
SOS/VOD is a manifestation of damage to the liver by the conditioning regimen that usually develops after allogeneic transplant. SOS/VOD diagnosis and severity grading muqt be performed according to the EBMT revised diagnosis and severity criteria(Mohty et al., 2016).

Recipients developing SOS/VOD will be monitored closely and will receive appropriate supportive care and careful fluid management.

End Organ Damage
End organ damage of all or any of the major organs, including the brain, may occur as a result of cumulative toxicity from anti-neoplastic therapy, reactions to other drugs, and as a result of destructive processes (e.g., infection, GVHD, etc.) and may have a fatal outcome. Toxicities may occur in any individual patient due to multiple events and cumulative effects that may involve any and all organs, including the brain. Brain damage can result in severe loss of cognitive or neurologic function. Data from previous studies do not suggest that the risk of end organ damage is appreciably affected by cyclophosphamide or the RIC regimen to be used in this study.

Death
There is an approximate 5-10% risk of transplant-related mortality within the first month of transplant due to the risk of severe regimen related toxicity, hemorrhage, opportunistic infection, or other complications. It is not expected that the regimens to be used in this protocol will increase this risk.

Any serious adverse reaction with fatal issue will be considered as a SUSAR by the sponsor and will be expedited to the competent authorities.

Fludarabine
Fludarabine is a fluorinated nucleoside analog. After phosphorylation to fluoro-ara-ATP the drug appears to incorporate into DNA and inhibit DNA polymerase alpha, ribonucleotide reductase and DNA primase, thus inhibiting DNA synthesis. Excretion of fludarabine is impaired in patients with impaired renal function.

Common side effects of fludarabine include:

- Hematologic: hematopoietic suppression including neutropenia with increased risk of infection and immunosuppression
- Neurologic: peripheral neuropathy and encephalopathy manifested by fatigue, weakness, paresthesia, visual disturbances, somnolence and coma
- Gastrointestinal: nausea, vomiting, diarrhea and stomatitis
- Miscellaneous: fever, skin rash, cough and idiopathic pneumonitis

Refer to Fludara® SmPC

Busulfan
Busulfan (1, 4-dimethanesulfonoxybutane) is an alkylating agent. The drug is extensively metabolized and its metabolites are eventually excreted in the urine. The oral preparation is well absorbed but studies at this institution have indicated that there is a ten-fold variability area under the curve (AUC) of the drug among patients receiving busulfan by mouth. There is a statistical association between increased AUC and the development of veno occlusive disease of the liver. Since its FDA approval in 1999, IV Bu has been used increasingly in combination with CY or Flu. IV Bu was initially administered every 6-hours, similar to oral Bu. More recently, several studies have demonstrated that the use of once-daily intravenous (iv) busulfan instead of oral busulfan was equally effective, with a predictable pharmacokinetic profile and a reduced incidence of severe toxicities (eg, sinusoidal obstruction syndrome)(Almog et al., 2011; Mamlouk et al., 2005).

Toxicities associated with busulfan administration include:

- Gastrointestinal: nausea, vomiting, constipation, diarrhea, abdominal discomfort, anorexia, dyspepsia and mucositis
- Hepatobiliary: veno-occlusive disease
- Neurologic: headache, insomnia and seizures
- Cardiovascular: hypertension, hypotension and tachycardia
- Pulmonary: dyspnea, lung fibrosis
- Endocrine and metabolic: hypermagnesemia, hyperglycemia and hyperphosphatemia
- Miscellaneous: rhinorrhea, amenorrhea, infertility, skin rashes, cataracts

Refer to Busilvex® SmPC

Cyclosporine
The most common side effects of cyclosporine are:

- Cardiovascular: hypertension
- Neurologic: paresthesias, neuropathy and seizures
- Hematologic: thrombotic microangiopathy
- Endocrine and metabolic: electrolyte imbalances
- Miscellaneous: hirsutism; hepatic and renal dysfunction; nephrotoxicity; gingival hyperplasia; transient blindness

Refer to Neoral® SmPC

Mycophenolate Mofetil
The most common side effects of Mycophenolate Mofetil are:

- Neurologic: headache, tremors, insomnia, dizziness, excessive fatigue, weakness
- Cardiovascular: tachycardia
- Pulmonary: dyspnea
- Gastrointestinal: nausea, vomiting, dyspepsia, abdominal pain, diarrhea, hematemesis and hematochezia
- Hematologic: Neutropenia, thrombocytopenia, unusual bruising, and anemia
- Endocrine and metabolic: hyperlipidemia
- Miscellaneous: rash, edema, change in vision, infection, second cancers, teratogenicity, miscarriage, limited effectiveness of birth control, and progressive multifocal leukoencephalopathy (PML).

Refer to Cellcept® SmPC

MESNA (sodium -2-mercapto ethane sulphonate)
Mesna is a prophylactic agent used to prevent hemorrhagic cystitis induced by the oxazophosphorines (cyclophosphamide and ifosfamide). It has no intrinsic cytotoxicity and no antagonistic effects on chemotherapy. Mesna binds with acrolein, the urotoxic metabolite produced by the oxazophosphorines, to produce a non-toxic thioether and slows the rate of acrolein formation by combining with 4-hydroxy metabolites of oxazophosphorines. At the doses used for uroprotection, mesna is virtually non-toxic.

The most common side effects of MESNA are:

- Cardiovascular: hypotension
- Dermatologic: rash, urticarial
- Gastrointestinal: nausea and vomiting, diarrhea, abdominal pain, altered taste
- Neurologic: headache, joint or limb pain
- Miscellaneous: fatigue

Methylprednisolone: refer to Solumedrol® SmPC

9.3.6.3 Analysis and declaration of other safety data

This relates to any safety data or new fact that could significantly alter the assessment of the benefit-risk ratio for the experimental medication, or for the research, or which could lead to the possibility of altering the administration of the experimental medication or altering the conduct of the research.

New facts must be declared to the competent authorities as soon as the sponsor becoming aware. Additional relevant information must be sent within an additional 8 days after the reception of new relevant information.

9.3.6.4 Annual safety report

Once a year for the duration of the clinical trial, the sponsor must draw up an annual safety report (Development Safety Update Report - DSUR) which includes, in particular:
- an analysis of the safety of the research subjects
- a description of the patients included in the trial (demographic characteristics, etc.)
- a line listing of suspected serious adverse reactions that occurred during the period covered by the report
- a cumulative summary tabulation of serious adverse events that have occurred since the start of the research

The report must be delivered no later than 60 days after the anniversary of the date on which the ANSM authorised the trial.

9.3.6.5 Data Safety Monitoring Board

The Data and Safety Monitoring Board (DSMB) can be established by the sponsor. Its primary mission is to serve as a committee for monitoring safety data. It can have other missions, such as monitoring efficacy data (especially if the protocol includes interim analyses).
The DSMB is mentioned in Article L. 1123-7 of the French Public Health Code.

The sponsor is responsible for justifying the creation or absence of a supervisory committee to the Competent Authority (ANSM) and to the CPP.

10 DATA MANAGEMENT

10.1 Data collection methods

Data will be collected in an electronic case report form (e-CRF), devised by the study coordinator in collaboration with URC-EST.

10.2 Identification of data collected directly in the CRFs and that will be considered as source data.

Not applicable for this study: There will be no data collected directly in the CRFs.

10.3 Right to access source data and documents

10.3.1 Access to data

In accordance with GCPs:
- the sponsor is responsible for obtaining the permission of all parties involved in the research to guarantee direct access to all locations where the research will be carried out, to the source data, to the source documents and the reports, with the goal of quality control and audit by the sponsor
- the investigators will make available to those in charge of monitoring, quality control and audit relating to the biomedical research the documents and personal data strictly necessary for these controls, in accordance with the legislative and regulatory provisions in force (Articles L.1121-3 and R.5121-13 of the French Public Health Code)

10.3.2 Source documents

Source documents are defined as any original document or object that can prove the existence or accuracy of a piece of information or a fact recorded during the research. These documents will be kept for 15 years by the investigator or by the hospital in the case of a hospital medical file.

Source documents that will be kept for 15 years are completed and signed CRFs.

10.3.3 Data confidentiality

Those responsible for biomedical research quality control (Article L.1121-3 of the French Public Health Code) will take all necessary precautions to ensure the confidentiality of information about the experimental medications, the research, the research subjects and in particular the identity of the subjects and the results obtained.
These individuals, as well as the investigators themselves, are subject to professional secrecy (in accordance with the conditions set out in Articles 226-13 and 226-14 of the Penal Code).
During or after the biomedical research, the data collected about the research subjects and sent to the sponsor by the investigators (or any other specialised parties) will be made non-identifying.
Under no circumstances should the names and addresses of the subjects involved be shown. Only surname initial and first name initial will be registred with code as it is defined in section 3.3.

The sponsor will ensure that each research subject has given permission in writing for access to personal information about him or her which is strictly necessary for the quality control of the research.

10.4Data processing and storage of documents and data

10.4.1 Identification of the manager and the location(s) for data processing

Data management (including e-CRF design - CleanWEB, Telemedicine S.A.) will be performed by URC-Est and statistical analysis by Dr Labopin. The data base will be transmitted to Dr Labopin after

10.4.2 Data entry

Data entry will be carried out on electronic media via a web browser with a restricted access to investigators.
Data will be completed by the investigators for each visit of follow up with the help of a Clinical Research Technician (CRT) of URC-Est for AP-HP centers and of each center for others centers.

10.4.3 Data processing (CNIL, the French Data Protection Authority) in France

This research falls under the "Méthodologie de référence" (MR-001) according to the provisions of Article 54, paragraph 5 of modified Law No. 78-17 of 6 January 1978 relating to information technology, data files and privacy. This change was approved in a decision made on 5 January 2006. AP-HP, the research sponsor, has signed a commitment to comply with this " Méthodologie de référence "
The processing of personal data for this research falls under the scope of the provisions of Articles 53 to 61 of the Law of 6 January 1978 relating to information technology, data files and privacy, modified by Law No. 0204-801 of 6 August 2004.

10.4.4 Archival

Specific documents for biomedical research relating to a medication for human use will be archived by the investigator and the sponsor for a period of 15 years after the end of the research.

This indexed archival includes, in particular:

- A sealed envelope containing the original copies of all information sheets and consent forms signed for all individuals at the centre that participated in the research for the investigator
- A copy of all the information notes and consent forms signed for all subjects at the centre that participated in the research for the sponsor
- "Research" binders for the Investigator and the sponsor, including:
    - the successive versions of the protocol (identified by the version no. and date), and the appendices
    - the ANSM authorisations and CPP favourable opinions
    - letters of correspondence
    - the inclusion list or register
    - the appendices specific to the research
    - the final research report
- The data collection documents

10.5 Ownership of the data

AP-HP is the owner of the data, which cannot be used or disclosed to a third party without its prior approval.

11 STATISTICAL ASPECTS

11.1 Description of statistical methods to be used including the timetable for the planned interim analyses

Baseline characteristics of patients will be described per group.
Qualitative data will be described with frequencies and percentages; quantitative data will be described with mean and standard error or with median, interquartile interval and range. For categorical variables groups will be compared using the chi-squared test. For continuous data, groups will be compared using the Mann-Whitney test.
The factor “center” will not be considered in statistical models.

Principal criteria analysis:

The primary analysis of this hypothesis generation study is to estimate the composite endpoint of graft–versus-host disease-free, relapse-free survival (GRFS) at 12 months after allogeneic stem cell transplantation in both treatment arms. In fact, it is more and more established that such composite endpoint acknowledges that both survival and rates of other critical events are important when testing new interventions. Recently, the Blood and Marrow Transplant Clinical Trials Network (BMT CTN) recognized the potential utility of a composite endpoint in trials of allo-SCT. The novel composite endpoint of GVHD-free, relapse-free survival (GRFS) after allo-SCT, defined as grade 3-4 acute GVHD, chronic GVHD requiring systemic treatment, relapse, or death, is a clinically very meaningful one because it represents ideal recovery from allo-SCT (at 1 year) and a measure of cure without ongoing morbidity. The GRFS will be calculated using the Kaplan-Meier method, and subgroups will be compared using the log-rank test.

Secondary criteria analysis:

Secondary end points were acute and chronic GVHD, non-relapse mortality (NRM), disease-free survival (DFS), overall survival (OS) and quality of life.

Acute and chronic GVHDs will be graded according to previously published criteria. NRM was defined as death without evidence of relapse or progression. DFS was defined as survival with no evidence of relapse or progression. OS was defined as the time from alloHSCT to death, regardless of the cause. Cumulative incidence functions (CIFs) will be used to estimate NRM in a competing-risks setting, with relapse as competing risk. Acute and chronic GVHD will also be estimated using CIF, we will considered death to be the competing event. Probabilities of DFS and OS will be calculated using the Kaplan–Meier estimates. Univariate analyses will be performed using Gray’s test for CIF and the log-rank test for DFS and OS.

The evaluation of quality of life (QOL) will be evaluated by 2 questionnaires at D-7,D30, D90, D180 and D360: EORTC QLQ-C30 (European Organisation for Research and Treatment of Cancer Quality of Life Questionnaire-Core 30) and FACT-BMT (Functional Assessment of Cancer Therapy - Bone Marrow Transplant)

11.2 Calculation hypotheses for the number of subjects required and the result

Sample size and power considerations are based on the comparison of PTCy to ATG arms. PTCy will be considered as promising relative to ATG if the HR relative to ATG, after adjustment on covariates, is significant at the one-sided significance level of 0.05. We are using one-sided testing since it is a phase II study to identify if PTCy should be considered as promising for further phase III study. The follow-up will be censored at 1 year for all patients. The probability of GRFS at 1 year in the ATG group was assumed to be 30, 35, 40 or 45%, while probabilities in the PTCy group was assumed to be 15, 20, 25 or 30% higher at 1 year, depending on the scenario. The simulation results are shown in the Table below.

With **80 patients,** the study design has 58-60.6% power to identify PTCy as superior to ATG when its GRFS at 1 year is 20% better than ATG, and 75.2-76.7% power to identify PTCy as superior to ATG when its GRFS at 1 year is 25% better than ATG.

GRFS at 1 year		***Power***
**ATG group**	**PT-Cy group**	**N=80 (40 per arm)**
30%	50%	60.6%
	55%	76.7%
35%	55%	58.8%
	60%	75.5%
40%	60%	58%
	65%	75.2%
45%	65%	58.2%
	70%	76%

11.3 Specify if subjects who leave the research prematurely will be replaced and in what proportion.
No replacement is planned for patients leaving research prematurely.

11.4 Anticipated level of statistical significance
All tests will be two-sided, and a P value of <0.05 will be considered significant.

11.5 Statistical criteria for termination of the research.
Not applicable. No interim analysis data planned.

11.6 Method for taking into account missing, unused or invalid data
In case of missing data, patients will be censored at last available follow-up.
To test analyses robustness, analyses under bias maximum hypothesis will be realized.

11.7 Management of modifications made to the analysis plan for the initial strategy.
All modification made to the analysis plan for the initial strategy will be documented in the analysis report.

11.8 Selection of populations
All efficacy analysis will be conducted based on ITT population (all randomized patients). Secondary efficacy analyses will also be conducted based on the “modified intent to treat” (MITT) population defined as patients who received at least one dose of treatement and “per-protocol" population. The “per protocol“ population is defined as all patients randomized and treated without major protocol violations/deviations. Pre-defined major protocol violations/deviations are:

- Non-respect of all selection criteria
- Non-respect of the randomized treatment allocation and/or duration (wrong treatment received, premature discontinuation of treatment – except for death)
- Missing data for the primary efficacy endpoints
- Inclusion in another interventional study
- Major protocol deviation identified during a blinded data review before data base freezing.

Safety analysis will be conducted based on MITT population.

12 QUALITY CONTROL AND ASSURANCE

Each biomedical research project managed by AP-HP is ranked from A to D according to the projected risk incurred by research subjects using the classification of biomedical research sponsored by AP-HP.

12.1 General organisation
The sponsor must be responsible for the safety and respect of those subjects who have agreed to participate in the research. The sponsor must implement a quality assurance system to best monitor the conduct of the research in the investigation centres.

For this purpose, the sponsor shall delegate Clinical Research Associates (CRA) whose primary role is to carry out regular follow-up visits at the research locations, after having carried out initial visits.
The objectives of monitoring the research, as defined in the French Good Clinical Practices (BPC section 5.18.1), are to verify that:

- the rights, safety and protection of the research subjects are met
- the data reported is exact, complete and consistent with the source documents
- the research is carried out in accordance with the protocol in force, with the French GCPs and with the legislative and regulatory provisions in force

12.1.1 Strategy for opening the centres

The strategy for opening the centres established for this research is determined using the appropriate monitoring plan.

12.1.2 Level of centre monitoring

In the case of this research, which is considered **C** risk, the appropriate monitoring level has been determined based on the complexity, the impact and the budget for the research. Thus, the sponsor and the coordinating investigator have agreed on the logistic score and impact, resulting in a research monitoring level to be implemented: level **C**.

12.2 Quality control

A Clinical Research Associate (CRA) appointed by the sponsor will be responsible for the proper conduct of the research, for collecting and documenting, recording and reporting the data generated in writing, in accordance with the Standard Operating Procedures applied within the DRCD and in accordance with the French Good Clinical Practices as well as with the legislative and regulatory provisions in force.

The investigator and the members of the investigator's team agree to make themselves available during Quality Control visits carried out at regular intervals by the Clinical Research Associate. During these visits, the following elements will be reviewed:

- written consent
- compliance with the research protocol and with the procedures defined therein
- quality of the data collected in the case report form: accuracy, missing data, consistency of the data with the "source" documents (medical files, appointment books, original copies of laboratory results, etc.)
- management of the treatments used

12.3 Case Report Form

All information required according to the protocol must be entered in the case report forms. The data must be collected as and when they are obtained, and clearly recorded in these case report forms. Each missing data item must be coded.
This digital case report form will be implemented in each of the centres thanks to a web-based data collection medium. Investigators will be given a document offering guidance in using this tool.
When the investigators complete the case report via the Internet, the CRA can view the data quickly and remotely. The investigator is responsible for the accuracy, quality and relevance of all the data entered. In addition, the data are immediately verified as they are entered, thanks to consistency checks. Thus, the investigator must validate any changes to the values in the case report form. These modifications will be subject to an audit trail. A justification can be added when applicable, as a comment. A print-out, authenticated (signed and dated) by the investigator, will be requested at the end of the research. The investigator must archive a copy of the authenticated document that was delivered to the sponsor.

12.4 Management of non-compliances

Any events that occur as a result of non-compliance, by the investigator or any other individual involved in conducting the research, with the protocol, with the standard operating procedures, with the good clinical practices or with the legislative and regulatory provisions in force must be noted in a declaration of non-compliance addressed to the sponsor. As a first step, major or critical non-compliances will be reviewed and processed by the DRCD's medical coordinator in order to implement the necessary corrective or preventive actions. Next, the non-compliances will be sent to the Quality - Risk Management Division of the DRCD for verification and analysis. These verifications could result in the investigator in charge of the research location in question being asked for information or could lead to compliance or audit visits.

12.5 Audits/inspections

The investigators agree to accept the quality assurance audits carried out by the sponsor as well as the inspections carried out by the competent authorities. All data, documents and reports may be subject to regulatory audits and inspections. Medical secrecy cannot be invoked in opposition to these audits and inspections.

An audit can be carried out at any time by individuals appointed by the sponsor and who are not associated with the research directors. The objective of the audit is to ensure the quality of the research, the validity of the results and compliance with the legislation and regulations in force.

The individuals who lead and monitor the research agree to comply with the sponsor's requirements and with the competent authority regarding research audits or inspections.

The audit may be applicable to all stages of the research, from the development of the protocol to the publication of the results and the organisation of the data used or produced as part of the research.

12.6 Primary investigator's commitment to assume responsibility

Before starting the research, each investigator will give the sponsor’s representative a copy of his/her personal curriculum vitæ, signed and dated, with his/her number in the RPPS (Répertoire Partagé des Professionnels de Santé, Collective Database of Health Professionals).

Each investigator will undertake to comply with the legislation and to carry out the research according to French GCP, adhering to the Declaration of Helsinki terms in force.

The primary investigator at each participating centre will sign a responsibility commitment (standard DRCD document) which will be sent to the sponsor's representative.
The investigators and their employees will sign a delegation of duties form specifying each person's role.

13 ETHICAL AND LEGAL CONSIDERATIONS

13.1 Responsibilities of Investigators

The investigator will be responsible for insuring that the clinical study is performed in accordance with the protocol, the ethical principle that have their origin in the Declaration of Helsinki as well as the Good Clinical Practice. To do this, a copy of the scientific commitment (standard DRCD document) signed and dated by each investigator given to the representative of the promoter.

13.2 Methods for obtaining information and consent from research participants

In accordance with Article L1122-1-1 of the French Public Health Code, no biomedical research can be carried out on a person without free and informed consent, obtained in writing after the person has been given the information specified in Article L.1122-1 of said Code.

The subject will be granted a reflection period of **1 day minimum and 7 days maximum** between the time when the subject receives the information and the time when he or she signs the consent form.
The free and informed consent, in writing, of the subject is obtained by the investigator, or by a doctor representing the investigator, before the inclusion of the subject in the research. All pertinent aspects of the study must be explained to the patient before he or she signed the informed consent.

The information sheet and a copy of the consent form, signed and dated by the research subject and by the investigator or the doctor representing the investigator, are given to the individual prior to his or her participation in the research.

In addition, the investigator will specify in the research participant's medical file the methods used for obtaining his or her consent as well as the methods used for providing information with the goal of obtaining their consent. The investigator will retain the original signed and dated copy of the subject's consent form.

Special case: Mention of the possibility for the investigator of withholding certain information relating to the diagnosis, as applicable, in accordance with paragraph 4 of Article L1122-1 of the French Public Health Code.

Consent is given in writing. If it is not possible for the person solicited to consent in writing, the consent is certified by a third party. This third party must have no connection with the investigator or the administration manager.
In case of inability of the patient to sign the informed consent and no person of trust, continuation of care consent will be signed by the patient as soon as possible.

13.3 Subject prohibited from participating in another research or an exclusion period anticipated after the research, if applicable

During the time of participation to the research, the subject may not participate in other interventional research protocol unless the investigator considers that it's in the patient's interest to be included in another interventional research protocol.
The investigator must refer to the sponsor and the coordinating investigator before any inclusion in another research protocol and must mention the reasons in the medical file.

13.4 Compensation for subjects

No Compensation of is anticipated for the patients/control subjects as compensation for the inconveniences relating to the research.

13.5 Registration on the national register of subjects participating in biomedical research relating to the products listed in Article L. 5311-1 of the French Public Health Code

NA

13.6 Legal obligations

13.6.1 The sponsor's role

Assistance Publique - Hôpitaux de Paris (AP-HP) is the sponsor of this research and by delegation, the Clinical Research and Development Department (DRCD) carries out the research's missions in accordance with Article L.1121-1 of the French Public Health Code. Assistance Publique - Hôpitaux de Paris reserves the right to halt the research at any time for medical or administrative reasons. In this case, notification will be sent to the investigator

13.7 Request for an opinion from the Comité de Protection des Personnes (CPP, ethical review board)

AP-HP, as sponsor, obtains for this biomedical research relating to a medication for human use and prior to starting the research, the favourable opinion of the appropriate CPP, within the scope of its authority and in accordance with the legislative and regulatory provisions in force.

13.8 Request for authorisation to ANSM

AP-HP, as sponsor, obtains for this biomedical research relating to a medication for human use and prior to starting the research, authorisation from the ANSM, within the scope of its authority and in accordance with the legislative and regulatory provisions in force.

13.9 Commitment to compliance with the MR 001 "Méthodologie de Reference"

AP-HP, the research sponsor, has signed a commitment to comply with this "Méthodologie de reference".

13.10 Modifications to the research

Any substantial modification to the protocol by the coordinating investigator must be sent to the sponsor for approval. After approval is given, the sponsor must obtain, prior to starting the research, a favourable opinion from the CPP and authorisation from the ANSM within the scope of their respective authorities.
The information sheet and the consent form can be revised if necessary; in particular if there is a substantial modification to the research or if adverse reactions occur.

13.11 Final research report

The final biomedical research report referred to in Article R1123-60 of the French Public Health Code is drawn up and signed by the sponsor and the investigator. A summary of the report written according to the competent authority's reference plan will need to be sent to the competent authority and ethical review board within one year after the end of the research, meaning the end of the participation of the last research subject.

14 FUNDING AND INSURANCE

14.1 Funding source

PHRC:
The study was funded by a grant from Programme Hospitalier de Recherche Clinique en Cancérologie – PHRC-K 2015.

This research has been registered on the website http://clinicaltrials.gov/ under number registration number NCT 02876679

14.2 Insurance

For the duration of the research, the Sponsor will take out an insurance policy covering the sponsor's own civil liability as well as the civil liability of all the doctors involved in carrying out the research. The sponsor will also provide full compensation for all harmful consequences of the research for the research subjects and their beneficiaries, unless the sponsor can prove that the harm is not the fault of the sponsor or any agent. The act of a third party or the voluntary withdrawal of the person who initially consented to participate in the research cannot be invoked against said compensation.

Assistance Publique- Hôpitaux de Paris (AP-HP) has taken out insurance from HDI-GERLING through BIOMEDIC-INSURE for the full research period, covering its own civil liability and that of any agent (doctor or research staff), in accordance with Article L.1121-10 of the French Public Health Code.

15 PUBLICATION RULES

The steering committee will oversee the conduct of the trial, data analyses and publications.

15.1 Mention of the affiliation of AP-HP for projects sponsored or managed by AP-HP

AP-HP institution must appear under the symbol "AP-HP" first in the address followed precisely by AP-HP, hospital, department, city, postcode, France

15.2 Mention of the AP-HP manager (DRCD) in the acknowledgements of the text

The sponsor was Assistance Publique – Hôpitaux de Paris (Département de la Recherche Clinique et du Développement, Clinical Research and Development Department)"

15.3 Mention of the financier in the acknowledgements of the text

The research was funded by a grant from Programme Hospitalier de Recherche Clinique en Cancérologie – PHRC-K 2015 (Ministère de la Santé)

This research has been registered on the website http://clinicaltrials.gov/ under number *registration number 2016-002129-12*

16 BIBLIOGRAPHY

Adverse, N.C.I.C.T.C.f., at: http://ctep.cancer.gov/protocol, E.v.a.v.C.A., and Development/electronic_applications/ctc.htm. Accessed June 14.

Almog, S., Kurnik, D., Shimoni, A., Loebstein, R., Hassoun, E., Gopher, A., Halkin, H., and Nagler, A. (2011). Linearity and stability of intravenous busulfan pharmacokinetics and the role of glutathione in busulfan elimination. Biol Blood Marrow Transplant *17*, 117-123.

Baron, F., Labopin, M., Blaise, D., Lopez-Corral, L., Vigouroux, S., Craddock, C., Attal, M., Jindra, P., Goker, H., Socie, G.*, et al.* (2014). Impact of in vivo T-cell depletion on outcome of AML patients in first CR given peripheral blood stem cells and reduced-intensity conditioning allo-SCT from a HLA-identical sibling donor: a report from the Acute Leukemia Working Party of the European Group for Blood and Marrow Transplantation. Bone Marrow Transplant *49*, 389-396.

Baron, F., Labopin, M., Niederwieser, D., Vigouroux, S., Cornelissen, J.J., Malm, C., Vindelov, L.L., Blaise, D., Janssen, J.J., Petersen, E.*, et al.* (2012). Impact of graft-versus-host disease after reduced-intensity conditioning allogeneic stem cell transplantation for acute myeloid leukemia: a report from the Acute Leukemia Working Party of the European group for blood and marrow transplantation. Leukemia *26*, 2462-2468.

Bradstock, K.F., Bilmon, I., Kwan, J., Micklethwaite, K., Blyth, E., Deren, S., Bayley, A., Gebski, V., and Gottlieb, D. (2015). Single-Agent High-Dose Cyclophosphamide for Graft-versus-Host Disease Prophylaxis in Human Leukocyte Antigen-Matched Reduced-Intensity Peripheral Blood Stem Cell Transplantation Results in an Unacceptably High Rate of Severe Acute Graft-versus-Host Disease. Biol Blood Marrow Transplant *21*, 941-944.

Castagna, L., Crocchiolo, R., Furst, S., Bramanti, S., El Cheikh, J., Sarina, B., Granata, A., Mauro, E., Faucher, C., Mohty, B.*, et al.* (2014). Bone marrow compared with peripheral blood stem cells for haploidentical transplantation with a nonmyeloablative conditioning regimen and post-transplantation cyclophosphamide. Biol Blood Marrow Transplant *20*, 724-729.

Crocchiolo, R., Esterni, B., Castagna, L., Furst, S., El-Cheikh, J., Devillier, R., Granata, A., Oudin, C., Calmels, B., Chabannon, C.*, et al.* (2013). Two days of antithymocyte globulin are associated with a reduced incidence of acute and chronic graft-versus-host disease in reduced-intensity conditioning transplantation for hematologic diseases. Cancer *119*, 986-992.

Ganguly, S., Ross, D.B., Panoskaltsis-Mortari, A., Kanakry, C.G., Blazar, B.R., Levy, R.B., and Luznik, L. (2014). Donor CD4+ Foxp3+ regulatory T cells are necessary for posttransplantation cyclophosphamide-mediated protection against GVHD in mice. Blood *124*, 2131-2141.

Giralt, S., Estey, E., Albitar, M., van Besien, K., Rondon, G., Anderlini, P., O'Brien, S., Khouri, I., Gajewski, J., Mehra, R.*, et al.* (1997). Engraftment of allogeneic hematopoietic progenitor cells with purine analog-containing chemotherapy: harnessing graft-versus-leukemia without myeloablative therapy. Blood *89*, 4531-4536.

Gooley, T.A., Chien, J.W., Pergam, S.A., Hingorani, S., Sorror, M.L., Boeckh, M., Martin, P.J., Sandmaier, B.M., Marr, K.A., Appelbaum, F.R.*, et al.* (2010). Reduced mortality after allogeneic hematopoietic-cell transplantation. N Engl J Med *363*, 2091-2101.

Guillaume, T., Porcheron, S., Audat, F., Bancillon, N., Berceanu, A., Charbonnier, A., Dulery, R., Edy, N., El Cheikh, J., Hermet, E.*, et al.* (2014). [Prophylactic, preemptive and curative use of donor lymphocyte infusion in patients undergoing allogeneic stem cell transplantation: guidelines of the SFGM-TC]. Pathologie-biologie *62*, 193-196.

Holtick, U., Chemnitz, J.M., Shimabukuro-Vornhagen, A., Theurich, S., Chakupurakal, G., Krause, A., Fiedler, A., Luznik, L., Hellmich, M., Wolf, D.*, et al.* (2015). OCTET-CY: a phase II study to investigate the efficacy of post-transplant cyclophosphamide as sole graft-versus-host prophylaxis after allogeneic peripheral blood stem cell transplantation. Eur J Haematol.

Jagasia, M.H., Greinix, H.T., Arora, M., Williams, K.M., Wolff, D., Cowen, E.W., Palmer, J., Weisdorf, D., Treister, N.S., Cheng, G.S.*, et al.* (2015). National Institutes of Health Consensus Development Project on Criteria for Clinical Trials in Chronic Graft-versus-Host Disease: I. The 2014 Diagnosis and Staging Working Group report. Biol Blood Marrow Transplant *21*, 389-401.e381.

Kanakry, C.G., O'Donnell, P.V., Furlong, T., de Lima, M.J., Wei, W., Medeot, M., Mielcarek, M., Champlin, R.E., Jones, R.J., Thall, P.F.*, et al.* (2014). Multi-institutional study of post-transplantation cyclophosphamide as single-agent graft-versus-host disease prophylaxis after allogeneic bone marrow transplantation using myeloablative busulfan and fludarabine conditioning. J Clin Oncol *32*, 3497-3505.

Luznik, L., and Fuchs, E.J. (2010). High-dose, post-transplantation cyclophosphamide to promote graft-host tolerance after allogeneic hematopoietic stem cell transplantation. Immunologic research *47*, 65-77.
Luznik, L., O'Donnell, P.V., and Fuchs, E.J. (2012). Post-transplantation cyclophosphamide for tolerance induction in HLA-haploidentical bone marrow transplantation. Semin Oncol *39*, 683-693.
Luznik, L., O'Donnell, P.V., Symons, H.J., Chen, A.R., Leffell, M.S., Zahurak, M., Gooley, T.A., Piantadosi, S., Kaup, M., Ambinder, R.F.*, et al.* (2008). HLA-haploidentical bone marrow transplantation for hematologic malignancies using nonmyeloablative conditioning and high-dose, posttransplantation cyclophosphamide. Biol Blood Marrow Transplant *14*, 641-650.
Malard, F., Cahu, X., Clavert, A., Brissot, E., Chevallier, P., Guillaume, T., Delaunay, J., Ayari, S., Dubruille, V., Mahe, B.*, et al.* (2011). Fludarabine, antithymocyte globulin, and very low-dose busulfan for reduced-intensity conditioning before allogeneic stem cell transplantation in patients with lymphoid malignancies. Biol Blood Marrow Transplant *17*, 1698-1703.
Mamlouk, K., Saracino, G., Berryman, R.B., Fay, J.W., Pineiro, L.A., Vance, E.A., White, M., Sandler, I., and Agura, E.D. (2005). Modification of the Bu/Cy myeloablative regimen using daily parenteral busulfan: reduced toxicity without the need for pharmacokinetic monitoring. Bone Marrow Transplant *35*, 747-754.
Mayumi, H., Umesue, M., and Nomoto, K. (1996). Cyclophosphamide-induced immunological tolerance: an overview. Immunobiology *195*, 129-139.
McCurdy, S.R., Kanakry, J.A., Showel, M.M., Tsai, H.L., Bolanos-Meade, J., Rosner, G.L., Kanakry, C.G., Perica, K., Symons, H.J., Brodsky, R.A.*, et al.* (2015). Risk-stratified outcomes of nonmyeloablative HLA-haploidentical BMT with high-dose posttransplantation cyclophosphamide. Blood *125*, 3024-3031.
McSweeney, P.A., Niederwieser, D., Shizuru, J.A., Sandmaier, B.M., Molina, A.J., Maloney, D.G., Chauncey, T.R., Gooley, T.A., Hegenbart, U., Nash, R.A.*, et al.* (2001). Hematopoietic cell transplantation in older patients with hematologic malignancies: replacing high-dose cytotoxic therapy with graft-versus-tumor effects. Blood *97*, 3390-3400.
Mohty, M., Bay, J.O., Faucher, C., Choufi, B., Bilger, K., Tournilhac, O., Vey, N., Stoppa, A.M., Coso, D., Chabannon, C.*, et al.* (2003a). Graft-versus-host disease following allogeneic transplantation from HLA-identical sibling with antithymocyte globulin-based reduced-intensity preparative regimen. Blood *102*, 470-476.
Mohty, M., Jacot, W., Faucher, C., Bay, J.O., Zandotti, C., Collet, L., Choufi, B., Bilger, K., Tournilhac, O., Vey, N.*, et al.* (2003b). Infectious complications following allogeneic HLA-identical sibling transplantation with antithymocyte globulin-based reduced intensity preparative regimen. Leukemia *17*, 2168-2177.
Mohty, M., Malard, F., Abecassis, M., Aerts, E., Alaskar, A.S., Aljurf, M., Arat, M., Bader, P., Baron, F., Bazarbachi, A.*, et al.* (2016). Revised diagnosis and severity criteria for sinusoidal obstruction syndrome/veno-occlusive disease in adult patients: a new classification from the European Society for Blood and Marrow Transplantation. Bone Marrow Transplant.
Nomoto, K., Eto, M., Yanaga, K., Nishimura, Y., and Maeda, T. (1992). Interference with cyclophosphamide-induced skin allograft tolerance by cyclosporin A. J Immunol *149*, 2668-2674.
Osca-Gelis, G., Puig-Vives, M., Saez, M., Gallardo, D., Lloveras, N., and Marcos-Gragera, R. (2013). Population-based incidence of myeloid malignancies: fifteen years of epidemiological data in the province of Girona, Spain. Haematologica *98*, e95-97.
Peric, Z., Cahu, X., Chevallier, P., Brissot, E., Malard, F., Guillaume, T., Delaunay, J., Ayari, S., Dubruille, V., Le Gouill, S.*, et al.* (2011). Features of Epstein-Barr Virus (EBV) reactivation after reduced intensity conditioning allogeneic hematopoietic stem cell transplantation. Leukemia *25*, 932-938.
Pinana, J.L., Valcarcel, D., Fernandez-Aviles, F., Martino, R., Rovira, M., Barba, P., Martinez, C., Brunet, S., Sureda, A., Carreras, E.*, et al.* (2010). MTX or mycophenolate mofetil with CsA as GVHD prophylaxis after reduced-intensity conditioning PBSCT from HLA-identical siblings. Bone Marrow Transplant *45*, 1449-1456.
Przepiorka, D., Weisdorf, D., Martin, P., Klingemann, H.G., Beatty, P., Hows, J., and Thomas, E.D. (1995). 1994 Consensus Conference on Acute GVHD Grading. Bone Marrow Transplant *15*, 825-828.

Rubio, M.T., Labopin, M., Blaise, D., Socie, G., Contreras, R.R., Chevallier, P., Sanz, M.A., Vigouroux, S., Huynh, A., Shimoni, A., *et al.* (2015). The impact of graft-versus-host disease prophylaxis in reduced-intensity conditioning allogeneic stem cell transplant in acute myeloid leukemia: a study from the Acute Leukemia Working Party of the European Group for Blood and Marrow Transplantation. Haematologica *100*, 683-689.
Schwartz, R., and Dameshek, W. (1959). Drug-induced immunological tolerance. Nature *183*, 1682-1683.
Slavin, S., Nagler, A., Naparstek, E., Kapelushnik, Y., Aker, M., Cividalli, G., Varadi, G., Kirschbaum, M., Ackerstein, A., Samuel, S., *et al.* (1998). Nonmyeloablative stem cell transplantation and cell therapy as an alternative to conventional bone marrow transplantation with lethal cytoreduction for the treatment of malignant and nonmalignant hematologic diseases. Blood *91*, 756-763.
Smith, A., Crouch, S., Lax, S., Li, J., Painter, D., Howell, D., Patmore, R., Jack, A., and Roman, E. (2015). Lymphoma incidence, survival and prevalence 2004-2014: sub-type analyses from the UK's Haematological Malignancy Research Network. British journal of cancer *112*, 1575-1584.
Smith, A., Howell, D., Patmore, R., Jack, A., and Roman, E. (2011). Incidence of haematological malignancy by sub-type: a report from the Haematological Malignancy Research Network. British journal of cancer *105*, 1684-1692.
Soiffer, R.J., Lerademacher, J., Ho, V., Kan, F., Artz, A., Champlin, R.E., Devine, S., Isola, L., Lazarus, H.M., Marks, D.I., *et al.* (2011). Impact of immune modulation with anti-T-cell antibodies on the outcome of reduced-intensity allogeneic hematopoietic stem cell transplantation for hematologic malignancies. Blood *117*, 6963-6970.
Storb, R., Antin, J.H., and Cutler, C. (2010). Should methotrexate plus calcineurin inhibitors be considered standard of care for prophylaxis of acute graft-versus-host disease? Biol Blood Marrow Transplant *16*, S18-27.
Weisdorf, D., Zhang, M.J., Arora, M., Horowitz, M.M., Rizzo, J.D., and Eapen, M. (2012). Graft-versus-host disease induced graft-versus-leukemia effect: greater impact on relapse and disease-free survival after reduced intensity conditioning. Biol Blood Marrow Transplant *18*, 1727-1733.

17 LIST OF APPENDIX

Appendix 1: SUMMARY OF PRODUCT CHARACTERISTICS

- The SmPC of Endoxan is coming from the French public database of medicine (http://base-donnees-publique.medicaments.gouv.fr/)
- The SmPC of Thymoglobuline is coming from the French public database of medicine (http://base-donnees-publique.medicaments.gouv.fr/)

Appendix 2: GVHD STAGING (Przepiorka et al., 1995)

Stade	Skin	GI	Liver
1	< 25% rash	Diarrhea > 500 ml/24h or persistent nausea	Bilirubin : 10-30 mg/l
2	25-50% rash	Diarrhea > 1000 ml/24h	Bilirubin : 30-60 mg/l
3	> 50% rash	Diarrhea > 1500 ml/24h	Bilirubin : 60-150 mg/l
4	Generalized erythroderma with bullae	Large volume diarrhea and severe abdominal pain +/- ileus	Bilirubin : > 150 mg/l

Appendix 3: GVHD GRADING (Przepiorka et al., 1995)

Grade	Skin	GI	Liver
I	Stage 1 - 2	0	0
II	Stage 1 - 3	Stage 1	Stage1
III	Stage 2 à 3	Stage >2	Stage >2
IV	Stage ≥2	Stage ≥2	Stage ≥2

Appendix 4: Rule of 9s for body surface area.

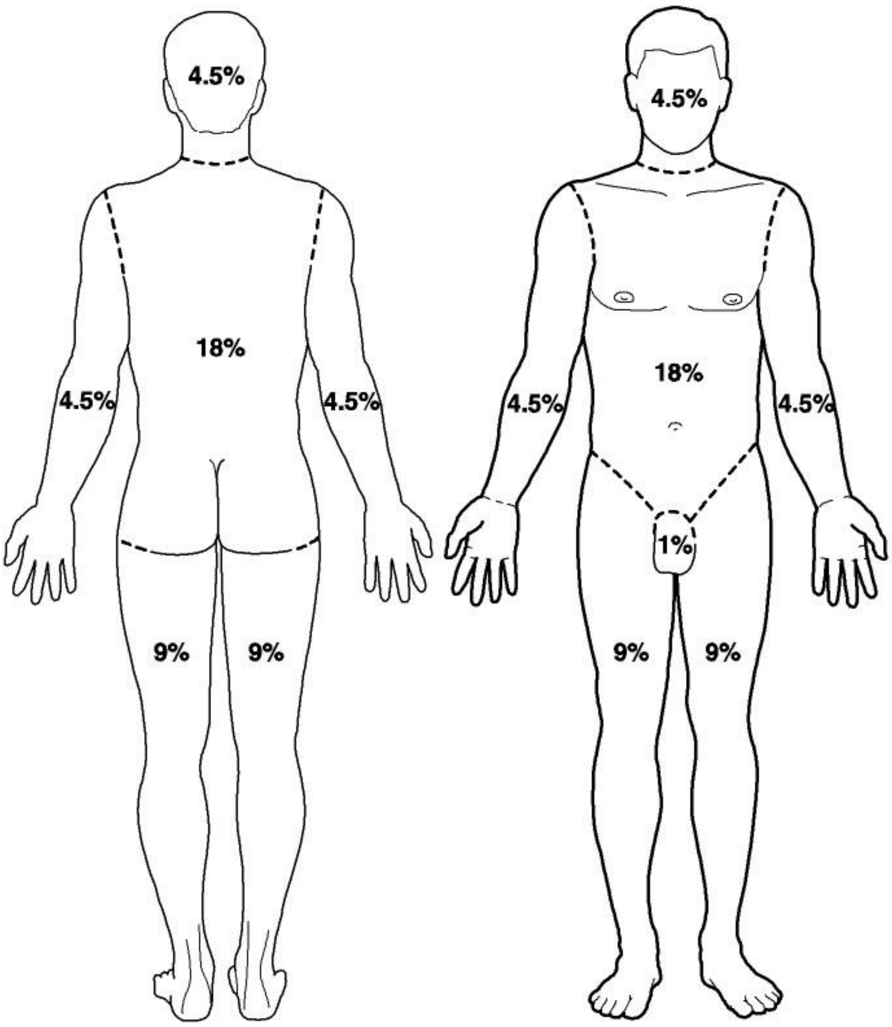
4.5%
18%
4.5%
4.5%
9%
9%
4.5%
18%
4.5%
4.5%
1%
9%
9%

Appendix 5: Signs and symptoms of cGvHD (2014 NIH consensus criteria) (Jagasia et al., 2015)

Signs and Symptoms of chronic GVHD

Organ or Site	Diagnostic (Sufficient to Establish the Diagnosis of chronic GVHD)	Distinctive[*] (Seen in chronic GVHD, but Insufficient Alone to Establish a Diagnosis)	Other Features or Unclassified Entities[†]	Common[‡] (Seen with Both Acute and chronic GVHD)
Skin	Poikiloderma<br>Lichen planus–like features<br>Sclerotic features Morphea-like features<br>Lichen sclerosus–like features	Depigmentation<br>Papulosquamous lesions	Sweat impairment<br>Ichthyosis<br>Keratosis pilaris<br>Hypopigmentation<br>Hyperpigmentation	Erythema<br>Maculopapular rash<br>Pruritus
Nails		Dystrophy<br>Longitudinal ridging, splitting or brittle features<br>Onycholysis<br>Pterygium unguis<br>Nail loss (usually symmetric, affects most nails)		
Scalp and body hair		New onset of scarring or nonscarring scalp alopecia (after recovery from chemoradiotherapy)<br>Loss of body hair<br>Scaling	Thinning scalp hair, typically patchy, coarse or dull (not explained by endocrine or other causes)<br>Premature gray hair	
Mouth	Lichen planus–like changes	Xerostomia<br>Mucoceles<br>Mucosal atrophy<br>Ulcers<br>Pseudomembranes		Gingivitis<br>Mucositis<br>Erythema<br>Pain
Eyes		New onset dry, gritty, or painful eyes<br>Cicatricial conjunctivitis<br>KCS<br>Confluent areas of punctate keratopathy	Photophobia<br>Periorbital hyperpigmentation<br>Blepharitis (erythema of the eyelids with edema)	
Genitalia	Lichen planus–like features<br>Lichen sclerosus–like features	Erosions<br>Fissures		
Females	Vaginal scarring or clitoral/labial agglutination	Ulcers		
Males	Phimosis or urethral/meatus scarring or stenosis			
GI Tract	Esophageal web<br>Strictures or stenosis in the upper to mid third of the esophagus		Exocrine pancreatic insufficiency	Anorexia<br>Nausea<br>Vomiting<br>Diarrhea<br>Weight loss<br>Failure to thrive (infants and children
Liver				Total bilirubin, alkaline phosphatase $>2\times$ upper limit of normal<br>ALT $>2\times$ upper limit of normal
Lung	Bronchiolitis obliterans diagnosed with lung biopsy<br>BOS[§]	Air trapping and bronchiectasis on chest CT	Cryptogenic organizing pneumonia<br>Restrictive lung disease[‖]	
Muscles, fascia, joints	Fasciitis<br>Joint stiffness or contractures secondary to fasciitis or sclerosis	Myositis or polymyositis[¶]	Edema<br>Muscle cramps<br>Arthralgia or arthritis	
Hematopoietic and Immune			Thrombocytopenia<br>Eosinophilia<br>Lymphopenia<br>Hypo- or hyper-gammaglobulinemia<br>Autoantibodies (AIHA, ITP)<br>Raynaud's phenomenon	
Other			Pericardial or pleural effusions<br>Ascites<br>Peripheral neuropathy<br>Nephrotic syndrome<br>Myasthenia gravis<br>Cardiac conduction abnormality or cardiomyopathy	

ALT indicates alanine aminotransferase; AIHA, autoimmune hemolytic anemia; ITP, idiopathic thrombocytopenic purpura.

[*] In all cases, infection, drug effect, malignancy, or other causes must be excluded.
[†] Can be acknowledged as part of the chronic GVHD manifestations if diagnosis is confirmed.
[‡] Common refers to shared features by both acute and chronic GVHD.
[§] BOS can be diagnostic for lung chronic GVHD only if distinctive sign or symptom present in another organ (see text).
[‖] Pulmonary entities under investigation or unclassified.
[¶] Diagnosis of chronic GVHD requires biopsy.

Appendix 6: Organ scoring of cGvHD (2014 NIH consensus criteria) (Jagasia et al., 2015)

	SCORE 0	SCORE 1	SCORE 2	SCORE 3
**PERFORMANCE SCORE:** ☐ **KPS ECOG LPS**	☐ Asymptomatic and fully active (ECOG 0; KPS or LPS 100%)	☐ Symptomatic, fully ambulatory, restricted only in physically strenuous activity (ECOG 1, KPS or LPS 80-90%)	☐ Symptomatic, ambulatory, capabl of self-care, >50% of waking hours ou of bed (ECOG 2, KPS or LPS 60-70%)	☐ Symptomatic, limited self-care, >50% of waking hours in bed (ECOG 3-4, KPS or LPS <60%)
**SKIN†** **SCORE % BSA** ☐ *GVHD features to be scored by BSA*: **Check all that apply**: ☐ Maculopapular rash/erythema ☐ Lichen planus-like features ☐ Sclerotic features ☐ Papulosquamous lesions or ichthyosis ☐ Keratosis pilaris-like GVHD	☐ No BSA involved	☐ 1-18% BSA	☐ 19-50% BSA	☐ >50% BSA
**SKIN FEATURES SCORE:**	☐ No sclerotic features		☐ Superficial sclerotic features "not hidebound" (able to pinch)	**Check all that apply**: ☐ Deep sclerotic features ☐ "Hidebound" (unable to pinch) ☐ Impaired mobility ☐ Ulceration
Other skin GVHD features (NOT scored by BSA) **Check all that apply**: ☐ Hyperpigmentation ☐ Hypopigmentation ☐ Poikiloderma ☐ Severe or generalized pruritus ☐ Hair involvement ☐ Nail involvement ☐ *Abnormality present but explained entirely by non-GVHD documented cause (specify):*______________________________				
**MOUTH** *Lichen planus-like features present:* ☐ **Yes** ☐ **No**	☐ No symptoms	☐ Mild symptoms **with** disease signs but not limiting oral intake significantly	☐ Moderate symptoms with disease signs **with** partial limitation of oral intake	☐ Severe symptoms with disease signs on examination **with** major limitation of oral intake
☐ *Abnormality present but explained entirely by non-GVHD documented cause (specify):*______________________________				

Figure 1. Organ scoring of chronic GVHD. ECOG indicates Eastern Cooperative Oncology Group; KPS, Karnofsky Performance Status; LPS, Lansky Performance Status; BSA, body surface area; ADL, activities of daily living; LFTs, liver function tests; AP, alkaline phosphatase; ALT, alanine aminotransferase; ULN, normal upper limit. *Weight loss within 3 months. †Skin scoring should use both percentage of BSA involved by disease signs and the cutaneous features scales. When a discrepancy exists between the percentage of total body surface (BSA) score and the skin feature score, OR if superficial sclerotic features are present (Score 2), but there is impaired mobility or ulceration (Score 3), the higher level should be used for the final skin scoring. ‡To be completed by specialist or trained medical providers (see Supplemental Figure). **Lung scoring should be performed using both the symptoms and FEV1 scores whenever possible. FEV1 should be used in the final lung scoring where there is discrepancy between symptoms and FEV1 scores.

	SCORE 0	SCORE 1	SCORE 2	SCORE 3
**EYES**<br><br>*Keratoconjunctivitis sicca (KCS) confirmed by ophthalmologist:*<br>☐ **Yes**<br>☐ **No**<br>☐ **Not examined**	☐ No symptoms	☐ Mild dry eye symptoms not affecting ADL (requirement of lubricant eye drops ≤ 3 x per day)	☐ Moderate dry eye symptoms partially affecting ADL (requiring lubricant eye drops > 3 x per day or punctal plugs), **WITHOUT** new vision impairment due to KCS	☐ Severe dry eye symptoms significantly affecting ADL (special eyeware to relieve pain) **OR** unable to work because of ocular symptoms **OR** loss of vision due to KCS
☐ *Abnormality present but explained entirely by non-GVHD documented cause (specify):*______________________________				
**GI Tract**<br>***Check all that apply:***<br>☐ Esophageal web/ proximal stricture or ring<br>☐ Dysphagia<br>☐ Anorexia<br>☐ Nausea<br>☐ Vomiting<br>☐ Diarrhea<br>☐ Weight loss ≥5%*<br>☐ Failure to thrive	☐ No symptoms	☐ Symptoms without significant weight loss* (<5%)	☐ Symptoms associated with mild to moderate weight loss* (5-15%) **OR** moderate diarrhea without significant interference with daily living	☐ Symptoms associated with significant weight loss* >15%, requires nutritional supplement for most calorie needs **OR** esophageal dilation **OR** severe diarrhea with significant interference with daily living
☐ *Abnormality present but explained entirely by non-GVHD documented cause (specify):*______________________________				
**LIVER**	☐ Normal total bilirubin and ALT or AP < 3 x ULN	☐ Normal total bilirubin with ALT ≥3 to 5 x ULN or AP ≥ 3 x ULN	☐ Elevated total bilirubin but ≤3 mg/dL or ALT > 5 ULN	☐ Elevated total bilirubin > 3 mg/dL
☐ *Abnormality present but explained entirely by non-GVHD documented cause (specify):*				
**LUNGS****<br>__Symptom score__:	☐ No symptoms	☐ Mild symptoms (shortness of breath after climbing one flight of steps)	☐ Moderate symptoms (shortness of breath after walking on flat ground)	☐ Severe symptoms (shortness of breath at rest; requiring 0_2)
__**Lung score**__:<br>**%** FEV1 ☐<br><br>*Pulmonary function tests*<br>☐ Not performed	☐ FEV1≥80%	☐ FEV1 60-79%	☐ FEV1 40-59%	☐ FEV1 ≤39%
☐ *Abnormality present but explained entirely by non-GVHD documented cause (specify):*______________________________				

	SCORE 0	SCORE 1	SCORE 2	SCORE 3
**JOINTS AND FASCIA**<br><br>P-ROM score<br>*(see below)*<br>Shoulder (1-7):___<br>Elbow (1-7): ____<br>Wrist/finger (1-7):___<br>Ankle (1-4):___	☐ No symptoms	☐ Mild tightness of arms or legs, normal or mild decreased range of motion (ROM) **AND** not affecting ADL	☐ Tightness of arms or legs **OR** joint contractures, erythema thought due to fasciitis, moderate decrease ROM **AND** mild to moderate limitation of ADL	☐ Contractures **WITH** significant decrease of ROM ***AND*** significant limitation of ADL (unable to tie shoes, button shirts, dress self etc.)
☐ *Abnormality present but explained entirely by non-GVHD documented cause (specify):*___				
**GENITAL TRACT**<br>(*See Supplemental figure*[‡])<br>☐ Not examined<br>*Currently sexually active*<br>☐ Yes<br>☐ No	☐ No signs	☐ Mild signs[‡] and females with or without discomfort on exam	☐ Moderate signs[‡] and may have symptoms with discomfort on exam	☐ Severe signs[‡] with or without symptoms
☐ *Abnormality present but explained entirely by non-GVHD documented cause (specify):*___				

Other indicators, clinical features or complications related to chronic GVHD (check all that apply and assign a score to severity (0-3) based on functional impact where applicable none – 0,mild -1, moderate -2, severe – 3)

☐ Ascites (serositis)___	☐ Myasthenia Gravis___	
☐ Pericardial Effusion___	☐ Peripheral Neuropathy___	☐ Eosinophilia > 500/µl___
☐ Pleural Effusion(s)___	☐ Polymyositis___	☐ Platelets <100,000/µl ___
☐ Nephrotic syndrome___	☐ Weight loss>5%* without GI symptoms ___	☐ Others (specify):___

**Overall GVHD Severity**<br>*(Opinion of the evaluator)*	❑ **No GVHD**	❑ **Mild**	❑ **Moderate**	❑ **Severe**

Photographic Range of Motion (P-ROM)

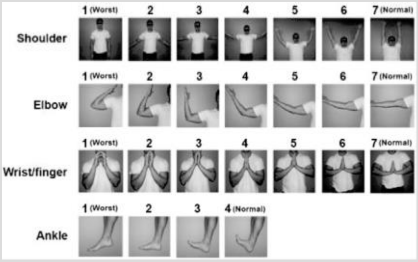

Appendix 7: Performans status (OMS)

0 - Asymptomatic

1 - symptomatic, fully ambulatory

2 - symptomatic, in bed < 50 % of day.

3 - symptomatic, in bed > 50 % of day but not bedridden.

4 - Bedridden.

Appendix 8: Karnofsky Performance Scale Index

Able to carry on normal activity and to work; no special care needed.

100 - Normal no complaints; no evidence of disease.

90 - Able to carry on normal activity; minor signs or symptoms of disease.

80 - Normal activity with effort; some signs or symptoms of disease.

Unable to work; able to live at home and care for most personal needs; varying amount of assistance needed.

70 - Cares for self; unable to carry on normal activity or to do active work.

60 - Requires occasional assistance, but is able to care for most of his personal needs.

50 - Requires considerable assistance and frequent medical care.

Unable to care for self; requires equivalent of institutional or hospital care; disease may be progressing rapidly.

40 - Disabled; requires special care and assistance.

30 - Severely disabled; hospital admission is indicated although death not imminent.

20 - Very sick; hospital admission necessary; active supportive treatment necessary.

10 - Moribund; fatal processes progressing rapidly.

0 - Dead

Appendix 9: EORTC QLQ-C30 (European Organisation for Research and Treatment of Cancer Quality of Life Questionnaire-Core 30)

FRENCH (EUROPE)

EORTC QLQ-C30 (version 3)

Nous nous intéressons à vous et à votre santé. Répondez vous-même à toutes les questions en entourant le chiffre qui correspond le mieux à votre situation. Il n'y a pas de « bonne » ou de « mauvaise » réponse. Ces informations sont strictement confidentielles.

Merci de préciser :

Vos initiales : |_|_|_|_|
Date de naissance (jour/mois/année) : |_|_|_|_|_|_|_|_|
La date d`aujourd`hui (jour/mois/année) : 31 |_|_|_|_|_|_|_|_|

	Pas du tout	Un peu	Assez	Beaucoup
1. Avez-vous des difficultés à faire certains efforts physiques pénibles comme porter un sac à provisions chargé ou une valise ?	1	2	3	4
2. Avez-vous des difficultés à faire une <u>longue</u> promenade ?	1	2	3	4
3. Avez-vous des difficultés à faire un <u>petit</u> tour dehors ?	1	2	3	4
4. Êtes-vous obligé(e) de rester au lit ou dans un fauteuil pendant la journée ?	1	2	3	4
5. Avez-vous besoin d'aide pour manger, vous habiller, faire votre toilette ou aller aux toilettes ?	1	2	3	4

**Au cours de la semaine passée :**	Pas du tout	Un peu	Assez	Beaucoup
6. Avez-vous été gêné(e) pour faire votre travail ou vos activités de tous les jours ?	1	2	3	4
7. Avez-vous été gêné(e) dans vos activités de loisirs ?	1	2	3	4
8. Avez-vous eu le souffle court ?	1	2	3	4
9. Avez-vous ressenti de la douleur ?	1	2	3	4
10. Avez-vous eu besoin de repos ?	1	2	3	4
11. Avez-vous eu des difficultés à dormir ?	1	2	3	4
12. Vous êtes-vous senti(e) faible ?	1	2	3	4
13. Avez-vous manqué d'appétit ?	1	2	3	4
14. Avez-vous eu des nausées (mal au cœur) ?	1	2	3	4
15. Avez-vous vomi ?	1	2	3	4

<u>Passez à la page suivante S.V.P.</u>

**Au cours de la semaine passée :**	**Pas du tout**	**Un peu**	**Assez**	**Beaucoup**
16. Avez-vous été constipé(e) ?	1	2	3	4
17. Avez-vous eu de la diarrhée ?	1	2	3	4
18. Étiez-vous fatigué(e) ?	1	2	3	4
19. Des douleurs ont-elles perturbé vos activités quotidiennes ?	1	2	3	4
20. Avez-vous eu des difficultés à vous concentrer sur certaines choses, par exemple, pour lire le journal ou regarder la télévision ?	1	2	3	4
21. Vous êtes-vous senti(e) tendu(e) ?	1	2	3	4
22. Vous êtes-vous fait du souci ?	1	2	3	4
23. Vous êtes-vous senti(e) irritable ?	1	2	3	4
24. Vous êtes-vous senti(e) déprimé(e) ?	1	2	3	4
25. Avez-vous eu des difficultés à vous souvenir de certaines choses ?	1	2	3	4
26. Votre état physique ou votre traitement médical vous ont-ils gêné(e) dans votre vie familiale ?	1	2	3	4
27. Votre état physique ou votre traitement médical vous ont-ils gêné(e) dans vos activités sociales (par exemple, sortir avec des amis, aller au cinéma...) ?	1	2	3	4
28. Votre état physique ou votre traitement médical vous ont-ils causé des problèmes financiers ?	1	2	3	4

Pour les questions suivantes, veuillez répondre en entourant le chiffre entre 1 et 7 qui s'applique le mieux à votre situation

29. Comment évalueriez-vous votre état de santé au cours de la semaine passée ?

1 2 3 4 5 6 7

Très mauvais Excellent

30. Comment évalueriez-vous l'ensemble de votre qualité de vie au cours de la semaine passée ?

1 2 3 4 5 6 7

Très mauvaise Excellente

© QLQ-C30 Copyright 1995 EORTC Quality of Life Group. Tous droits réservés. Version 3.0

Appendix 10: FACT-BMT (Functional Assessment of Cancer Therapy - Bone Marrow Transplant)

FACT-BMT (4ème Version)

Veuillez indiquer votre réponse en entourant un seul chiffre par ligne et en tenant compte des <u>7 derniers jours</u>.

	AUTRES SUJETS D'INQUIÉTUDE	Pas du tout	Un peu	Moyen-nement	Beau-coup	Énormé-ment
BMT1	Je m'inquiète de ne pas pouvoir continuer à travailler (y compris le travail à la maison)	0	1	2	3	4
BMT2	Je me sens distant(e) des autres	0	1	2	3	4
BMT3	J'ai peur que la greffe ne réussisse pas	0	1	2	3	4
BMT4	Les effets du traitement sont pires que ce que j'imaginais	0	1	2	3	4
C6	J'ai bon appétit	0	1	2	3	4
C7	Je suis satisfait(e) de mon apparence physique	0	1	2	3	4
BMT5	Je peux me débrouiller seul(e)	0	1	2	3	4
BMT6	Je me fatigue facilement	0	1	2	3	4
BL4	Le sexe m'intéresse	0	1	2	3	4
BMT7	J'ai peur de ne plus pouvoir avoir d'enfants	0	1	2	3	4
BMT8	J'ai confiance en mes infirmières(iers)	0	1	2	3	4
BMT9	Je regrette d'avoir eu une greffe de la moelle osseuse	0	1	2	3	4
BMT 10	J'ai de la mémoire	0	1	2	3	4
Br1	Je suis capable de me concentrer	0	1	2	3	4
BMT 11	J'ai fréquemment des rhumes ou des infections	0	1	2	3	4
BMT 12	Je vois trouble	0	1	2	3	4
BMT 13	Je suis gêné(e) par un changement de goût des aliments	0	1	2	3	4
BMT 14	J'ai des tremblements	0	1	2	3	4
B1	J'ai le souffle court	0	1	2	3	4
BMT 15	Je suis gêné(e) par des problèmes de peau (éruptions démangeaisons)	0	1	2	3	4
BMT 16	J'ai du mal à aller à la selle	0	1	2	3	4
BMT 17	Ma maladie est une lourde épreuve pour ma famille proche	0	1	2	3	4
BMT 18	Le coût du traitement est un fardeau pour moi et pour ma famille	0	1	2	3	4

French (Universal)
Copyright 1987, 1997

FACT-BMT (4ème Version)

Vous trouverez ci-dessous une liste de commentaires que d'autres personnes atteintes de la même maladie que vous ont jugés importants. **Veuillez indiquer votre réponse en entourant un seul chiffre par ligne et en tenant compte des 7 derniers jours.**

	**BIEN-ÊTRE PHYSIQUE**	**Pas du tout**	**Un peu**	**Moyen-nement**	**Beau-coup**	**Énormé-ment**
GP1	Je manque d'énergie	0	1	2	3	4
GP2	J'ai des nausées	0	1	2	3	4
GP3	À cause de mon état physique, j'ai du mal à répondre aux besoins de ma famille	0	1	2	3	4
GP4	J'ai des douleurs	0	1	2	3	4
GP5	Je suis incommodé(e) par les effets secondaires du traitement	0	1	2	3	4
GP6	Je me sens malade	0	1	2	3	4
GP7	Je suis obligé(e) de passer du temps allongé(e)	0	1	2	3	4

	**BIEN-ÊTRE FAMILIAL/SOCIAL**	**Pas du tout**	**Un peu**	**Moyen-nement**	**Beau-coup**	**Énormé-ment**
GS1	Je me sens proche de mes amis	0	1	2	3	4
GS2	Ma famille me soutient moralement	0	1	2	3	4
GS3	Mes amis me soutiennent	0	1	2	3	4
GS4	Ma famille a accepté ma maladie	0	1	2	3	4
GS5	Je suis satisfait(e) de la communication avec ma famille au sujet de ma maladie	0	1	2	3	4
GS6	Je me sens proche de mon (ma) partenaire (ou de la personne qui est mon principal soutien)	0	1	2	3	4
Q1	*Quel que soit votre degré d'activité sexuelle en ce moment, veuillez répondre à la question suivante. Si vous préférez ne pas y répondre, cochez cette case et passez à la section suivante.* ☐					
GS7	Je suis satisfait(e) de ma vie sexuelle	0	1	2	3	4

French (Universal)
Copyright 1987, 1997

17 February 2010
Page 1 of 3

FACT-BMT (4ème Version)

Veuillez indiquer votre réponse en entourant un seul chiffre par ligne et en tenant compte des 7 derniers jours.

	**BIEN-ÊTRE ÉMOTIONNEL**	**Pas du tout**	**Un peu**	**Moyen-nement**	**Beau-coup**	**Énormé-ment**
GE1	Je me sens triste...	0	1	2	3	4
GE2	Je suis satisfait(e) de la façon dont je fais face à ma maladie...	0	1	2	3	4
GE3	Je perds espoir dans le combat contre ma maladie.........	0	1	2	3	4
GE4	Je me sens nerveux (nerveuse)......................................	0	1	2	3	4
GE5	Je suis préoccupé(e) par l'idée de mourir.......................	0	1	2	3	4
GE6	Je suis préoccupé(e) à l'idée que mon état de santé puisse s'aggraver...	0	1	2	3	4

	**BIEN-ÊTRE FONCTIONNEL**	**Pas du tout**	**Un peu**	**Moyen-nement**	**Beau-coup**	**Énormé-ment**
GF1	Je suis capable de travailler (y compris le travail à la maison)...	0	1	2	3	4
GF2	Mon travail (y compris le travail à la maison) me donne de la satisfaction...	0	1	2	3	4
GF3	Je suis capable de profiter de la vie................................	0	1	2	3	4
GF4	J'ai accepté ma maladie..	0	1	2	3	4
GF5	Je dors bien..	0	1	2	3	4
GF6	J'apprécie mes loisirs habituels......................................	0	1	2	3	4
GF7	Je suis satisfait(e) de ma qualité de vie actuelle.............	0	1	2	3	4

Copyright 1987, 1997
