# Supplementary material for: Post-transplant cyclophosphamide versus anti-thymocyte globulin after reduced intensity peripheral blood allogeneic cell transplantation in recipients of matched sibling or 10/10 HLA matched unrelated donors: final analysis of a randomized, open-label, multicenter, phase 2 trial
Source: Blood Cancer J. 2024 Feb 19;14(1):31. doi: 10.1038/s41408-024-00990-3 (PMC10876658; doi:10.1038/s41408-024-00990-3)
Supplement: Supplementary file 2 — supplementary appendix 2 [file 41408_2024_990_MOESM2_ESM.pdf]

Table S1. Outcomes of the 8 patients who dropped out in the ATG arm

n°	Reason for drop out	allo-HCT	Date of allo-HCT	GVHD	Relapse	Last status (time from randomization)	Cause of death
1	relapse 19 days after randomization	No			Yes (day 19 post randomization)	death day 91	relapse/progression
2	Donor not available => haploidentical donor chosen	Yes	09/29/2017		No	alive day 1573	
3	relapse 1 day after randomization	No			Yes (day 1 post randomization)	death day 63	relapse/progression
4	molecular relapse day 15 before allo-HCT	Yes	03/07/2018 (sequential conditioning regimen)	GVH skin grade II	Yes (day 991 post randomization)	death day 1013	relapse/progression
5	molecular relapse before allo-HCT	Yes	10/25/2017 (sequential conditioning regimen)		Yes (day 83 post randomization)	death day 226	relapse/progression
6	relapse 13 days after randomization	No			Yes (day 13 after randomization)	death day 421	relapse/progression
7	donor has EBV replication	Yes	01/29/2019	GVHD skin & gut	No	death day 867	infection (Covid)
8	relapse 1 day after randomization	Yes	08/01/2017 (sequential conditioning regimen)	No	Yes (day 64 after randomization)	death day 73	relapse/progression

Table S2: Acute GVHD, extent of organ involvement

	Stage	PTCy, n (%)	ATG, n (%)	p value
Skin	0	19 (43.2%)	22 (59.5%)	0.43
	1	10 (22.7%)	5 (13.5%)	
	2	11 (25.0%)	6 (16.2%)	
	3	4 (9.10%)	4 (10.8%)	
Liver	0	40 (90.9%)	34 (91.9%)	0.69
	1	2 (4.5%)	1 (2.7%)	
	2	1 (2.3%)	1 (2.7%)	
	3	1 (2.3%)	0 (0%)	
	4	0 (0%)	1 (2.7%)	
Gut	0	36 (81.8%)	32 (86.5%)	0.45
	1	3 (6.8%)	3 (8.1%)	
	2	2 (4.5%)	0 (0%)	
	3	3 (6.8%)	1 (2.7%)	
	4	0 (0%)	1 (2.7%)	

abbreviations: GVHD: graft-versus-host disease, PTCy: post-transplant cyclophosphamide, ATG: anti-thymocyte globulin

Table S3. Cause of death

**Cause of death**	N
Relapse	16
GVHD	3
Infection	6
Hemorrhage	2
VOD/SOS	1
Other (1 mesenteric ischemia, 1 melanoma)	2
Total	30
Missing	1

Abbreviations: GVHD: graft-versus-host disease, VOD/SOS: Hepatic veno-occlusive disease/sinusoidal obstruction syndrome
